# A novel bioresponsive self-immolative spacer based on aza-quinone methide reactivity for the controlled release of thiols, phenols, amines, sulfonamides or amides†

**DOI:** 10.1039/d4sc01576b

**Published:** 2024-04-02

**Authors:** Elena Ermini, Annalaura Brai, Elena Cini, Federica Finetti, Giuseppe Giannini, Daniele Padula, Lucrezia Paradisi, Federica Poggialini, Lorenza Trabalzini, Paola Tolu, Maurizio Taddei

**Affiliations:** a Dipartimento di Biotecnologie, Chimica e Farmacia, Università degli Studi di Siena Via A. Moro 2 53100 Siena Italy maurizio.taddei@unisi.it; b Translational Medicine & Clinical Pharmacology Corporate R&D – Alfasigma SpA Via Pontina, km 30400 00071 Pomezia (Roma) Italy

## Abstract

A stimuli-sensitive linker is one of the indispensable components of prodrugs for cancer therapy as it covalently binds the drug and releases it upon external stimulation at the tumour site. Quinone methide elimination has been widely used as the key transformation to release drugs based on their nucleofugacity. The usual approach is to bind the drug to the linker as a carbamate and release it as a free amine after a self-immolative 1,6-elimination. Although this approach is very efficient, it is limited to amines (as carbamates), alcohols or phenols (as carbonates) or other acidic functional groups. We report here a self-immolative spacer capable of directly linking and releasing amines, phenols, thiols, sulfonamides and carboxyamides after a reductive stimulus. The spacer is based on the structure of (5-nitro-2-pyrrolyl)methanol (NPYM-OH), which was used for the direct alkylation of the functional groups mentioned above. The spacer is metabolically stable and has three indispensable sites for bioconjugation: the bioresponsive trigger, the conjugated 1,6 self-immolative system and a third arm suitable for conjugation with a carrier or other modifiers. Release was achieved by selective reduction of the nitro group over Fe/Pd nanoparticles (NPs) in a micellar aqueous environment (H_2_O/TPGS-750-M), or by NADH mediated nitroreductase activation. A DFT study demonstrates that, during the 1,6 elimination, the transition state formed from 5-aminopyrrole has a lower activation energy compared to other 5-membered heterocycles or *p*-aminobenzyl derivatives. The NPYM scaffold was validated by late-stage functionalisation of approved drugs such as celecoxib, colchicine, vorinostat or ciprofloxacin. A hypoxia-activated NPYM-based prodrug (HAP) derived from HDAC inhibitor ST7612AA1 was also produced, which was active in cancer cells under hypoxic conditions.

## Abstract 2

The power of pyrrole: a 1,6-self-immolative spacer designed on the pyrrole structure enables direct late-stage functionalisation of drugs and their traceless release under enzymatic or reductive micellar catalysis in water.

## Graphical abstract

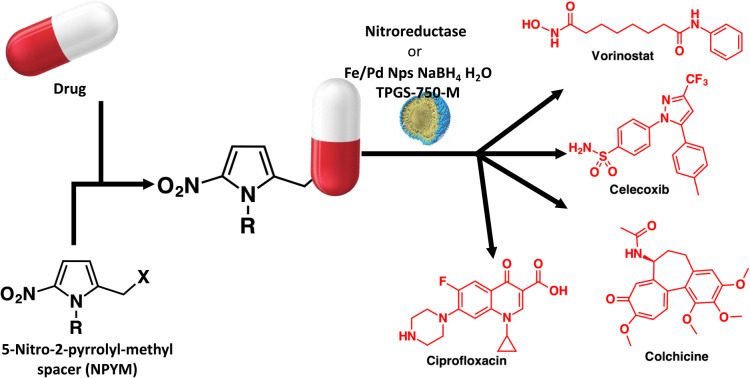

## Introduction

Molecular architectures that change their chemical or physical properties in response to various external stimuli have found application in several new areas of organic chemistry. Organic materials,^1^ polymers,^2^ fluorophore probes,^3^ toll systems for chemical biology^4^ and new drugs^5^ all benefit from stimuli-responsive self-immolative disassembly that enables signal amplification.^5*c*,6^ In the prodrug field, various scaffolds equipped with external stimuli triggers have been used in target delivery systems to monitor and control the release of drug molecules.

**Scheme 1:**
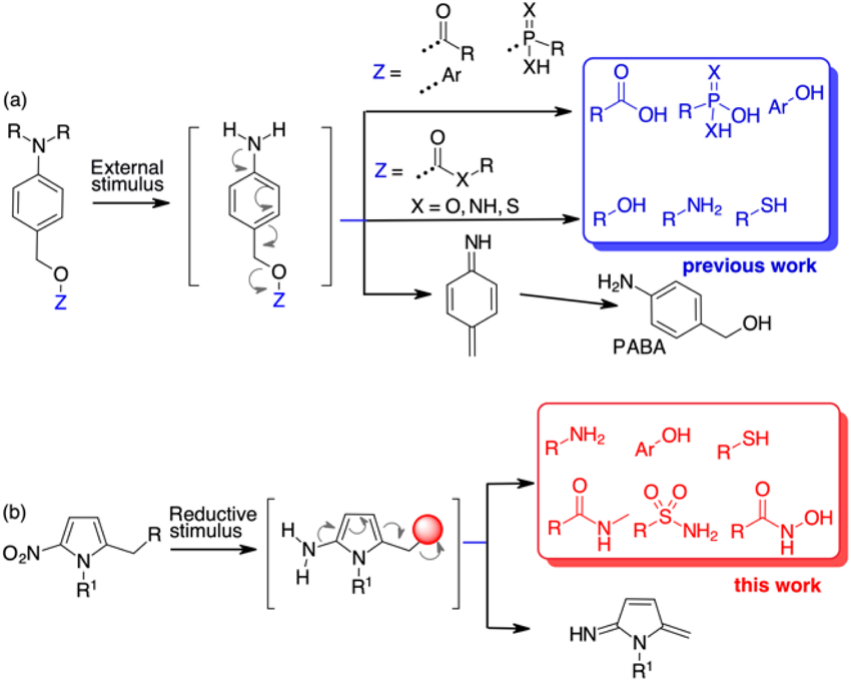
State of the art for 1,6-self-immolative spacers (a) and results of this paper (b).

Quinone methide elimination has been used for many years as a unique adaptor to control the self-immolative properties of stimulus-responsive systems.^7^ Molecular adaptors based on quinone or aza-quinone methide chemistry behave like stable spacers between a reactive group and a reporter moiety and can undergo 1,4-, 1,6- or 1,8-type elimination reactions upon pulling the trigger.^8^ The result is the formation of a quinone methide species and the release of the reporter group.^9^ Using *p*-aminobenzyl alcohol (PABA) derivatives, when the appropriate stimulus generates the free amine, a 1,6-electron cascade occurs that releases the fragment bound at the benzylic position (Scheme 1a). However, this self-immolative process relies on molecules containing functional groups that are characterised by high nucleofugacity, *i.e.* have a p*K*_a_ ≤ 9.0 (Scheme 1a).^10^ While carboxylic, sulfonic or phosphonic acids and “acidic” phenols can be bound directly to the PABA-like spacer and are released as soon as the free amine is formed, less acidic compounds such as amines are bound to the benzyl spacer as carbamates. The further uncaged process releases the free amine after decarboxylation (Scheme 1a). Alcohols can be bound as carbonates while alternative approaches are based on 1,6 self-immolation followed by cyclization release.^11^ Other tailor-made linkers for alcohols are based on polarity or pH variations.^12^ The use of PABA linkers to release β-lapachone-like molecules through a benzylic C–C bond cleavage has also been reported.^13^ However, reporting molecules and, more importantly, drugs that do not contain sufficient acid groups, cannot be used in release processes controlled by 1,6 self-immolative mechanism.

With the aim of increasing the number of tools for self-immolative processes, we decided to investigate the possibility of using a heteroaromatic scaffold as the 1,6-self-immolative adaptor for the direct conjugation of molecules with higher p*K*_a_, such as phenols, amines, thiols, sulfonamides or amides (Scheme 1b). Since the variation in aromaticity of the self-immolative adaptor is expected to influence the disassembly kinetics,^10^ we hypothesised that weakly aromatic five-membered heterocycles might be useful as scaffolds for the release of low acidity compounds.^14^ Nitrofuran, nitrothiophene, nitropyrrole and nitroimidazole spacers have already been used as alternatives to the standard PABA derivatives because of their higher potential compared to nitrobenzyl spacers. So far, these spacers have been employed for the release of phosphoramidates,^15^ amines from carbamates,^16^ phenols (as resofurin, p*K*_a_ 6.6)^17^ or tertiary amines from the corresponding quaternary ammonium salts.^18^ Applications to depolymerisation or mechanically triggered release of functionally diverse payloads have been also described.^19^

## Results and discussion

We began our investigation by exploring the potential release of thiols from heteroaromatic scaffolds. Thiols are a special class of molecules that play an important role in biological systems.^20^ As excellent electron donors, thiols readily bind metals in proteins and act as potent metallo-enzyme inhibitors. In addition, due to their redox potential, thiols play an important role in controlling redox homeostasis and in scavenging ROS and RNS (reactive oxygen and/or nitrogen species).^21^ Because of this broad spectrum of activities, thiols are potentially useful agents in therapy that are often non-selective due to the diversity of targets.^22^ There are very few examples of pro-drug delivery of thiols, apart from thiol release by the reversible disulphide bond.^23^ With a p*K*_a_ of about 10–11, thiols should not be released from simple PABA-like systems, while binding *via* a thiocarbonate or a thiocarbamate is not suitable due to the instability of these functional groups in plasma.^24^

**Scheme 2:**
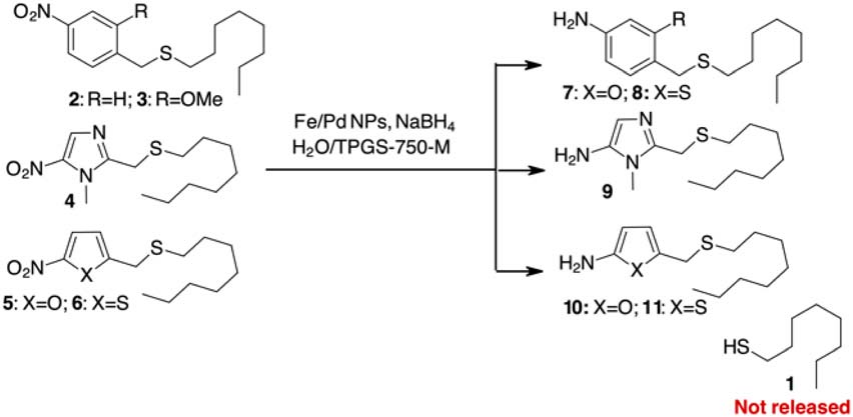
Attempts to use PABA or heterocyclic linkers for self-immolative release of a thiol.

To test this hypothesis, we decided to prepare different sulphides from 1-octanethiol (1) with various benzylic or heterocyclic scaffolds (compounds 2–6 in Scheme 2) to test the release of the thiol after reduction of the nitro group. The reduction of the nitroaromatic to release the payload was previously described with Zn in AcOH,^25^ but these conditions gave poor results in our hands, probably due to poor compatibility of the reagents with sulphur-containing molecules. We decided to investigate the possibility of using a mild procedure based on NaBH_4_ in the presence of Fe/Pd nanoparticles (NPs) in an aqueous/micellar solution (2 wt% TPGS-750-M) at rt.^26^ These conditions appear to be comparable with enzymatic processes that occur in aqueous medium. When applied to the PABA-like sulphides 2–3, reduction of the nitro group occurred within 2 hours in almost quantitative yield, but stable amino derivatives 7–8 were formed (Scheme 2). The presence of free 1-octanethiol 1 was never observed in tlc, ESI/MS and GC/MS analysis. Similar behaviour was observed when 1 was linked to (1-methyl-5-nitroimidazol-2-yl)methanol or 5-nitrofuryl- and 5-nitrothienyl-methanol (sulphides 4–6 in Scheme 2). Reduction of the nitro group gave the corresponding heteroaromatic amines 9–11 without liberation of the free thiol.

**Scheme 3:**
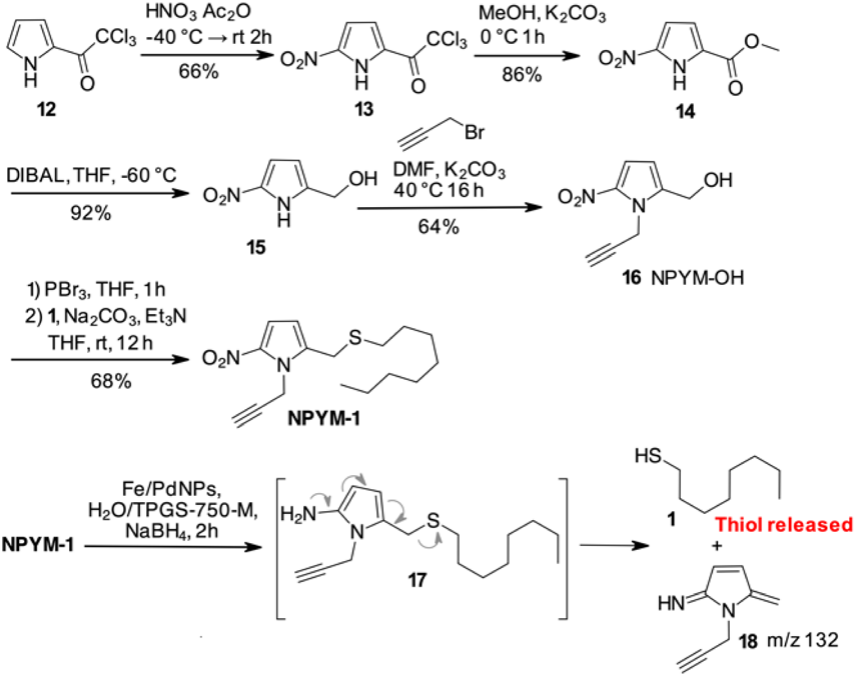
Synthetic scheme for NPYM-OH 16 and reductive release of 1-octanethiol.

Despite these disappointing results, we decided to investigate a 5-nitro-pyrrol-2-yl-methanol derivative (Scheme 3).

Starting from commercially available 2-(trichloroacetyl)pyrrole 12, an efficient 4-step synthesis of NPYM-OH was developed. Nitration of 12 was carried out with nitric acid in acetic anhydride to give the 5-nitro-trichloroacetylpyrrole 13, which was then converted to the corresponding methyl ester 14. Reduction of the ester to the corresponding alcohol 15 took place in the presence of DIBAL in THF at low temperature. Alkylation of the nitrogen with propargyl bromide was carried out with Na_2_CO_3_ in DMF at 40 °C to give product 16 in acceptable yield. The introduction of the alkyne in position 1 of the pyrrole ring was convenient to remove the reactive pyrrole NH and provided an additional appendage for further functionalisation by click chemistry. The final product 16 was isolated in four steps in 33% yield and the process could be applied to the preparation of 16 on a gram scale. Starting with 16, conversion to the corresponding bromide with PBr_3_, immediately followed by the introduction of 1-octanethiol 1, gave the model compound NPYM-1 in 68% yield (Scheme 3). When NPYM-1 was subjected to Fe/Pd nanoparticle-mediated reduction of the nitro group, we were pleased to observe the formation of free thiol 1 in solution, with complete conversion achieved in nearly 2 hours after addition of the reducing agent. Careful inspection of the HPLC/MS reaction mixture revealed a peak at *m*/*z* 133 corresponding to the protonated form of 5-methylene-3-pyrroline-2-imine 18 (Scheme 3), which is not stable enough for isolation.^27^ The presence of this product confirms the proposed mechanism for the release of the thiol by a 1,6 elimination through the undetected 2-amino derivative 17 (Scheme 3).

**Scheme 4:**
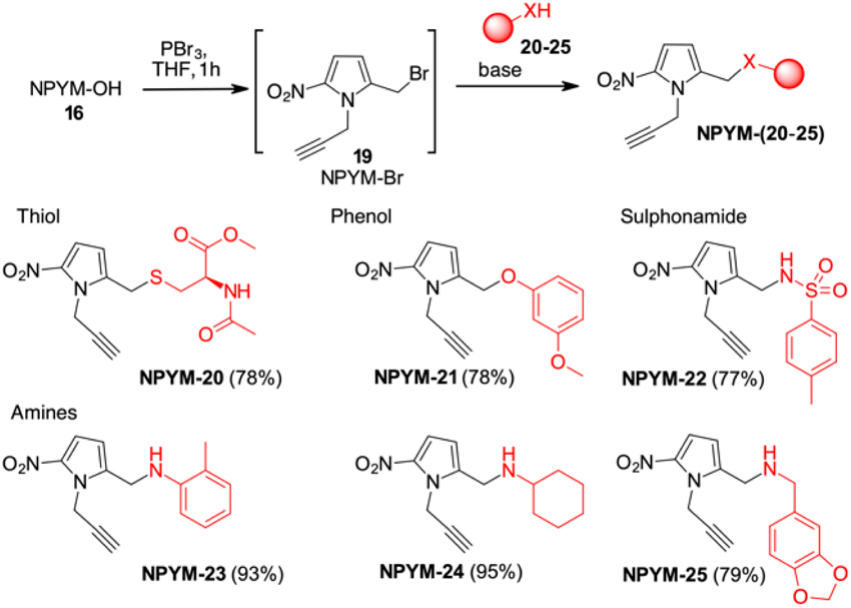
Preparation of NPYM derivatives of thiols, sulfonamides, and amines.

The potential of this new bioreductive donor was explored with other nucleophilic functional groups to verify the scope of this system. The general approach to introduce the NPYM moiety was the reaction with the bromide NPYM-Br 19 formed *in situ* from NPYM-OH and PBr_3_ (Scheme 4). *N*-Acetylcysteine 20, *m*-methoxyphenol 21, *p*-toluensulfonamide 22 and aliphatic or aromatic amines (23–25) reacted rapidly with NPYM-Br to give the compounds NPYM-(20–25). Depending on the nucleophile, different reaction conditions were required for the introduction of the NPYM framework. The optimised procedures (see the ESI†) gave products NPYM-(20–25) in good yields (Scheme 4).

This group of compounds is representative of common functional groups in drugs where conjugation for stimulus-driven release poses some problems. It is known that phenols are only released from PABA-like self-immolative spacers when their p*K*_a_ is below 9. Sulfonamides are an important class of molecules active as antiviral compounds, diuretics, non-steroidal anti-inflammatory drugs, cardiovascular drugs and many others. The p*K*_a_ value of sulfonamides, which is between 9 and 10, prevents their self-immolative release from molecular adaptors based on aza-quinone methide chemistry (*p*-toluenesulfonamide 22, p*K*_a_ ≂ 10.2). Consequently, the development of sulfonamide-modified prodrugs has been limited to molecules that can only temporarily improve the physicochemical properties by converting the sulphonamide into the corresponding *N*-acyloxyalkyl,^28^*N*-acyl, *N*-phthalyl^29^ or *N*-phosphoramidic acid derivatives.^30^ However, all these types of functional groups lead to products that are not particularly stable in physiological fluids and are not suitable for targeted or stimuli-sensitive delivery. Recently, the release of resatorvid (TAK-242), a drug containing a sulphonamide group with p*K*_a_ 8.0–8.1 was reported to occur using a PABA-like linker.^31^ Finally, aromatic or aliphatic amines have very low nucleofugacity and release by a self-immolative process occurs only after conversion to the corresponding carbamates. The only exception is the release of tertiary amines from the corresponding quaternary ammonium salt obtained by alkylation with a PABA-Cl derivative.^32^

**Fig. 1:**
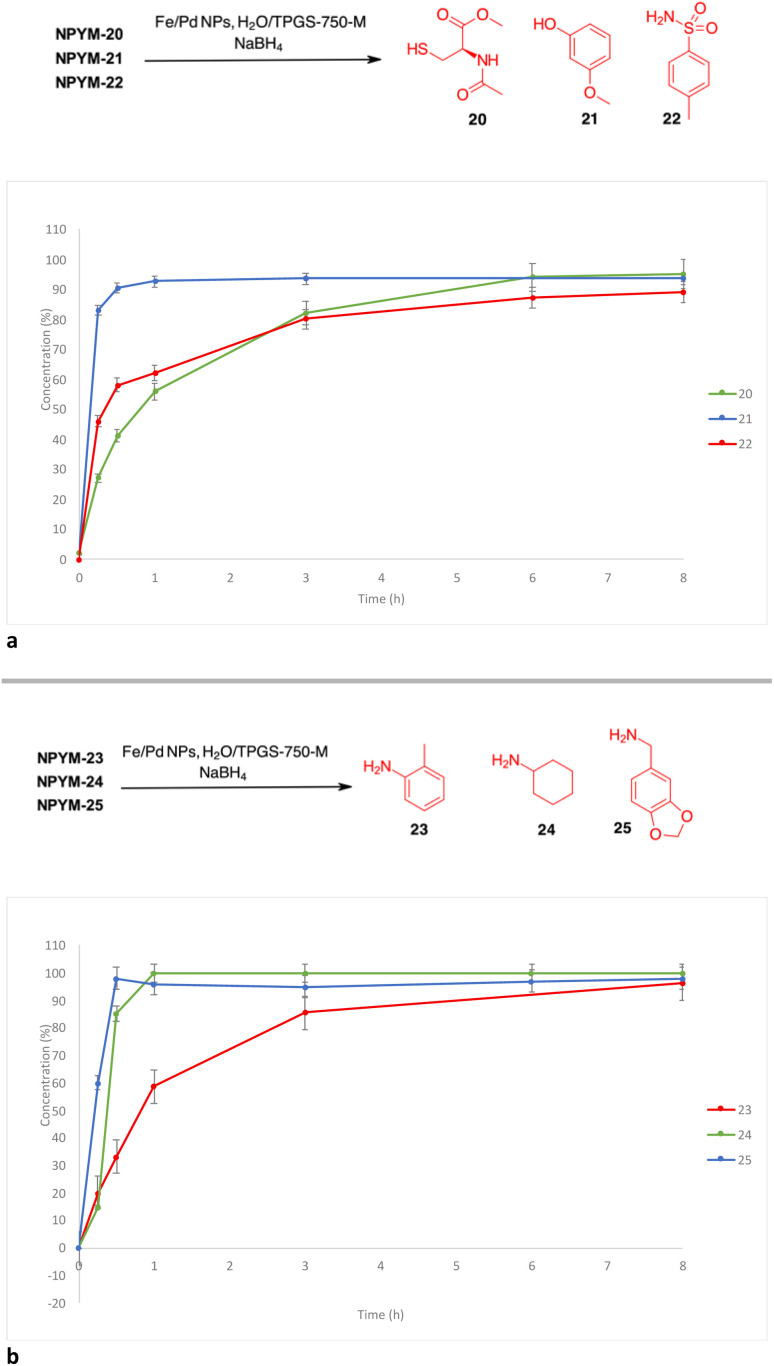
(a) Release profiles of compounds 20–22 from the corresponding NPYM adducts. (b) Release profiles of compounds 23–25 from the corresponding NPYM adducts.

Reductive release of compounds NPYM-(20–25) was performed with Fe/Pd NPs and NaBH_4_ in micellar environment H_2_O/TPGS-750-M (2%) at room temperature. The concentrations of the starting materials and the released products were determined *via* HPLC-MS at the following time intervals: 0.0 h (*t*_0_), 0.25 h (*t*_1_), 0.5 h (*t*_2_), 1 h (*t*_3_), 3 h (*t*_4_), 8 h (*t*_5_). The release profiles are shown in Fig. 1. The release of thiol 20 and sulfonamide 22 started rapidly and continued more slowly until it reached an almost complete release after 8 h (*t*_1/2_ < 1 h). Phenol 21 was released more rapidly and reached the plateau corresponding to complete release after 30 minutes (Fig. 1a). Surprisingly, even poor leaving groups like amines 23–25 were released after the reduction of the nitro group (Fig. 1b). Aliphatic amines 24 and 25 reached the saturation plateau after 1 h, while *o*-toluidine required 8 h to reach 90% of the released product, even if the *t*_1/2_ was about 1 h.

**Scheme 5:**
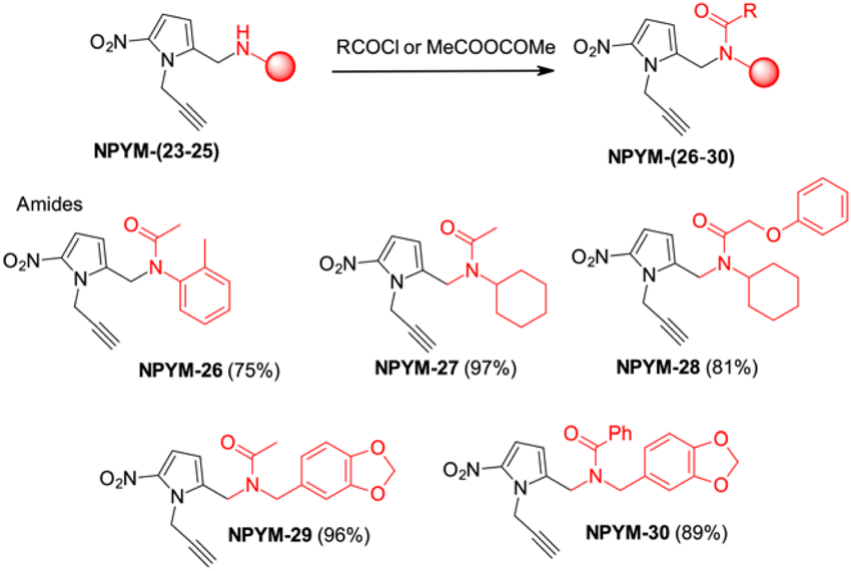
Preparation of NPYM derivatives of amides.

The above observations prompted us to investigate the behaviour of the NPYM scaffold linked to (carboxy)amides. Amides are ubiquitous in nature and include many important biological compounds and drugs.^33^ Despite their importance, there are very few bioreversible prodrugs or self-immolative linkers for bioconjugation and traceless release of amides.^34^ They are poor nucleophiles, and the low acidity of the amide hydrogen (p*K*_a_ > 14) prevents nucleofugacity from carriers and donor systems. To introduce the NPYM linker, we adopt an indirect approach by first binding the NPYM to primary amines 23–25 and then acylating the secondary amine formed. Reaction of NPYM-(23–25) with acetic anhydride, benzoyl chloride and phenoxyacetyl chloride, respectively, gave the products NPYM-(26–30) in good yields (Scheme 5).

**Fig. 2:**
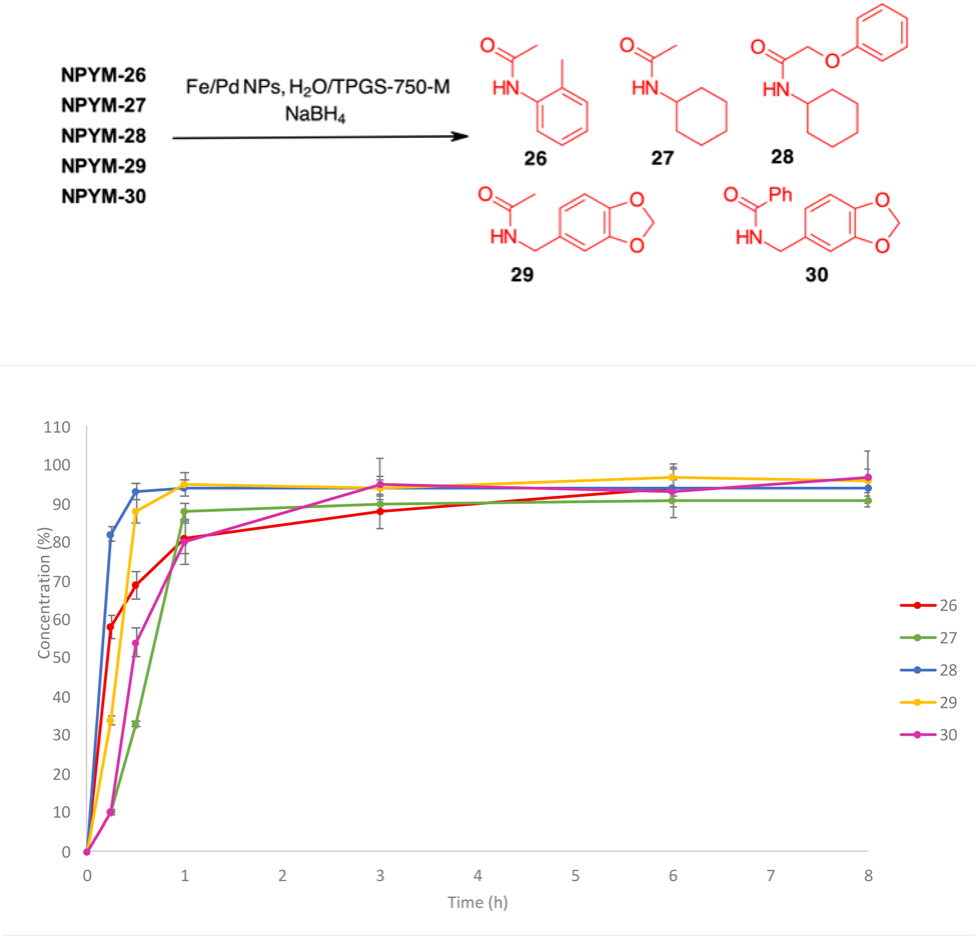
Release profiles of amides 26–30 from the corresponding NPYM adducts.

The NPYM scaffold proved to be a good donor system for amides as well. After treatment with Fe/Pd nanoparticles in a micellar environment, all amides 26–30 were released within 2 h (Fig. 2).

To understand the reason for the remarkable reactivity of our NPYM scaffold to release poor leaving groups directly bound to the linker, we investigated the mechanism by quantum chemical calculations, comparing the activation free energies (Δ*G*^‡^) of our linker and the other 5-membered heterocycles.^35^ We performed quantum chemical calculations using Gaussian16 software, adopting the B3LYP functional including Grimme's D3 dispersion with Becke–Johnson damping (D3BJ)^36^ in combination with the triple ξ def2-TZVPP basis set and the implicit polarisable continuum mode,^37^ in its integral equation formalism (IEF-PCM) to include solvation effects for water.

**Scheme 6:**
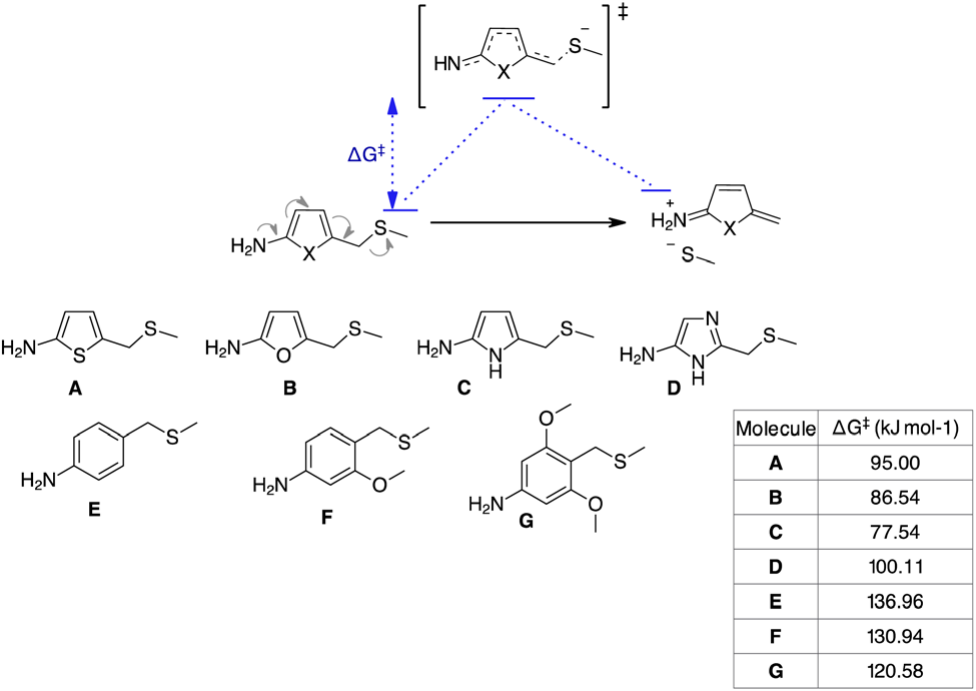
Model reaction and substrates subject to quantum-chemical calculations.

We identified transition states (TSs) by first performing a relaxed potential energy surface (PES) scan, increasing the distance between the benzyl C atom and the leaving group heteroatom in steps of 0.075 Å steps. Then, starting from the maximum of the PES scan, we began TS optimisation, freezing the distance between the benzyl C atom and the leaving group heteroatom. When we obtained a second order saddle point, we removed the second imaginary mode, shifted the geometry along this vibrational coordinate and optimised again. Following this procedure, we identified TSs for all the transformations of structures A–G in Scheme 6. As expected, they are described by a single imaginary mode that shifts the atoms along the reaction coordinate (see the ESI†). Thermodynamic corrections to the electronic energies can be estimated from the Hessians of the reactants and TSs. The free activation energies (Δ*G*^‡^, see the table in Scheme 6) are in agreement with our experimental trend regarding the release halftimes, as the 5-aminopyrrole frame shows the lowest Δ*G*^‡^ value compared to the other 3 heterocycles. Furthermore, the Δ*G*^‡^ values obtained are consistent with our observation that PABA-like linkers do not release thiols. An indirect validation of the method was applied to a number of PABA-like derivatives for which half-times of phenol release have been described in the literature.^13*a*^ Our estimated Δ*G*^‡^ values are in qualitative agreement with the release trend experimentally observed (see the ESI†) and also show that the release of phenols is easier (lower Δ*G*^‡^) than that of (aliphatic) thiols, despite their comparable p*K*_a_.

**Scheme 7:**
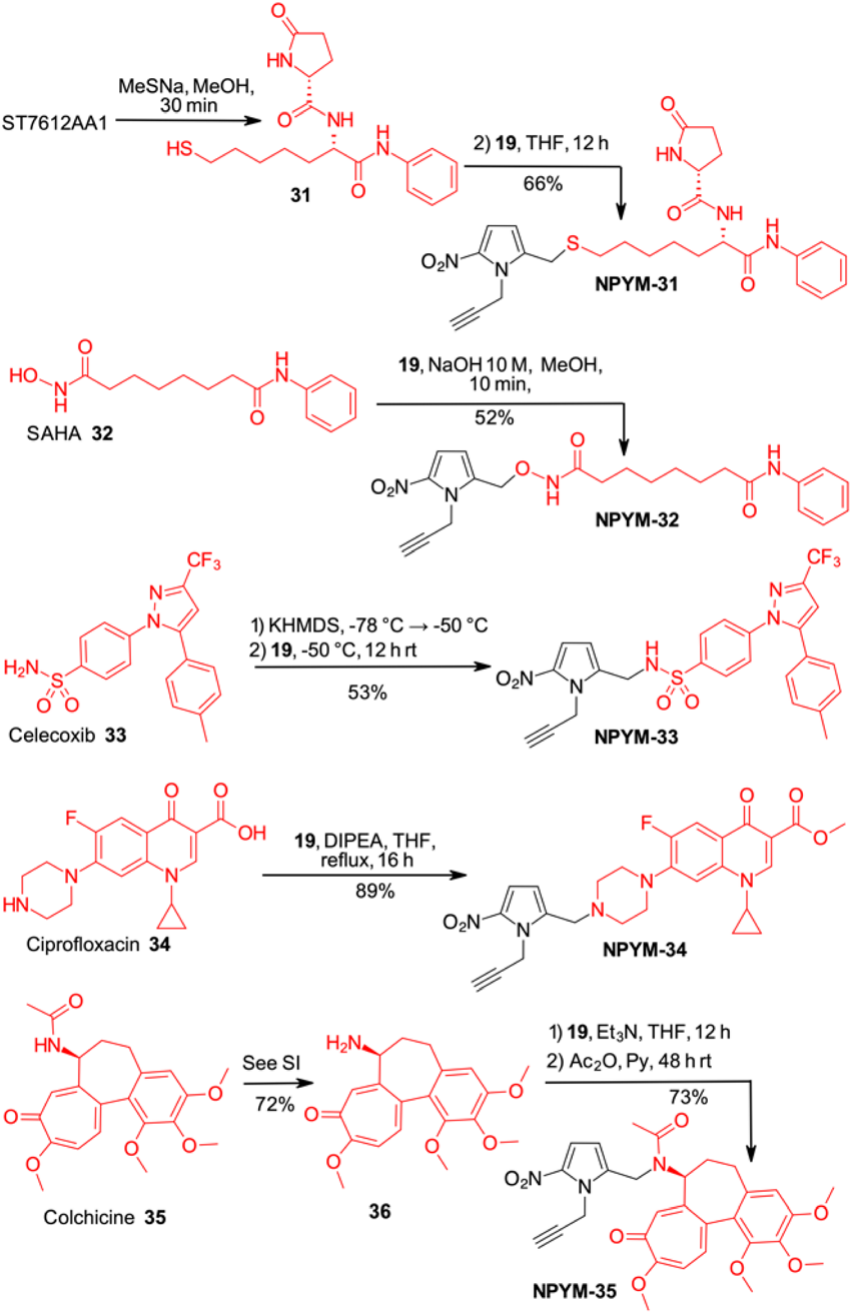
Late-stage introduction on the NPYM frame on different drugs.

**Scheme 8:**
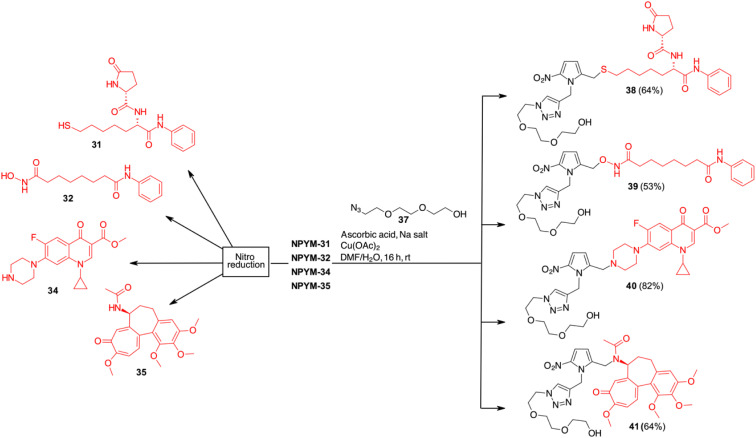
Click chemistry and release of drugs from NPYM derivatives.

Finally, the scope of the catch and (traceless) release system based on the NPYM linker has been used to modify thiol 31, the active species of the HDAC inhibitor ST7612AA1, vorinostat 32, celecoxib 33, ciprofloxacin 34 and colchicine 35 for pro-drug applications (Schemes 7 and 8). ST7612AA1 is a potent HDAC pan inhibitor, exhibiting high affinity for HDAC isoforms 1–3, 6, 8, 10, 11 and *in vitro* activity in the nanomolar range (IC_50_ = 50 nM on NCI-H460 cells) combined with remarkable *in vivo* antitumour activity.^38^ The thioester releases in a few minutes thiol 31, which is the potent HDAC inhibitor, as the –SH acts as a strong Zn-binding group.^38*c*^ Vorinostat, the first approved HDACi drug, is a hydroxamic acid, and several examples of prodrugs based on standard 1,6-self immolative systems have been reported as the free hydroxamic acid has some pharmacokinetic problems.^39^*p*-Nitrobenzyl alcohol and nitroimidazolyl derivatives of vorinostat have been described with varying results in terms of yield and activity.^40^ Celecoxib is a non-steroidal anti-inflammatory drug (NSAID) that acts as a COX-2 inhibitor.^41^ In addition to its typical use as an NSAID for the treatment of pain and inflammation, it has recently been investigated as a potential inhibitor of PEG2 synthesis for the treatment of malignancies.^42^ Since it contains an arylsulfonamide with a p*K*_a_ around 11, it was also a perfect model compound for our study. Ciprofloxacin (as a methyl ester), a fluoroquinolone broad-spectrum antibiotic containing a secondary amine, was selected for conjugation. Although several derivatives were prepared to access prodrugs based on PABA-like carbamates,^43^ no examples of direct association with the amine were reported in the literature.

Finally, colchicine, one of the best-known molecules of plant origin, was selected as an example of a biologically active amide.^44^ It has been extensively studied for various therapeutic applications and several synthetic derivatives have been developed and used in pharmacological studies. With an amidic NH group as the only functional group suitable for a catch and traceless release approach, it was another good model to test our system.

While in the more nucleophilic compounds 31–34 the introduction of NPYM was based on a nucleophilic substitution on the bromide NPYM-Br, in the case of colchicine we had to hydrolyse the amide, functionalise the free amine with NPYM-Br and finally reintroduce the acetyl group (see Scheme 7).^45^ Treatment of NPYM-31, NPYM-32, NPYM-34 and NPYM-35 with NaBH_4_ over Fe/Pd nanoparticles in an aqueous micellar environment resulted in rapid release of the free drugs within 2 h, in perfect agreement with the behaviour of the model systems (Scheme 8). Moreover, to verify the introduction of an addendum frame in position 1 of the NPYM scaffold, NPYM-31, NPYM-32, NPYM-34 and NPYM-35 were subjected to copper-catalysed azide–alkyne cycloaddition (CuAACC) with azide 37 to afford compounds 38–41 in good to acceptable yields (Scheme 8). Products 38, 39 and 41 were found to be stable in water and PBS for 24 hours (see Table 1).

**Table 1:** Stability tests of compounds 38, 39 and 41

Sample	H_2_O^a^, *t*_1/2_^b^	pH 7.4^a^, *t*_1/2_^b^	Plasma^a^, *t*_1/2_^b^
38	>48 h	>48 h	33 h
39	>48 h	>48 h	24 h
41	>48 h	>48 h	>48 h

a Value expressed as percentage of the unmodified compound after 36 h (water and PBS solution) or 24 h (plasma) of incubation.

b Half-life (*t*_1/2_) expressed as the amount of time it takes before half of the drug is degraded.

Stability in biological fluids was also demonstrated by incubating them at a fixed concentration in the presence of human plasma at various time points (from 0 to 1440 minutes). All compounds showed a high percentage of plasma stability up to 8 h after incubation, with the unmodified compounds decreasing slightly after 24 hours. Amide derivative 41 proved to be the most stable of the series with a half-life (*t*_1/2_) of more than 48 hours, while the stability of hydroxamic acid ester 39 and sulphide 38 decreased slightly, probably due to the hydrolytic action of plasma esterase or metabolic oxidation. For all these products, however, the half-life was more than 24 h.

Finally, to confirm the potential of NPYM for use as a prodrug, compound 38 was tested for its cytotoxicity in tumour cell lines. The presence of a nitro group on NPYM qualifies the linker as suitable for the preparation of hypoxia-activated prodrugs (HAPs).^46^ These molecules are inactive in normoxic cells, but after activation by endogenous oxidoreductases, which are highly expressed under hypoxic conditions, they release the drug only in the hypoxic environment. Traditionally, highly cytotoxic agents have been used in HAPs (*e.g.* DNA-damaging or tubulin aggregation inhibitors), with variable success due to overlapping toxicities.^47^ During hypoxia, there are significant changes in histone modification, *e.g.* overactivation of HDACs and changes in HDAC-protein interactions.

Inhibition of HDACs under hypoxic conditions leads to a reduction in HIF1a expression and activity, *via* a mechanism that is not well understood.^19*a*^

**Scheme 9:**
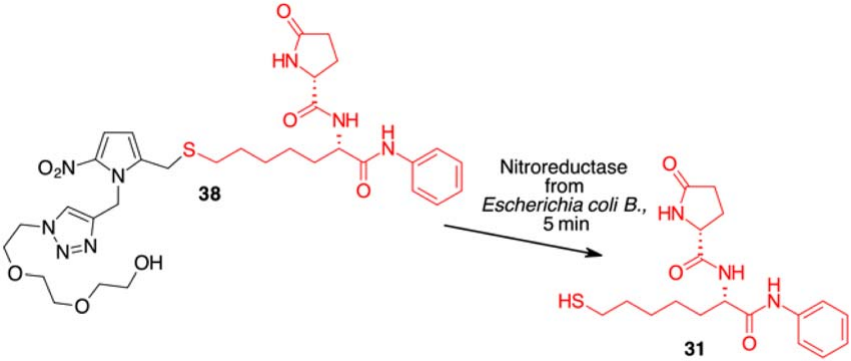
Enzyme mediated release of 31 from NPYM derivative 38.

**Fig. 3:**
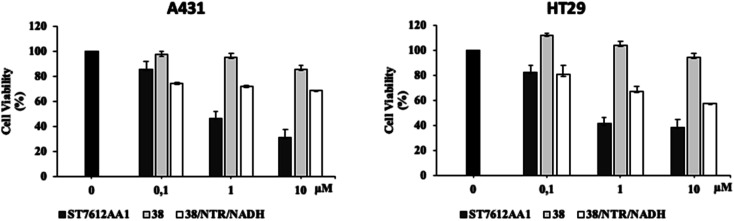
Effect of 38 in A431 and HT29 cells under normoxic conditions and activation by exogenous NTR and NADH. Cell viability of A431 and HT29 cancer cells treated with increasing concentrations of 38 alone and in the presence of exogenous NTR NADH assessed by MTT assays. These results are representative of three independent experiments.

**Fig. 4:**
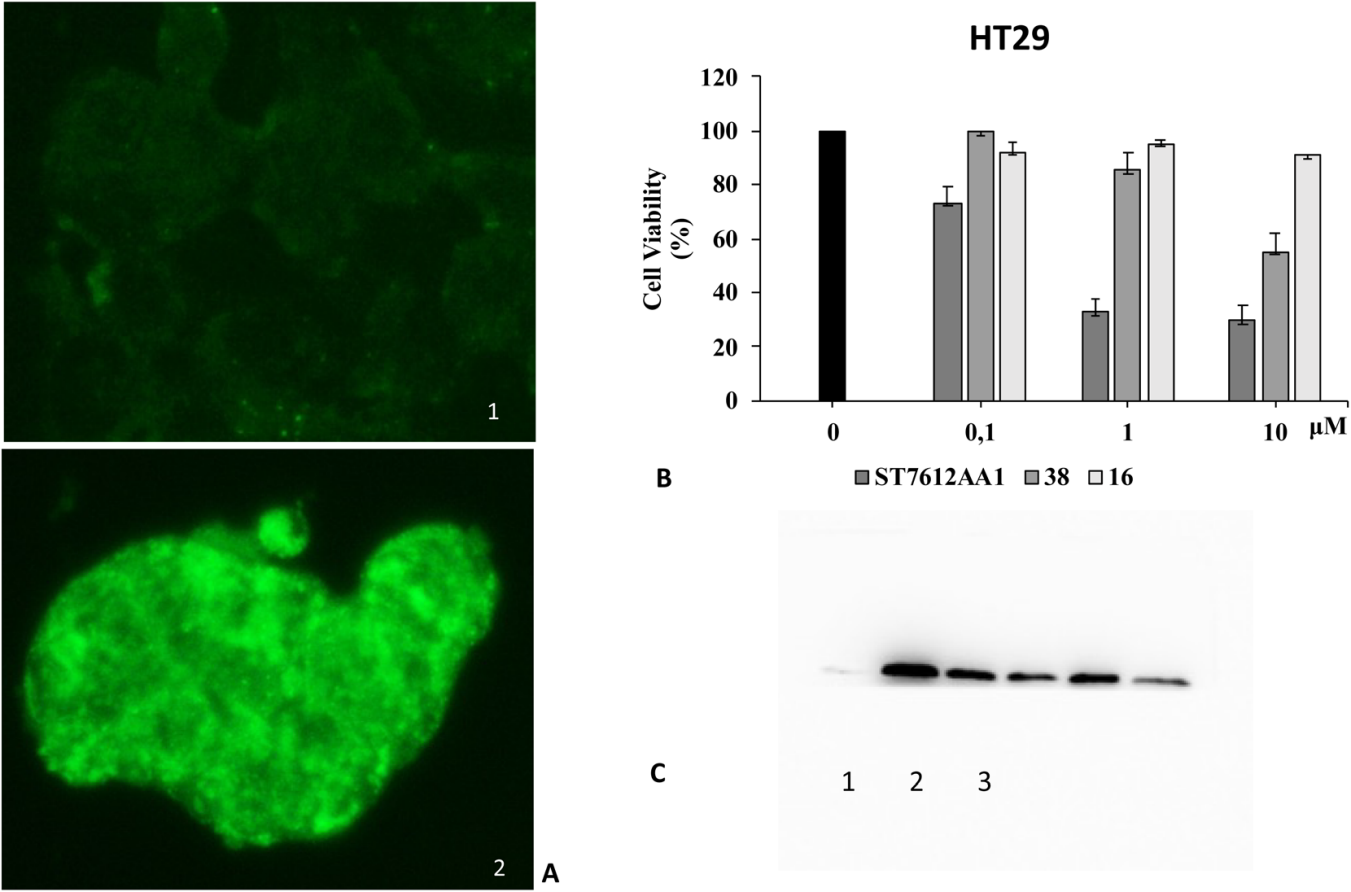
Effect of compound 38 activated by endogenous nitroreductase in HT29 cells. (A) Assessment of nitroreductase activation with the Image-iTTM green hypoxia reagent. (1) Fluorescence images of nitroreductase (40× magnification) in HT29 cells under normoxic conditions. (2) Fluorescence images of nitroreductase (40× magnification) in HT29 cells under hypoxic conditions. (B) Cell viability of HT29 cancer cells treated with increasing concentrations of 38 compared to the original ST7612AA1 drug and pyrrolyl methanol 16 under hypoxic conditions as measured by MTT assays. These results are representative of three independent experiments. (C) Western blot analysis of acetyl histone H4 levels in HT29 cells treated for 48 hours under hypoxic conditions: (1) control, (2) ST7612AA1, (3) compound 38. In the original gel the other three spots on the left are HDAC inhibitors not related to this work. Beta-actin was used for normalisation (see the ESI†).

Since HDAC inhibitors are also effective radiosensitizers and hypoxia interferes with radiotherapy, a HAP, based on an HDAC inhibitor, could be useful in the case of radiotherapy resistance due to tumour hypoxia.^47^ Thus, the availability of HAPs that selectively inhibit HDAC activity in hypoxia would allow alteration of the epigenetic profile in tumours with a favourable clinical outcome.^40*b*^ First, we confirm the release of thiols 31 from 38 using oxygen-insensitive nitroreductase (NTR) from *Escherichia coli* B. This enzyme is the most commonly used reductase for antibody- and gene-directed enzyme prodrug therapy strategies (ADEPT and GDEPT) and it is the reference enzyme for nitro group-containing prodrugs.^48^ With NTR, substrates are reduced in a concerted two-electron reduction, bypassing the oxygen-sensitive prodrug radical, which can be reoxidised by oxygen to the original nitro compound. Activation of 38 by NTR reduction was studied by incubation in aqueous solutions containing the enzyme (2 μg mL^−1^) and NADH (1 mM) at 37 °C (Scheme 9). Compound 38 was rapidly reduced, releasing more than 90% of thiol 31 in the solution in 5 min. To further characterise our NPYM adduct, the metabolic stability of compound 38 was investigated in the presence of human liver microsomes to evaluate a possible interaction of the pyrrole ring contained in NPYM with cytochrome P450. We were pleased to find that compound 38 exhibited good metabolic stability (93%, see the ESI†). The only observed (mild) phase I metabolism resulted in the formation of a monooxidate derivative (M1 = M + 16), which was probably formed by oxidation of the sulphide to sulphoxide without involvement of the pyrrole ring. The metabolite was detected and quantified with HPLC-UV-MS. Then A431 epidermoid carcinoma cells and HT29 colorectal adenocarcinoma cells were selected for an exploratory study (Fig. 3). The latter are considered a standard substrate for hypoxic conditions as they produce dl-diaphorase, an obligate two-electron reductase that bioactivates nitroaromatics.^49^ Cells were incubated in the presence of the test compounds at various concentrations for 72 h under normoxic or hypoxic conditions, and cell viability and proliferation behaviour were assessed by MTT. First, the activity of 38 was examined under normoxic conditions compared to the parent compound ST7612AA1. As shown in Fig. 3, ST7612AA1 drastically reduced cellular viability to 40% already at a concentration of 1 μM, while prodrug 38 did not show the same toxicity as the reference compound but showed remarkable stability and low toxicity in tumour cell culture (cell viability 90–95% even at 10 μM). The release of the drug was induced by the addition of NTR and NADH to the cells, and some effect was observed (10 μM), with a more marked decrease in cell viability in HT29 cells, indicating that the drug was effectively released under bioreductive conditions (Fig. 3). Due to the increased drug sensitivity of HT29 cells, we decided to perform hypoxia experiments only with this type of cancer cell. HT29 cells were then treated under hypoxic conditions (94% N_2_, 5% CO_2_, 1% O_2_) and the extent of nitroreductase activation was assessed using the Image-iT green hypoxia reagent.^50^ The fluorescence images in Fig. 4A showed that the target enzyme was highly expressed in the HT29 culture. Therefore, the cells were treated with compounds ST7612AA1, 38 and also with NPYM-OH alone, and only ST7612AA1 and 38 showed comparable activity at 10 μM (Fig. 4B). Comparing the data on 38 with the same cell line under normoxic conditions, we observed a decrease in cell viability from 95 to 55% after 48 hours, confirming good selectivity under hypoxia and demonstrating the effective reduction of the NPYM framework in the cell.

Moreover, both 38 and NPYM-OH were moderately toxic in cancer cells or in a human fibroblast cell line, at least up to a concentration of 10 μM. Finally, to demonstrate that the cytotoxic activity of 38 is due to effective HDAC inhibition, the release of 31 in HT29 cells was confirmed by western blot analysis of the total protein lysate (Fig. 4C). Comparison of the band intensity clearly shows an increase in acetylation of HDAC-4 when cells are treated with ST7612AA1 and 38 compared to cells treated with vehicle alone.

## Conclusions

In summary, we have shown that 5-nitropyrrolylmethanol (NPYM-OH) is a valid alternative to the standard PABA-like system for the release of molecules containing poor leaving groups such as thiols, amines, amides, hydroxamic acids, sulfonamides or carboxamides after reduction of the nitro group. The release occurred with NaBH_4_ and Pd nanoparticles in aqueous micellar medium using TPGS-750-M 2% and under NADH-dependent nitroreductase activation. The NPYM-OH scaffold was used as a late-stage functionalisation of ST7612AA1, a thiol-based HDAC inhibitor, and of approved drugs such as vorinostat (SAHA), ciprofloxacin, celecoxib and colchicine, which contain a hydroxamic acid, a secondary amine, a primary sulfonamide and a secondary carboxamide, respectively, as the only anchor point. This peculiar reactivity was explained by a lower free energy of the TS formed during 1,6-elimination of 5-aminopyrrole. The NPYM-OH scaffold was finally used to prepare a hypoxia-activated prodrug based on ST7612AA1, which showed good selectivity as it is much less toxic than the corresponding drug and has reasonable cytotoxicity in hypoxia-sensitive cell cultures due to HDAC inhibition. The NPYM-OH alone also shows low toxicity in cancer cells and in human fibroblasts, making it suitable for the preparation of various reduction-sensitive materials containing the functional groups mentioned above. Further bioconjugation of this new scaffold with a targeted component is under investigation and will be reported in due course.

## Data availability

All data were inserted in the ESI.†

## Author contributions

E. E., F. F. and M. T. designed the project. A. B. and F. P. collected chromatographic data and performed ADME experiments, L. P. carried out cell experiments, E. E and P. T. carried out synthetic procedures, D. P. carried out the computational analysis, and E. C., G. G., F. F. and L. T. drafted the manuscript. All authors were involved in the data analysis, wrote the paper, and approved the final version of this manuscript.

## Conflicts of interest

There are no conflicts to declare.

## Supplementary Material

SC-015-D4SC01576B-s001

SC-015-D4SC01576B-s002

## References

[cit1] Trovato V., Sfameni S., Rando G., Rosace G., Libertino S., Ferri A., Plutino M. R. (2022). Molecules.

[cit2] Wang S., Urban M. W. (2023). Chem.

[cit3] Zhao J.-L., Li M.-H., Cheng Y.-M., Zhao X.-W., Xu Y., Cao Z.-Y., You M.-H., Lin M.-J. (2023). Coord. Chem. Rev..

[cit4] Dong J., O'Hagan M. P., Willner I. (2022). Chem. Soc. Rev..

[cit5] Ma X., Li S.-J., Liu Y., Zhang T., Xue P., Kang Y., Sun Z.-J., Xu Z. (2022). Chem. Soc. Rev..

[cit6] Taresco V., Alexander C., Singh N., Pearce A. K. (2018). Adv. Ther..

[cit7] Gnaim S., Shabat D. (2014). Acc. Chem. Res..

[cit8] Gonzaga R. V., do Nascimento L. A., Santos S. S., Machado Sanches B. A., Giarolla J., Ferreira E. I. (2020). J. Pharm. Sci..

[cit9] Alouane A., Labruére R., Le Saux T., Schmidt F., Jullien L. (2015). Angew. Chem., Int. Ed..

[cit10] Robbins J. S., Schmid K. M., Phillips S. T. (2013). J. Org. Chem..

[cit11] Dal Corso A., Arosio S., Arrighetti N., Perego P., Belvisi L., Pignataro L., Gennari C. (2021). Chem. Commun..

[cit12] Roberts D. A., Pilgrim B. S., Dell T. N., Stevens M. M. (2020). Chem. Sci..

[cit13] Dunsmore L., Navo C. D., Becher J., de Montes E. G., Guerreiro A., Hoyt E., Brown L., Zelenay V., Mikutis S., Cooper J., Barbieri I., Lawrinowitz S., Siouve E., Martin E., Ruivo P. R., Rodrigues T., da Cruz F. P., Werz O., Vassiliou G., Ravn P., Jiménez-Osés G., Bernardes G. J. L. (2022). Nat. Chem..

[cit14] Schmid K. M., Jensen L., Phillips S. T. (2012). J. Org. Chem..

[cit15] Borch R. F., Liu J., Schmidt J. P., Marakovits J. T., Joswig C., Gipp J. J., Mulcahy R. T. (2000). J. Med. Chem..

[cit16] Hay M. P., Anderson R. F., Ferry D. M., Wilson W. R., Denny W. A. (2003). J. Med. Chem..

[cit17] Chaplin D. J., Trawick M. L., Pinney K. G. (2017). Bioorg. Med. Chem. Lett..

[cit18] Tercel M., Lee A. E., Hogg A., Anderson R. F., Lee H. H., Siim B. G., Denny W. A., Wilson W. R. (2001). J. Med. Chem..

[cit19] Kim H., Brooks A. D., Dilauro A. M., Phillips S. T. (2020). J. Am. Chem. Soc..

[cit20] Chowdhury R., Candela-Lena J. I., Chan M. C., Greenald D. J., Yeoh K. K., Tian Y.-M., McDonough M. A., Tumber A., Rose N. R., Conejo-Garcia A., Demetriades M., Mathavan S., Kawamura A., Lee M. K., van Eeden F., Pugh C. W., Ratcliffe P. J., Schofield C. J. (2013). ACS Chem. Biol..

[cit21] Klingler F.-M., Wichelhaus T. A., Frank D., Cuesta-Bernal J., El-Delik J., Müller H. F., Sjuts H., Göttig S., Koenigs A., Pos K. M., Pogoryelov D., Proschak E. (2015). J. Med. Chem..

[cit22] Frost L., Suryadevara P., Cannell S. J., Groundwater P. W., Hambleton P. A., Anderson R. J. (2016). Eur. J. Med. Chem..

[cit23] Srinivas N. R., Mamidi R. N. V. S. (2003). Biomed. Chromatogr..

[cit24] Subramanian S., Bates S. E., Wright J. J., Espinoza-Delgado I., Piekarz R. L. (2010). Pharmaceuticals.

[cit25] Berry J. M., Watson C. Y., Whish W. J. D., Threadgill M. D. (1997). J. Chem. Soc., Perkin Trans. 1.

[cit26] Feng J., Handa S., Gallou F., Lipshutz B. H. (2016). Angew. Chem., Int. Ed..

[cit27] Balaban A. T., Oniciu D. C., Katritzky A. R. (2004). Chem. Rev..

[cit28] Thulam V. K., Kotte S. C. B., Sanjay Kumar H., Murali P. M., Mukkanti K., Mainker P. S. (2013). J. Pharma Res..

[cit29] Song R., Liu Y., Majhi P. K., Ng P. R., Hao L., Xu J., Tian W., Zhang L., Liu H., Zhang X., Zhang X., Chi Y. R. (2021). Org. Chem. Front..

[cit30] (a) Guarino V. and Stella V., in Prodrugs: Challenges and Rewards, Part 2, 2007, vol. V, p. 833

[cit31] Kostyo J. H., Lallande A. T., Sells C. A., Shuda M. R., Kane R. R. (2023). ACS Med. Chem. Lett..

[cit32] Staben L. R., Koenig S. G., Lehar S. M., Vandlen R., Zhang D., Chuh J., Yu S.-F., Ng C., Guo J., Liu Y., Flygare J. A., Pillow T. H. (2016). Nat. Chem..

[cit33] Amides 648 entries|DrugBank Online, https://go.drugbank.com/categories/DBCAT000494, accessed 30 October 2022

[cit34] Rannoux C., Roussi F., Martin M. T., Guéritte F. (2011). Org. Biomol. Chem..

[cit35] Rose D. A., Treacy J. W., Yang Z. J., Ko J. H., Houk K. N., Maynard H. D. (2022). J. Am. Chem. Soc..

[cit36] (a) Frisch M. J. , Trucks G. W., Schlegel H. B., Scuseria G. E., Robb M. A., Cheeseman J. R., Scalmani G., Barone V., Petersson G. A., Nakatsuji H., Li X., Caricato M., Marenich A. V., Bloino J., Janesko B. G., Gomperts R., Mennucci B., Hratchian H. P., Ortiz J. V., Izmaylov A. F., Sonnenberg J. L., Williams-Young D., Ding F., Lipparini F., Egidi F., Goings J., Peng B., Petrone A., Henderson T., Ranasinghe D., Zakrzewski V. G., Gao J., Rega N., Zheng G., Liang W., Hada M., Ehara M., Toyota K., Fukuda R., Hasegawa J., Ishida M., Nakajima T., Honda Y., Kitao O., Nakai H., Vreven T., Throssell K., Montgomery Jr J. A., Peralta J. E., Ogliaro F., Bearpark M. J., Heyd J. J., Brothers E. N., Kudin K. N., Staroverov V. N., Keith T. A., Kobayashi R., Normand J., Raghavachari K., Rendell A. P., Burant J. C., Iyengar S. S., Tomasi J., Cossi M., Millam J. M., Klene M., Adamo C., Cammi R., Ochterski J. W., Martin R. L., Morokuma K., Farkas O., Foresman J. B. and Fox D. J., Gaussian 16 Revision C.01, Gaussian Inc, Wallingford CT, 2016

[cit37] Tomasi J., Mennucci B., Cammi R. (2005). Chem. Rev..

[cit38] Giannini G., Vesci L., Battistuzzi G., Vignola D., Milazzo F. M., Guglielmi M. B., Barbarino M., Santaniello M., Fanto N., Mor M., Rivara S., Pala D., Taddei M., Pisano C., Cabri W. (2014). J. Med. Chem..

[cit39] Rubio-Ruiz B., Weiss J. T., Unciti-Broceta A. (2016). J. Med. Chem..

[cit40] Calder E. D. D., Skwarska A., Sneddon D., Folkes L. K., Mistry I. N., Conway S. J., Hammond E. M. (2020). Tetrahedron.

[cit41] Szabó G., Fischer J., Kis-Varga Á., Gyires K. (2008). J. Med. Chem..

[cit42] O'Callaghan G., Houston A. (2015). Br. J. Pharmacol..

[cit43] Ji C., Miller P. A., Miller M. J. (2015). ACS Med. Chem. Lett..

[cit44] Gracheva I. A., Shchegravina E. S., Schmalz H.-G., Beletskaya I. P., Fedorov A. Yu. (2020). J. Med. Chem..

[cit45] Ghawanmeh A. A., Al-Bajalan H. M., Mackeen M. M., Alali F. Q., Chong K. F. (2020). Eur. J. Med. Chem..

[cit46] Sharma A., Arambula J. F., Koo S., Kumar R., Singh H., Sessler J. L., Kim J. S. (2019). Chem. Soc. Rev..

[cit47] Spiegelberg L., Houben R., Niemans R., de Ruysscher D., Yaromina A., Theys J., Guise C. P., Smaill J. B., V Patterson A., Lambin P., Dubois L. J. (2019). Clin. Transl. Radiat. Oncol..

[cit48] Roldán M. D., Pérez-Reinado E., Castillo F., Moreno-Vivián C. (2008). FEMS Microbiol. Rev..

[cit49] Beyer R. E., Segura-Aguilar J., Di Bernardo S., Cavazzoni M., Fato R., Fiorentini D., Galli M. C., Setti M., Landi L., Lenaz G. (1996). Proc. Natl. Acad. Sci. U.S.A..

[cit50] Godet I., Doctorman S., Wu F., Gilkes D. M. (2022). Cells.

