# Supplementary material for: A novel bioresponsive self-immolative spacer based on aza-quinone methide reactivity for the controlled release of thiols, phenols, amines, sulfonamides or amides
Source: Chem Sci. 2024 Apr 2;15(16):6168–77. doi: 10.1039/d4sc01576b (PMC11041255; doi:10.1039/d4sc01576b)
Supplement: SC-015-D4SC01576B-s002 [file SC-015-D4SC01576B-s002.pdf]

Electronic Supplementary Material (ESI) for Chemical Science.
This journal is © The Royal Society of Chemistry 2024

A novel bioresponsive self-immolative spacer based on aza-quinone methide reactivity for the controlled release of thiols, phenols, amines, sulfonamides or amides

Elena Ermini,[a] Annalaura Brai,[a] Elena Cini, Federica Finetti,[a] Giuseppe Giannini,[b] Daniele Padula, Lucrezia Paradisi,[a] Federica Poggialini,[a] Lorenza Trabalzini,[a] Paola Tolu[a] and Maurizio Taddei*[a]

Supporting Information

General methods S1

Synthetic Procedures S2

HPLC-MS Analysis and release studies S18

Stability in water and PBS S23

Stability in Human Plasma S26

Metabolic Stability in human liver microsomes (HLM) S27

Cell culture, MTT assay and Western blotting. S31

Western blot gel original images S32

Indirect validation of the computational data S33

^{1}H and ^{13}C NMR of organic compounds S35

General methods

All reagents were used as purchased from commercial suppliers without further purification. Reactions were carried out in oven-dried or flame-dried vessels. Solvents were dried and purified by conventional methods before use or purchased in anhydrous form if available. Flash column chromatography was performed using Merck silica gel 60, 0.040-0.063 mm (230-400 mesh). Merck aluminium plates pre-coated with silica gel 60 (UV254) and visualised by staining with a $KMnO_4$ solution were used for analytical thin-layer chromatography. NMR spectra were recorded at 25 °C with a Bruker AC 400 or 600 MHz for ^1H and 100 or 150 MHz for ^{13}C respectively. Deuterated solvents were used as purchased. The solvent is indicated for each spectrum. Cleavage patterns are designated as s, singlet; d, doublet; t, triplet; q, quartet; m, multiplet; br, broad. Chemical shifts (δ) are given in part-per-million (ppm) relative to the resonance of the respective residual solvent peaks. ^{13}C are recorded with a JMODE experiment where C and CH_2 have a negative phase and CH and CH_3 a positive phase and reported only in case of new compounds. High- and low-resolution mass spectroscopy analyses were recorded by electrospray ionisation using a Q-exactive Plus mass spectrometer. HPLC/MS analyses were performed with the Agilent 1260 Infinity II Preparative LC /MSD System Single Quadrupole (LC /MSD iQ) connected to a UV detector (254 nm) using an InfinityLab Poroshell 120 EC -C18 column (2.1 x 50 mm, 2.7 µm), flow 0.6 mL/min, MeCN/H_2O gradient from 0.5:9.5 to 9.5:0.5 in 10 minutes. ESI ionisation, flow of drying gas (N2) 9 L/min, temperature 350 °C, atomising pressure 40 PSI, fragmentation.

Synthetic Procedures

Synthesis of thioethers, general procedure

The alcohol (1 mmol) was dissolved in dry THF (5 mL) and cooled to 0 °C. PBr_3 (2 mmol) was added to the solution and the reaction was allowed to warm up to room temperature for 1 h. The reaction mixture was then allowed to cool to room temperature. The crude reaction mixture was concentrated in vacuo and filtered through a silica gel path with EtOAc to afford the bromo-derivatives in quantitative yield as a light orange oil, identified by mass spectroscopy. The products were immediately used for the next steps. 1-octanethiol **1** (2.1 mmol) and K_2CO_3 (1.95 mmol) were added to dry THF (4 mL) in a round bottom flask and the suspension was stirred for 1 h under N_2 atmosphere at room temperature. The bromide derivative (1 mmol) and Et_3N (1.05 mmol) were added to the reaction and the mixture was left for 16 h under magnetic stirring. After complete consumption of the starting material, which was monitored by TLC, the reaction was taken up in EtOAc and washed with water and brine. The organic phase was dried over anhydrous Na_2SO_4, filtered and concentrated in vacuo. The crude reaction mixture was purified by silica gel flash chromatography to obtain the desired compound.

Nitro group reduction, general procedure

Iron-based nanoparticles[1] (6 mg) were placed in a round bottom flask. 0.5 mL aqueous solution of 2 wt% TPGS-750-M was added using a syringe and $NaBH_4$ (59.0 mg, 1.5 mmol) was added to the reaction mixture. The reaction flask was closed with a septum and the reaction mixture turned black with evolution of hydrogen gas. After 2 minutes, the nitro-containing compounds (0.5 mmol), pre-dissolved in a mixture of 0.5 mL aqueous 2 wt% TPGS-750-M and 0.1 mL THF, were added to the catalyst suspension and the reaction was stirred vigorously at room temperature. The reaction was monitored via HPLC-MS at 0.0 h (t0), 0.25 h (t1), 0.5 h (t2), 1 h (t3), 3 h (t4), 6 h (t5), 8 h (t6). The sample was prepared by taking 100 µL of the reaction mixture and extracting with EtOAc (200 µL x3). The combined organic layers were dried over anhydrous Na_2SO_4, filtered and concentrated in vacuo. The sample was then dissolved in 90 µL MeOH and 50 µL of this solution was removed and evaporated in vacuo. The final sample was diluted with 200 µL MeCN + 800 µL H_2O and filtered for injection. To isolate compounds **7-10**, the crude product from EtOAc evaporation was purified by silica gel flash chromatography.

Enzymatic nitro group reduction, general procedure

Recombinant E. coli nitroreductase and nicotinamide adenine dinucleotide (NADH, reduced form, dipotassium salt) were purchased from Sigma–Aldrich. Compound stock solution (15 □L, 250 □M) in DMSO was added to sodium phosphate buffer (410 □L, 10 mM, pH 7, preheated to 37 °C) and NADH (75 □L of 1mM) in sodium phosphate buffer. The reaction was initiated by addition of E. coli nitroreductase (1 mL, 2 □g mL^{-1} in sodium phosphate buffer). Aliquots were collected and the reactions were followed by HPLC-MS as a

function of time. The HPLC chromatogram were used to determine both the disappearance of the starting material as well as the appearance of the released product.

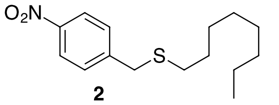

2

*1-[(p-Nitrophenyl)methylthio]octane (**2**).* Flash chromatography (PE:EtOAc/9:1) provided compound **2** (130 mg, 98 % yield). ^{1}H NMR (600 MHz, $CDCl_3$): δ 8.17 (d, J = 8 Hz, 2H), 7.48 (d, J = 8 Hz, 2H), 3.76 (s, 2H), 2.40 (t, J = 6.9 Hz, 2H), 1.59 – 1.48 (m, 2H), 1.37 – 1.12 (m, 12H), 0.87 (t, J = 6.9 Hz, 3H). ^{13}C NMR (150 MHz, $CDCl_3$): δ 146.66, 129.62, 123.74, 35.81, 31.79, 31.63, 29.14, 29.10, 28.80, 22.64, 14.10. MS (ESI): m/z calcd for $C_{15}H_{23}NO_2SNa$ $[M+Na]^+$: 304.1347; found: 304.1345.

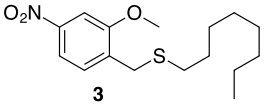

3

*1-[(2-Methoxy-4-nitrophenyl)methylthio]octane (**3**).* Flash chromatography (PE:EtOAc/9:1) provided compound **3** (24 mg, 52 % yield). ^{1}H NMR (600 MHz, $CDCl_3$): δ 7.82 (dd, J = 8.2, 2.0 Hz, 1H), 7.72 (d, J = 2.0 Hz, 1H), 7.41 (d, J = 8.3 Hz, 1H), 3.96 (s, 3H), 3.76 (s, 2H), 2.46 (m, 2H), 1.57 (m, 3H), 1.36 – 1.32 (m, 2H), 1.31 – 1.22 (m, 8H), 0.88 (t, J = 7.0 Hz, 3H). ^{13}C NMR (150 MHz, $CDCl_3$): δ 157.47, 148.04, 135.32, 130.18, 115.78, 105.54, 56.05, 32.10, 31.80, 30.11, 29.70, 29.32, 29.19, 29.17, 28.90, 22.64, 14.09. MS (ESI): m/z calcd for $C_{16}H_{25}NO_3SNa$ $[M+Na]^+$: 334.1453; found: 334.1452.

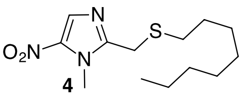

4

*5-Nitro-2-[(octylthio)methyl]imidazole (**4**).* Flash chromatography (PE:EtOAc/9:1) provided compound **4** (204 mg, 45 % yield). ^{1}H NMR (600 MHz, $CDCl_3$): δ 7.85 (s, 1H), 3.93 (s, 3H), 3.74 (s, 2H), 2.50 – 2.42 (m, 2H), 1.58 – 1.41 (m, 2H), 1.32 – 1.15 (m, 10H), 0.81 (t, J = 6.9 Hz, 3H). ^{13}C NMR (150 MHz, $CDCl_3$): δ 149.9, 139.4, 133.0, 131.9, 33.5, 31.7, 29.1, 29.0, 28.6, 27.8, 22.6, 14.0. MS (ESI): m/z calcd for $C_{13}H_{23}N_3O_2SNa$ $[M+Na]^+$: 308.1409; found: 308.1408.

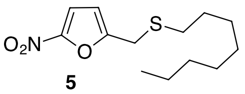

5

*2-Nitro-5-((octylthio)methyl)furan (**5**).* Flash chromatography (PE:EtOAc/95:5) in order to obtain the product **5** (114 mg, 30% yield). <u>^{1}H NMR</u> (600 MHz, $CDCl_3$): δ 7.26 (d, *J* = 3.6 Hz, 1H), 6.46 (d, *J* = 3.6 Hz, 1H), 3.74 (s, 2H), 2.57 (s, 2H), 1.61 – 1.55 (m, 2H), 1.40 – 1.33 (m, 2H), 1.28 (m, 8H), 0.87 (t, *J* = 7.0 Hz, 3H). <u>^{13}C NMR</u> (150 MHz, $CDCl_3$): δ 157.05, 112.75, 110.55, 32.45, 31.78, 31.76, 29.13, 29.11, 20.07, 28.74, 28.27, 22.62, 14.06. <u>MS (ESI):</u> m/z calcd for $C_{13}H_{21}NO_3SNa$ $[M+Na]^+$: 294.1141; found: 294.1139.

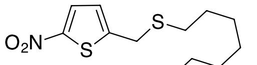

6

*2-Nitro-5-((octylthio)methyl)thiophene (**6**).* General procedures 1 and 2. Flash chromatography (PE:EtOAc/9:1) provided compound **6** (150 mg, 53% yield). <u>^{1}H NMR</u> (600 MHz, $CDCl_3$): δ 7.77 (d, *J* = 4.1 Hz, 1H), 6.91 (d, *J* = 4.1 Hz, 1H), 3.86 (s, 2H), 2.53 (t, *J* = 7.4, 2H), 1.61 – 1.54 (m, 2H), 1.40 – 1.33 (m, 2H), 1.32 – 1.20 (m, 8H), 0.88 (t, *J* = 7.0 Hz, 2H). <u>^{13}C NMR</u> (150 MHz, $CDCl_3$): δ 152.59, 128.65, 125.24, 32.12, 31.78, 31.04, 29.13, 29.12, 29.00, 28.76, 22.63, 14.08. <u>MS (ESI):</u> m/z calcd for $C_{13}H_{21}NO_2S_2Na$ $[M+Na]^+$: 310.0912; found: 310.0911.

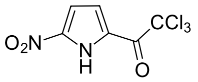

13

*2,2,2-Trichloro-1-(5-nitro-2-pyrrolyl)-1-ethanone (**13**). Product prepared according to literature.*[2] Pyrrole **12** (500 mg, 2.38 mmol) was dissolved in 2.5 mL acetic anhydride under N_2 atmosphere. The solution was cooled to -40 °C and conc HNO_3 (210 µL) was slowly added to the reaction and the temperature was allowed to warm to room temperature in 2 h. The mixture was allowed to cool to room temperature. After this time, the mixture was cooled to -40 °C and water was added, the resulting suspension was extracted with EtOAc (x3) and the organic phase was washed with brine, dried with anhydrous Na2SO_4, filtered and evaporated in vacuo. The crude product was purified by silica gel flash chromatography (PE:EtOAc) to give compound **13** (398 mg, 66% yield). <u>^{1}H NMR</u> (600 MHz, $CDCl_3$): δ 10.40 (s, 1H), 7.32 (d, J = 4.4 Hz, 1H), 7.13 (d, J = 4.4 Hz, 1H). <u>^{13}C NMR</u> (150 MHz, $CDCl_3$): δ 173.30, 124,03, 119.85, 110.25, 22.19.

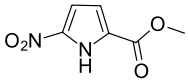

14

*Methyl 5-nitro-2-pyrrolecarboxylate (**14**).* Compound **13** (479 mg, 1.87 mmol) was cooled at 0°C and 15 mL of anhydrous methanol and K_2CO_3 (494 mg, 3.74 mmol) were added. The reaction was maintained at 0°C under magnetic stirring for 1 h. After the disappearance of the starting material, the crude was concentrated in vacuo, diluted with DCM and washed with H_2O and Brine. The organic layer was dried with anhydrous Na_2SO_4, filtered and evaporated in vacuo. The crude product was purified by silica gel flash chromatography (PE:EtOAc/4:1) to provide compound **14** (273 mg, 86% yield). <u>^{1}H NMR</u> (600 MHz, $CDCl_3$): δ 10.35 (s, 1H), 7.08

(d, J = 4.2 Hz, 1H), 6.90 (d, J = 4.2 Hz, 1H), 3.96 (s, 3H). ^{13}C NMR (150 MHz, $CDCl_3$): δ 160.06, 125.26, 115.32, 110,59, 52.68.

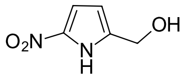

15

(5-Nitro-2-pyrrolyl)methanol (**15**). Under N_2 atmosphere, compound **14** (318 mg, 1.87 mmol) was dissolved in 10 mL of anhydrous THF and cooled to -60 °C. A 1 M solution of DIBAL (5.6 mL, 5.61 mmol) was carefully added and, after consumption of the starting material, the reaction was quenched with a saturated aqueous solution of NH4Cl. The crude product was extracted with EtOAc (x3) and washed with H_2O and brine. The organic layer was dried over anhydrous Na_2SO_4, filtered and concentrated in vacuo. The reaction mixture was purified by silica gel flash chromatography (PE:EtOAc/2:1) to give compound **15** (244 mg, 92% yield). ^{1}H NMR (600 MHz, CD_3OD): δ 7.03 (d, J = 4.0 Hz, 1H), 6.23 (d, J = 4.0 Hz, 1H), 4.56 (s, 2H). ^{13}C NMR (150 MHz, CD_3OD): δ 110.98, 108.53, 56.25. MS (ESI): m/z calcd for $C_5H_6N_2O_3Na$ $[M+Na]^+$: 165.0276; found: 165.0274.

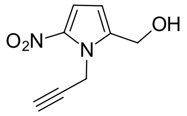

*[5-Nitro-1-(2-propynyl)-2-pyrrolyl]methanol (**16**)*. Compound **15** (110 mg, 0.775 mmol) was dissolved in 2 mL of anhydrous DMF under N_2 atmosphere and K_2CO_3 (214 mg, 1.55 mmol) was added. The suspension was heated at 40 °C for 1 h, then propargyl bromide (59 µL, 0.775 mmol) was added and the reaction was stirred for 16 h at 40 °C. After cooling to room temperature, H_2O was added and the crude product was extracted with EtOAc (x3) and washed with brine (x3). The organic layer was dried over anhydrous Na_2SO_4, filtered and concentrated in vacuo. The reaction mixture was purified by silica gel flash chromatography (PE:EtOAc/4:1) to give compound **16** (89 mg, 64% yield). ^{1}H NMR (600 MHz, $CDCl_3$): δ 7.20 (d, J = 4.3 Hz, 1H), 6.22 (d, J = 4.3 Hz, 1H), 5.34 (d, J = 2.3 Hz, 3H), 4.76 (d, J = 5.7 Hz, 3H), 1.91 (t, J = 5.8 Hz, 1H). ^{13}C NMR (150 MHz, $CDCl_3$): δ 139.71, 138.62, 114.26, 109.61, 73.18, 56.94, 35.72. MS (ESI): m/z calcd for $C_8H_8N_2O_3Na$ $[M+Na]^+$: 203.0433; found: 203.0431.

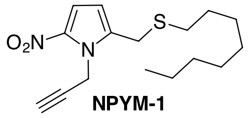

NPYM-1

5-Nitro-2-[(octylthio)methyl]-1-(2-propynyl)pyrrole (**NPYM-1**). General procedure. Flash chromatography (PE:EtOAc/9:1) provided compound **NPYM-1** (20 mg, 68 % yield). ^{1}H NMR (600 MHz, $CDCl_3$): δ 7.21 (d, J = 4.3 Hz, 1H), 6.13 (d, J = 4.3 Hz, 1H), 5.38 (d, J = 2.4 Hz, 2H), 3.80 (s, 2H), 2.47 (t, J = 7.2 Hz, 2H), 2.34 (s, 1H), 1.59 – 1.52 (m, 2H), 1.39 – 1.32 (m, 2H), 1.32 – 1.21 (m, 8H), 0.89 (t, J = 7.2 Hz, 3H). ^{13}C NMR (150 MHz, $CDCl_3$): δ

136.69, 114.48, 110.36, 73.15, 35.28, 31.78, 31.68, 29.14, 29.13, 29.09, 28.78, 27.36, 22.63, 14.08. MS (ESI): m/z calcd for $C_{16}H_{24}N_2O_2SNa$ $[M+Na]^+$: 331.1456; found: 331.1455.

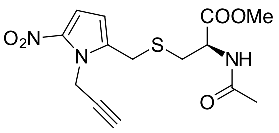

NPYM-20

Methyl N-acetyl-S-((5-nitro-1-(2-propynyl)-2-pyrrolyl)methyl)-L-cysteinate **(NPYM-20):** To a solution of methyl acetyl L-cysteinate (32 mg; 0.18 mmol) in THF (8 mL) was added bromide **19** (0.22 mmol) and DIPEA (64 µL, 0.37 mmol), then the mixture was stirred for 16 h at room temperature. The crude product was diluted with EtOAc, then washed with H_2O and brine. The organic layer was dried over anhydrous Na_2SO_4, filtered and concentrated in vacuo. The reaction mixture was purified by silica gel flash chromatography (PE:EtOAc/50:50) to give the product (44 mg, 78% yield). ^{1}H NMR: (400 MHz, $CDCl_3$) δ 7.16 (d, *J* = 4.3 Hz, 1H), 6.29 (d, *J* = 7.2 Hz, 1H), 6.17 (d, *J* = 4.3 Hz, 1H), 5.35 – 5.19 (m, 2H), 4.80 (dd, *J* = 12.9, 5.5 Hz, 1H), 3.82 (s, 2H), 3.74 (s, 3H), 2.96 (dd, *J* = 14.0, 5.1 Hz, 1H), 2.82 (dd, *J* = 14.0, 5.9 Hz, 1H), 2.33 (s, 1H), 2.01 (s, 3H). ^{13}C NMR (150 MHz, CD_3OCl_3): δ 171.06, 169.96, 137.74, 135.54, 114.48, 110.87, 73.38, 52.83, 51.56, 35.31, 33.62, 29.64, 27.60, 23.07. MS (ESI): m/z calcd for $C_{14}H_{17}N_3O_5SNa$ $[M+Na]^+$: 362.0787; found: 362.0786.

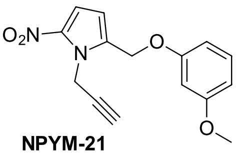

NPYM-21

2-[(m-Methoxyphenoxy)methyl]-5-nitro-1-(2-propynyl)pyrrole (**NPYM-21**). To a solution of 3-methoxyphenol (29 mg, 0.23 mmol) in dry acetone (5 mL) was added K_2CO_3 (98 mg, 0.71 mmol) and the reaction was refluxed under magnetic stirring for 20 min. Then the mixture was cooled to room temperature and the bromo derivative **19** (0.28 mmol) was added. The reaction was refluxed for 16 h then cooled to room temperature and concentrated *in vacuo*. The crude was purified by silica gel flash chromatography (PE:EtOAc/70:30) in order to obtain the product (51 mg, 78% yield). ^{1}H NMR: (600 MHz, $CDCl_3$) δ 7.24 – 7.16 (m, 2H), 6.64 (dd, J = 8.2, 1.7 Hz, 1H), 6.62 – 6.59 (m, 1H), 6.57 (dd, J = 8.1, 1.7 Hz, 1H), 6.40 (d, J = 4.3 Hz, 1H); 5.30 (s, J = 2.3 Hz, 2H), 5.18 (s, 2H), 3.77 (s, 3H), 2.81 (s, J = 2.4 Hz, 1H). ^{13}C NMR (150 MHz, $CDCl_3$): δ 161.10, 159.13, 135.75, 129.65, 113.34, 110.33, 106.95, 106.77, 101.18, 77.15, 72.90, 61.45, 54.34, 35.54. MS (ESI): m/z calcd for $C_{15}H_{14}N_2O_4Na$ $[M+Na]^+$: 309.0852; found: 309.0850.

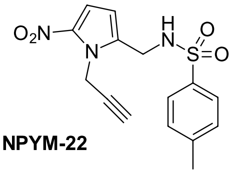

NPYM-22

[5-Nitro-1-(2-propynyl)-2-pyrrolyl](tosylamino)methane (**NPYM-22**). To a solution of 2-methyl-benzenesulfonamide (79 mg; 0.46 mmol) in THF (5 mL), bromide **19** (0.55 mmol) and DIPEA (16 µL, 0.92 mmol) were added, then the mixture was refluxed for 16 h. The crude was diluted with DCM then washed with H_2O and brine. The organic layer was dried over anhydrous Na_2SO_4, filtered and concentrated *in vacuo*. The reaction mixture was purified by silica gel flash chromatography (PE:EtOAc/60:40) in order to obtain the product (118 mg, 77% yield). ^{1}H NMR: (600 MHz, $CDCl_3$) δ 7.76 (d, J = 8.3 Hz, 2H), 7.74 (d, J = 1.9 Hz, 1H), 7.38 (d, J = 8.0 Hz, 2H), 6.55 (d, J = 1.9 Hz, 1H), 4.81 (d, J = 2.6 Hz, 2H), 4.11 (d, J = 6.3 Hz, 2H), 2.61 (s, 1H), 2.49 (s, 3H). ^{13}C NMR (150 MHz, $CDCl_3$): δ 144.4, 143.7, 135.8, 130.1, 129.8, 127.2, 127.0, 126.5, 123.1, 106.9, 76.3, 75.6, 39.0, 37.6, 21.6. MS (ESI): m/z calcd for $C_{15}H_{15}N_3O_4SNa$ $[M+Na]^+$: 356.0681; found: 356.0680.

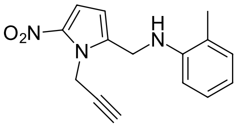

NPYM-23

o-({[5-Nitro-1-(2-propynyl)-2-pyrrolyl]methyl}amino)toluene **(NPYM-23).** To a solution of *o*-toluidine (60 mg; 0.56 mmol) in THF (5 mL) bromide **19** (0.28 mmol) and DIPEA (97 µL, 0.56 mmol) were added, then the mixture was refluxed for 16 h. The crude was diluted with DCM then washed with H_2O and brine. The organic layer was dried over anhydrous Na_2SO_4, filtered and concentrated *in vacuo*. The reaction mixture was purified by silica gel flash chromatography (PE:EtOAc/60:40) in order to obtain the product (70 mg, 93% yield). ^{1}H NMR: (600 MHz, $CDCl_3$) δ 7.24 (d, J = 3.8 Hz, 1H), 7.17 (t, J = 7.5 Hz, 1H), 7.12 (d, J = 7.1 Hz, 1H), 6.78 (t, J = 7.3 Hz, 1H), 6.69 (d, J = 7.9 Hz, 1H), 6.25 (d, J = 3.9 Hz, 1H); 5.33 (d, J = 1.5 Hz, 2H), 4.45 (s, 2H), 3.74 (s, 1H), 2.38 (s, 1H), 2.19 (s, 3H). ^{13}C NMR (150 MHz, $CDCl_3$): δ 144.93, 137.86, 130.43, 127.24, 122.96, 118.63, 114.67, 110.24, 109.85, 73.39, 40.90, 35.75, 29.71, 17.54. MS (ESI): m/z calcd for $C_{15}H_{15}N_3O_2Na$ $[M+Na]^+$: 292.1062; found: 292.1063.

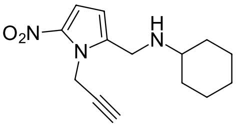

NPYM-24

1-({[5-Nitro-1-(2-propynyl)-2-pyrrolyl]methyl}amino)cyclohexane (**NPYM-24**). To a solution of cyclohexylamine (26 µL; 0.23 mmol) in THF (5 mL) bromide **19** (0.28 mmol) and DIPEA (97 µL, 0.56 mmol) were added, then the mixture was refluxed for 16 h. The crude was diluted with DCM then washed with H_2O and brine. The organic layer was dried over anhydrous Na_2SO_4, filtered and concentrated *in vacuo*. The

reaction mixture was purified by silica gel flash chromatography (PE:EtOAc/90:10) in order to obtain the product (49 mg, 95% yield). ^{1}H NMR: (600 MHz, $CDCl_3$) δ 7.19 (d, *J* = 4.2 Hz, 1H), 6.12 (d, *J* = 4.1 Hz, 1H), 5.46 (d, *J* = 2.0 Hz, 2H), 3.89 (s, 2H), 2.56 – 2.43 (m, 1H), 1.91 (d, *J* = 10.3 Hz, 2H), 1.74 (dd, *J* = 9.6, 3.4 Hz, 2H), 1.62 (dd, *J* = 9.0, 3.4 Hz, 1H), 1.35 – 1.16 (m, 4H), 1.15 – 1.02 (m, 2H). ^{13}C NMR (150 MHz, CD_3OCl_3): δ 139.75, 137.45, 114.47, 109.35, 77.88, 72.60, 56.32, 42.96, 35.36, 33.34, 29.67, 26.03, 24.80. MS (ESI): m/z calcd for $C_{14}H_{19}N_3O_2Na$ $[M+Na]^+$: 284.1375; found: 248.1373.

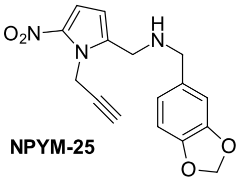

NPYM-25

{[(2H-1,3-Benzodioxol-5-yl)methyl]amino}[5-nitro-1-(2-propynyl)-2-pyrrolyl]methane **(NPYM-25).** To a solution of piperonylamine (22 µL; 0.23 mmol) in THF (5 mL) the bromide **19** (0.28 mmol) and DIPEA (97 µL, 0.56 mmol) were added then the mixture was refluxed for 16 h. The crude was diluted with DCM then washed with H_2O and brine. The organic layer was dried over anhydrous Na_2SO_4, filtered and concentrated *in vacuo*. The reaction mixture was purified by silica gel flash chromatography (PE:EtOAc/85:15) in order to obtain the product (56 mg, 79% yield). ^{1}H NMR: (600 MHz, $CDCl_3$) δ 7.19 (d, *J* = 4.2 Hz, 1H), 6.83 (s, 1H), 6.76 (s, 2H), 6.13 (d, J = 4.2 Hz, *1*H), 5.95 (s, 2H), 5.41 (d, *J* = 2.1 Hz, 2H), 3.86 (s, 2H), 3.73 (s, 2H), 2.30 (s, 1H). ^{13}C NMR (150 MHz, CD_3OCl_3): δ 147.9, 146.8, 138.9, 137.6, 133.3, 120.8, 113.8, 113.8, 109.3, 107.6, 101.0, 73.6, 71.9, 53.1, 44.8, 35.5. MS (ESI): m/z calcd for $C_{16}H_{15}N_3O_4Na$ $[M+Na]^+$: 336.0960; found: 336.0962.

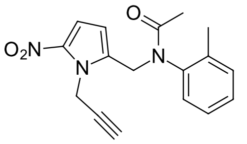

NPYM-26

N-{[5-Nitro-1-(2-propynyl)-2-pyrrolyl]methyl}-N-o-tolylacetamide ((**NPYM-26**)*):* Compound **NPYM-23** (6 mg, 0.022 mmol) was dissolved in dry THF (3 mL) and cooled to 0 °C under N_2 atmosphere and magnetic stirring. Et_3N (4.5 mg; 0.044 mmol) and acetyl chloride (2.6 mg, 0.033 mmol) were added and the mixture stirred at 0 °C for 1 h. The crude was diluted with DCM then washed with H_2O and brine. The organic layer was dried over anhydrous Na_2SO_4, filtered and concentrated *in vacuo*. The reaction mixture was purified by silica gel flash chromatography (PE:EtOAc/70:30) in order to obtain the product (5 mg, 75% yield). ^{1}H NMR: (600 MHz, $CDCl_3$) δ 7.30 (t, J = 8.5 Hz, 1H), 7.16 (t, J = 6.7 Hz, 1H), 7.12 (d, J = 4.3 Hz, 1H), 6.82 (d, J = 7.8 Hz, 1H), 5.77 (d, J = 4.3 Hz, 1H), 5.44 (dd, J = 24.6, 9.0 Hz, 2H), 5.30 (dd, J = 17.6, 2.3 Hz, 1H), 4.36 (d, J = 15.4 Hz, 1H), 2.32 (m, 1H), 2.14 (s, 3H), 1.82 (s, 3H). ^{13}C NMR (150 MHz, CD_3OCl_3): δ 170.68, 139.68, 136.08, 135.60, 131.68, 129.06, 127.41, 114.37, 111.61, 73.03, 42.37, 36.36, 21.97, 17.20. MS (ESI): m/z calcd for $C_{17}H_{17}N_3O_3Na$ $[M+Na]^+$: 334.1168; found: 334.1167.

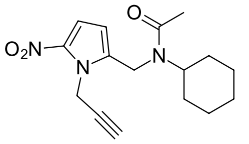

NPYM-27

N-Cyclohexyl-N-{[5-nitro-1-(2-propynyl)-2-pyrrolyl]methyl}acetamide **(NPYM-27):** Compound **NPYM-24** (20 mg, 0.077 mmol) was dissolved in dry THF (5 mL) and cooled to 0 °C under N_2 atmosphere and magnetic stirring. Et_3N (21 µL; 0.153 mmol) and acetyl chloride (8.2 µL, 0.115 mmol) were added and the mixture was stirredat 0 °C for 1 h. The crude was diluted with DCM then washed with H_2O and brine. The organic layer was dried over anhydrous Na_2SO_4, filtered and concentrated *in vacuo*. The reaction mixture was purified by silica gel flash chromatography (PE:EtOAc/65:35) in order to obtain the product (22 mg, 97% yield). ^{1}H NMR: (600 MHz, $CDCl_3$) δ 7.19 (d, *J* = 4.2 Hz, 1H), 6.83 (s, 1H), 6.76 (s, 2H), 6.13 (d, *J* = 4.2 Hz, 1H), 5.95 (s, 2H), 5.41 (d, *J* = 2.1 Hz, 2H), 3.86 (s, 2H), 3.73 (s, 2H), 2.30 (s, 1H). ^{13}C NMR (150 MHz, CD_3OCl_3): δ 170.80, 139.15, 136.96, 114.80, 109.03, 77.62, 73.16, 58.54, 37.07, 35.75, 31.72, 30.64, 29.66, 25.91, 25.16, 21.86. MS (ESI): m/z calcd for $C_{16}H_{21}N_3O_3Na$ $[M+Na]^+$: 326.1481; found: 326.1479.

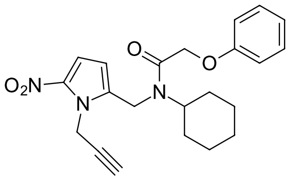

NPYM-28

N-Cyclohexyl-N-{[5-nitro-1-(2-propynyl)-2-pyrrolyl]methyl}phenoxyacetamide (**NPYM-28**). Compound **NPYM-24** (18 mg, 0.067 mmol) was dissolved in dry THF (5 mL) and cooled to 0 °C under N_2 atmosphere and magnetic stirring. Et_3N (19 µL; 0.14 mmol) and phenoxyacetyl chloride (14 µL, 0.103 mmol) were added and the mixture was stirred at 0 °C for 1 h. Then the crude was diluted with DCM, washed with H_2O and brine. The organic layer was dried over anhydrous Na_2SO_4, filtered and concentrated *in vacuo*. The reaction mixture was purified by silica gel flash chromatography (PE:EtOAc/80:20) to obtain the product (21 mg, 81% yield).^{1}H NMR: (600 MHz, $CDCl_3$) δ 7.33 – 7.21 (m, 2H), 7.11 (d, *J* = 4.1 Hz, 1H), 7.02 – 6.85 (m, 3H), 5.85 (d, *J* = 4.0 Hz, 1H), 5.29 (s, 2H), 4.77 (s, 2H), 4.56 (s, 3H), 3.87 (m, 1H), 1.80 (m, 5H), 1.65 (d, *J* = 12.4 Hz, 2H), 1.50 – 1.26 (m, 5H), 1.12 – 0.94 (m, 1H). ^{13}C NMR (150 MHz, CD_3OCl_3): δ 171.0, 168.6, 157.7, 138.3, 136.9, 129.7, 129.6, 121.8, 114.8, 114.6, 108.9, 73.4, 67.7, 64.9, 57.5, 37.7, 35.6, 31.9, 30.3, 29.7, 25.7, 25.1. MS (ESI): m/z calcd for $C_{22}H_{25}N_3O_4Na$ $[M+Na]^+$: 418.1743; found: 418.1744.

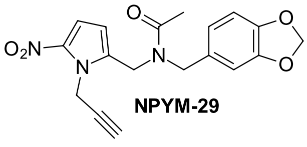

NPYM-29

N-[(2H-1,3-Benzodioxol-5-yl)methyl]-N-{[5-nitro-1-(2-propynyl)-2-pyrrolyl]methyl}acetamide (**NPYM-29**). Compound **NPYM-25** (20 mg, 0.064 mmol) was dissolved in dry THF (5 mL) and cooled to 0 °C under N_2 atmosphere and magnetic stirring. Et_3N (18 µL; 0.13 mmol) and acetyl chloride (7 µL, 0.096 mmol) were added into the reaction and the mixture was stirred at 0 °C for 1 h. The crude was diluted with DCM then washed with H_2O and brine. The organic layer was dried over anhydrous Na_2SO_4, filtered and concentrated *in vacuo*. The reaction mixture was purified by silica gel flash chromatography (PE:EtOAc/70:30) in order to obtain the product (23 mg, 96% yield). ^{1}H NMR: (600 MHz, $CDCl_3$) δ 7.19 (d, *J* = 3.8 Hz, 1H), 6.81 (d, *J* = 7.7 Hz, 1H), 6.65 – 6.56 (m, 2H), 6.05 (d, *J* = 3.7 Hz, 1H), 5.99 (s, 2H), 5.24 (s, 2H), 4.68 (s, 2H), 4.33 (s, 2H), 2.28 (s, 3H), 2.01 (s, 1H). ^{13}C NMR (150 MHz, CD_3OCl_3): δ 170.9, 148.5, 147.5, 137.9, 135.7, 129.0, 119.8, 114.2, 111.2, 108.7, 106.8, 101.4, 72.5, 67.9, 50.0, 38.9, 36.3, 21.5. MS (ESI): m/z calcd for $C_{18}H_{17}N_3O_5Na$ $[M+Na]^+$: 378.1066; found: 378.1065.

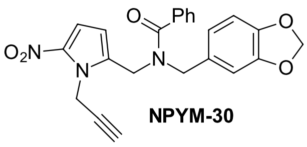

NPYM-30

N-[(2H-1,3-Benzodioxol-5-yl)methyl]-N-{[5-nitro-1-(2-propynyl)-2-pyrrolyl]methyl}benzamide (**NPYM-30**). Compound **NPYM-25** (18 mg, 0.057 mmol) was dissolved in dry THF (5 mL) and cooled to 0 °C under N_2 atmosphere and magnetic stirring. Et_3N (16 µL; 0.115 mmol) and benzoyl chloride (10 µL, 0.086 mmol) were added and the mixture was stirred at 0 °C for 1 h. The crude was diluted with DCM then washed with H_2O and brine. The organic layer was dried over anhydrous Na_2SO_4, filtered and concentrated *in vacuo*. The reaction mixture was purified by silica gel flash chromatography (PE:EtOAc/70:30) in order to obtain the product (22 mg, 89% yield). ^{1}H NMR: (400 MHz, $CDCl_3$) δ 7.63 – 7.33 (m, 5H), 7.21 (d, J = 4.1 Hz, 1H), 6.77 (d, J = 7.9 Hz, 1H), 6.61 (s, 2H), 6.04 (s, 1H), 5.97 (s, 2H), 5.29 (sb, 2H), 4.77 (sb, 2H), 4.39 (sb, 2H), 2.27 (s, 1H). ^{13}C NMR (150 MHz, CD_3OCl_3): δ 171.9, 148.4, 147.5, 137.8, 135.3, 135.0, 130.2, 128.6, 126.9, 120.5, 114.4, 111.2, 108.6, 107.2, 101.3, 73.2, 67.9, 51.4, 38.6, 36.2, 30.3, 29.7, 25.6. MS (ESI): m/z calcd for $C_{23}H_{19}N_3O_5Na$ $[M+Na]^+$: 440.1223; found: 440.1224.

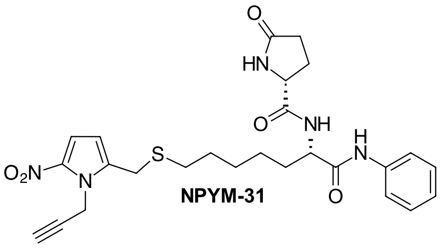

NPYM-31

Compound **NPYM-31.** ST7612AA1 (50 mg, 0.123 mmol) was dissolved in 2.5 mL of MeOH in a round-bottom flask. The solution was degassed with three cycles of argon/vacuum and CH_3SNa (8.6 mg, 0.123 mmol) as a solution 1M in degassed MeOH was added. The reaction was stirred for 30 min, further degassed with argon and evaporated *in vacuo* to lead the free thiol. Following the general procedure for thioether, compound **NPYM-OH** (24 mg, 0.133 mmol) was transformed into **19** (0.123 mmol), solubilized in dry THF (2 mL) and added to the thiol. The reaction was stirred under argon for 16 h at room temperature. The crude was evaporated *in vacuo* and purified by silica gel flash chromatography (DCM:MeOH/8:2) to provide compound **NPYM-31** (42 mg, 66% yield). ^{1}H NMR (600 MHz, CD_3OD): δ 7.55 (d, J = 7.7 Hz, 2H), 7.31 (t, J = 7.9 Hz, 2H), 7.18 (d, J = 4.3 Hz, 1H), 7.11 (t, J = 7.3 Hz, 1H), 6.22 (d, J = 4.2 Hz, 1H), 5.33 (d, J = 2.3 Hz, 2H), 4.47 (dd, J = 8.3, 5.8 Hz, 1H), 4.27 (dd, J = 8.6, 4.7 Hz, 1H), 3.87 (s, 2H), 3.31 (s, 5H), 2.53 – 2.38 (m, 5H), 2.36 – 2.27 (m, 2H), 2.12 – 2.03 (m, 1H), 1.79 – 1.71 (m, 1H), 1.64 – 1.54 (m, 2H), 1.50 – 1.37 (m, 4H). ^{13}C NMR (150 MHz, CD_3OD): δ 180.1, 173.5, 171.1, 138.0, 137.5, 128.4, 124.1, 120.0, 113.8, 110.0, 77.2, 72.9, 56.6, 53.9, 46.0, 35.0, 31.9, 30.9, 29.1, 28.7, 27.9, 26.4, 26.0, 25.9, 25.4, 25.1. MS (ESI): m/z calcd for $C_{26}H_{31}N_5O_5SNa$ $[M+Na]^+$: 548.1944; found: 548.1944.

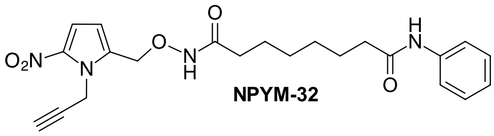

NPYM-32

Compound ***NPYM-32***. To a solution of SAHA (24 mg, 0.093 mmol) in MeOH (1 mL) was added an aqueous solution of NaOH 10 N (11 mg; 0.279 mmol) and the reaction was stirred at room temperature for 10 min. Then bromide **19** (0.11 mmol,) was added and the reaction was stirred until consumption of the bromide, monitored by TLC. An aqueous solution of HCl 1N (28 µL) was added in the reaction. The crude was diluted with DCM then washed with H_2O and brine. The organic layer was dried over anhydrous Na_2SO_4, filtered and concentrated *in vacuo*. The reaction mixture was purified by silica gel flash chromatography (DCM:MeOH/95:5) in order to obtain the product (21 mg, 52% yield). ^{1}H NMR: (600 MHz, $CDCl_3$) δ 7.54 (d, J = 7.8 Hz, 2H), 7.29 (t, J = 7.9 Hz, 2H), 7.19 (d, J = 4.2 Hz, 1H), 7.08 (t, J = 7.4 Hz, 1H), 6.36 (d, J = 4.2 Hz, 1H); 5.49 (d, J = 2.0 Hz, 2H), 4.94 (s, 2H), 2.80 (s, 1H); 2.37 (t, J = 7.4 Hz, 2H), 2.09 – 2.06 (m, 2H), 1.70 – 1.68 (m, 2H), 1.62 – 1.60 (m, 2H), 1.39 – 1.35 (m, 4H). ^{13}C NMR (150 MHz, $CDCl_3$): δ 173.18, 171.91, 138.49, 138.20, 133.84, 128.37, 123.72, 199.87, 112.98, 111.88, 84.11, 77.31, 72.92, 67.98, 36.43, 35.50, 32.19, 28.47, 28.38, 25.27, 24.94. MS (ESI): m/z calcd for $C_{22}H_{25}N_4O_5$ $[M-1]^-$: 425.1825; found: 425.1824.

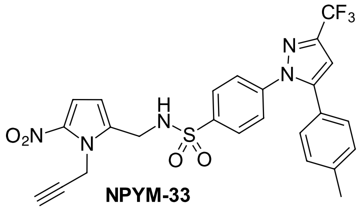

NPYM-33

Compound **NPYM-33**. In a Schlenk tube under Ar, a solution of Celecoxib (106 mg, 0.28 mmol) in 2 mL of dry THF was cooled to -78 °C and KHMDS (0.380 mL 1M in THF) was added strirred at -78 °C for 45 min. A solution of bromide **19** (0.32 mmol) in THF was added at -50 °C and the reaction was allowed to reach room temperature and stirred for 16 h. A NH_4Cl saturated solution was added the crude was extracted with DCM (x3) and washed with brine. The organic layer was dried over anhydrous Na_2SO_4, filtered and concentrated *in vacuo*. The reaction mixture was purified by silica gel flash chromatography (EP:EtOAc/70:30) in order to obtain the product (80 mg, 53% yield). ^{1}H NMR: (600 MHz, $CDCl_3$) δ 7.80 (d, J = 8.6 Hz, 2H), 7.66 (d, J = 1.9 Hz, 1H), 7.60 (d, J = 8.6 Hz, 2H), 7.24 (d, J = 8.1 Hz, 2H), 7.17 (d, J = 8.1 Hz, 2H), 6.79 (s, 1H), 6.40 (d, J = 1.8 Hz, 1H), 4.73 (d, J = 2.6 Hz, 2H), 4.30 (s, 2H), 2.58 (s, 1H), 2.42 (s, 3H). ^{13}C NMR (150 MHz, $CDCl_3$): δ 145.4, 143.5, 140.1, 136.1, 136.1, 129.9, 128.8, 128.4, 126.0, 125.8, 123.1, 107.4, 76.3, 75.5, 43.8, 37.6, 21.4. MS (ESI): m/z calcd for $C_{25}H_{19}F_3N_5O_4S$ $[M-1]^-$: 542.1111; found: 542.1108.

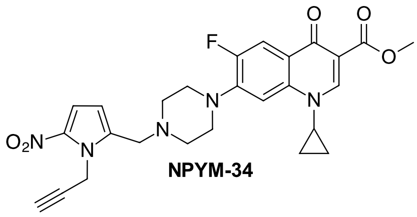

NPYM-34

Compound (**NPYM-34**). To a solution of Ciprofloxacin methyl ester **34** (96 mg; 0.28 mmol) in THF (10 mL) bromide **19** (0.55 mmol) and DIPEA (106 µL, 0.61 mmol) were added, then the mixture was refluxed for 16 h. The crude was diluted with DCM then washed with H_2O and brine. The organic layer was dried over anhydrous Na_2SO_4, filtered and concentrated *in vacuo*. The product was purified by trituration with EtOAc and filtration (126 mg, 89% yield). ^{1}H NMR: (600 MHz, $CDCl_3$) δ 8.40 (s, 1H), 7.99 (d, J = 2.1 Hz, 1H), 7.72 (d, J = 13.5 Hz, 1H), 7.40 (d, J = 7.4 Hz, 1H), 6.66 (d, J = 2.1 Hz, 1H), 5.02 (d, J = 2.5 Hz, 2H), 3.69 (s, 3H), 3.66 – 3.50 (m, 4H), 3.26 – 3.16 (m, 4H), 2.60 – 2.50 (m, 4H), 1.20 (t, J = 6.5 Hz, 2H), 1.11 – 0.97 (m, 2H). ^{13}C NMR (150 MHz, $CDCl_3$): δ 172.0, 165.4, 148.7, 144.2, 138.5, 135.1, 130.7, 124.2, 122.3, 122.2, 112.1, 111.8, 109.4, 106.7, 106.1, 78.5, 77.6, 53.0, 52.4, 51.7, 50.0, 37.5, 35.2, 8.0. MS (ESI): m/z calcd for $C_{26}H_{26}FN_5O_5Na$ $[M+Na]^+$: 530.1816; found: 530.1817.

Synthesis of NPYM-35

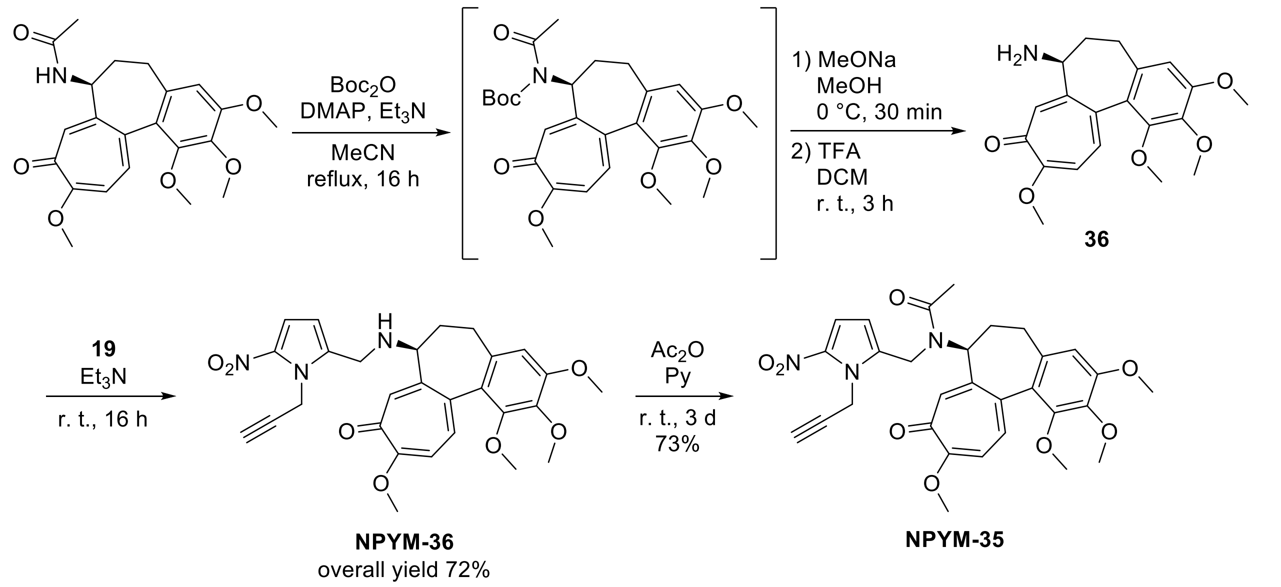

Compound **NPYM-36***.* Product 36 was prepared according to literature.[3] To a solution of Colchicine (1 g, 2.5 mmol) in MeCN (10 mL), Boc_2O (2.307 g, mmol), DMAP (304 mg, mmol) and Et_3N (673 mL, mmol) were added and the resulting solution was refluxed for 20 h. After cooled to room temperature, the crude was diluted with $CHCl_3$ washed with water, a solution of citric acid (10% p/p) and brine. The organic layer was dried over anhydrous Na_2SO_4, filtered and concentrated *in vacuo*. The reaction mixture was used without further purification for the next step by dissolving in MeOH (13.5 mL) and cooling to 0 °C in ice bath. MeONa (461 mg, 8.5 mmol) was added and the reaction was stirred for 30 min. After the disappearance of the starting material, water was added. The crude was extracted with EtOAc (x3) then washed with H_2O and brine. The organic layer was dried over anhydrous Na_2SO_4, filtered and concentrated *in vacuo*. The product was resolubilized in DCM (12 mL) and TFA (5.8 mL) was added. After 3 h at room temperature, the reaction was quenched with NaOH 1N until pH=10. The crude was extracted with DCM (x3) then washed with H_2O and brine. The organic layer was dried over anhydrous Na_2SO_4, filtered and concentrated *in vacuo*. The reaction mixture was purified by silica gel flash chromatography (DCM:MeOH/90:10) in order to obtain product **36**.

36 (119 mg; 0.33 mmol) in THF (5 mL) was added the bromide **19** (0.28 mmol) and Et_3N (46 µL, 0.33 mmol), then the mixture was stirred at room temperature for 16 h. The crude was diluted with DCM then washed with H_2O and brine. The organic layer was dried over anhydrous Na_2SO_4, filtered and concentrated *in vacuo*. The reaction mixture was purified by silica gel flash chromatography (DCM:MeOH/99:01) in order to obtain the product (105 mg, 72% yield). ^{1}H NMR (600 MHz, $CDCl_3$): δ 7.60 (s, 1H), 7.22 (d, *J* = 10.6 Hz, 1H), 7.04 (d, *J* = 4.2 Hz, 1H), 6.81 (d, *J* = 10.7 Hz, 1H), 6.51 (s, 1H), 5.93 (d, *J* = 4.1 Hz, 1H), 5.30 (s, 2H), 3.99 (s, 3H), 3.89 (s, 6H), 3.85 – 3.79 (m, 1H), 3.64 (s, 3H), 3.60 (d, *J* = 14.3 Hz, 1H), 3.40 (dd, *J* = 10.8, 6.2 Hz, 1H), 2.46 (dd, *J* = 13.4, 6.2 Hz, 1H), 2.36 (td, *J* = 13.2, 6.8 Hz, 1H), 2.29 – 2.17 (m, 2H), 1.68 – 1.64 (m, 1H). ^{13}C NMR (150 MHz, $CDCl_3$): δ 179.42, 163.95, 153.45, 150.70, 150.12, 141.25, 138.13, 137.52, 136.72, 135.10, 134.85, 131.59, 125.11,

114.24, 111.88, 109.79, 107.21, 72.98, 66.39, 61.14, 61.01, 59.89, 56.32, 56.03, 43.48, 38.76, 35.52, 30.26. MS (ESI): m/z calcd for $C_{28}H_{29}N_3O_7Na$ $[M+Na]^+$: 542.1904; found: 542.1903.

*Compound **NPYM-35**:* **NPYM-36** (32 mg, 0.06 mmol) was dissolved in pyridine (100 mL) and Ac_2O (66 mL) was added. The reaction was stirred at room temperature for 3 days, then concentrated *in vacuo*. The crude was purified by silica gel flash chromatography (DCM:MeOH/99:01) in order to obtain the product (24 mg, 73% yield). ^{1}H NMR (600 MHz, $CDCl_3$): δ 7.35 (d, *J* = 10.2 Hz, 1H), 7.29 – 7.14 (m, 2H), 6.85 (d, *J* = 10.6 Hz, 1H), 6.49 (s, 1H), 6.03 (s, 1H), 5.30 (d, *J* = 3.5 Hz, 1H), 5.12 (s, 2H), 4.91 (d, *J* = 18.4 Hz, 1H), 4.00 (s, 3H), 3.95 (s, 3H), 3.89 (s, 3H), 3.73 (s, 3H), 2.53 – 2.52 (m, 1H), 2.48 – 2.33 (m, 2H), 2.17 (s, 1H), 2.08 (s, 3H), 1.82 (s, 1H). ^{13}C NMR (150 MHz, $CDCl_3$): δ 179.36, 171.56, 164.10, 153.54, 151.58, 151.32, 141,97, 137.87, 137.66, 136.26, 135.45, 133.47, 129.98, 125.81, 114.93, 112.44, 108.04, 107.27, 74.48, 61.68, 61.42, 57.64, 56.42, 56.09, 43.81, 35.57, 33.14, 29.73, 22.00. MS (ESI): m/z calcd for $C_{30}H_{31}N_3O_8Na$ $[M+Na]^+$: 584.2009; found: 584.2010.

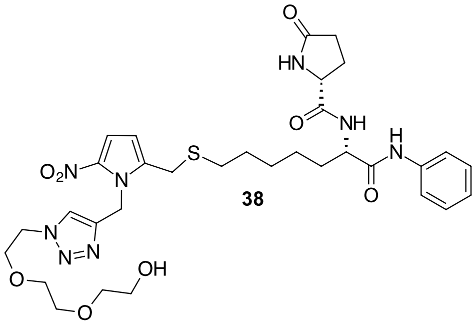

*Compound **38**.* **NPYM-31** (22 mg, 0.04 mmol) and azide **37** (11 mg, 0.04 mmol) were dissolved in dry DMF (2 mL) in a round-bottom flask under magnetic stirring and Ar atmosphere. The solution was degassed with three cycles of argon/vacuum. To this solution, a freshly prepared aqueous mixture (1 mL) of $Cu(OAc)_2$ (2.5 mg, 0.013 mmol) and sodium ascorbate (5 mg, 0.025 mmol), previously degassed was added dropwise. The reaction mixture was degassed and left to stir under argon at room temperature for 16 h. The solvent was evaporated and the crude reaction mixture was purified by silica gel flash chromatography (CH_2Cl_2:MeOH/8:2) to provide compound **38** (18 mg, 64% yield) as a pale yellow oil. ^{1}H NMR (600 MHz, MeOD) δ 7.93 (s, 1H), 7.55 (d, *J* = 7.7 Hz, 2H), 7.31 (t, *J* = 7.9 Hz, 2H), 7.19 (d, *J* = 4.3 Hz, 1H), 7.10 (t, *J* = 7.4 Hz, 1H), 6.24 (d, *J* = 4.3 Hz, 1H), 5.80 (s, 2H), 4.53 (t, *J* = 5.0 Hz, 2H), 4.48 (dd, *J* = 8.5, 5.8 Hz, 1H), 4.27 (dd, *J* = 8.6, 4.8 Hz, 1H), 4.01 (s, 2H), 3.84 (t, *J* = 5.0 Hz, 2H), 3.69 – 3.61 (m, 8H), 3.57 (dd, *J* = 9.4, 4.6 Hz, 7H), 3.51 – 3.47 (m, 2H), 2.53 (t, *J* = 7.2 Hz, 2H), 2.50 – 2.38 (m, 2H), 2.36 – 2.27 (m, 1H), 2.13 – 2.04 (m, 1H), 1.90 – 1.81 (m, 1H), 1.80 – 1.71 (m, 1H), 1.63 – 1.55 (m, 2H).^{13}C NMR (150 MHz, CD_3OD): δ 180.1, 173.5, 171.1, 143.2, 138.2, 138.1, 128.4, 124.1, 120.0, 114.0, 110.0, 72.3, 70.1, 70.0, 68.9, 60.8, 60.7, 56.6, 54.0, 50.0, 40.6, 31.8, 31.1, 29.3, 29.1, 28.7, 27.9, 27.4, 26.8, 25.4, 25.1. MS (ESI): m/z calcd for $C_{37}H_{51}N_8O_{10}SNa$ $[M+Na]^+$: 822.3347; found: 822.3345.

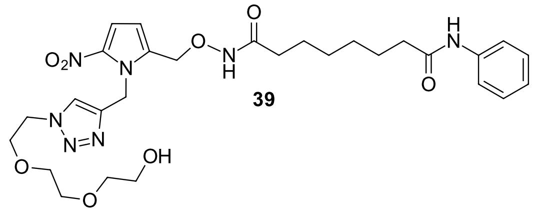

39

*Compound **39**.* **NPYM-32** (10 mg, 0.02 mmol) and azide **37** (4 mg, 0.02 mmol) were dissolved in dry DMF (2 mL) in a round-bottom flask under magnetic stirring and Ar atmosphere. The solution was degassed with three cycles of argon/vacuum. To this solution, a freshly prepared aqueous mixture (1 mL) of $Cu(OAc)_2$ (1.5 mg, 0.007 mmol) and sodium ascorbate (3 mg, 0.014 mmol), previously degassed, was added dropwise. The reaction mixture was degassed and left to stir under argon at room temperature for 16 h. The solvent was evaporated and the crude reaction mixture was purified by silica gel flash chromatography (CH_2Cl_2:MeOH/9.5:0.5) to provide compound **39** (6 mg, 53% yield) as a pale yellow oil. ^1H NMR (600 MHz, MeOD): δ 7.96 (s, 1H), 7.51 (d, J = 7.6 Hz, 2H), 7.29 – 7.23 (m, 2H), 7.16 (d, J = 4.3 Hz, 1H), 7.05 (t, J = 7.5 Hz, 1H), 6.34 (d, J = 4.2 Hz, 1H), 5.90 (s, 2H), 5.07 (s, 2H), 4.53 – 4.44 (m, 2H), 3.86 – 3.77 (m, 2H), 3.63 – 3.58 (m, 6H), 3.50 – 3.44 (m, 2H), 2.34 (t, J = 7.5 Hz, 2H), 2.06 (t, J = 7.3 Hz, 2H), 1.72 – 1.62 (m, 2H), 1.62 – 1.54 (m, 2H), 0.92 – 0.83 (m, 4H). ^{13}C NMR (150 MHz, CD_3OD): δ 183.1, 181.5, 166.1, 161.3, 151.5, 133.5, 119.3, 117.3, 83.1, 74.3, 73.5, 72.2, 71.4, 70.0, 68.9, 60.7, 59.3, 49.9, 43.3, 29.3, 23.1, 15.1. MS (ESI): m/z calcd for $C_{28}H_{39}N_7O_8Na$ $[M+Na]^+$: 624.2758; found: 624.2757.

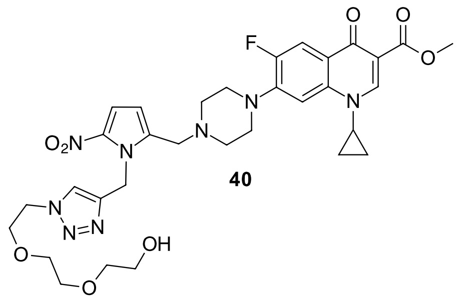

40

*Compound **40.*** **NPYM-34** (24 mg, 0.05 mmol) and azide **37** (9 mg, 0.05 mmol) were dissolved in dry DMF (3 mL) in a round-bottom flask under magnetic stirring and Ar atmosphere. The solution was degassed with three cycles of argon/vacuum. To this solution, a freshly prepared aqueous mixture (1.5 mL) of $Cu(OAc)_2$ (3 mg, 0.014 mmol) and sodium ascorbate (6 mg, 0.028 mmol), previously degassed, was added dropwise. The reaction mixture was degassed and left to stir under argon at room temperature for 16 h. The solvent was evaporated and the crude reaction mixture was purified by silica gel flash chromatography (CH_2Cl_2:MeOH/9.5:0.5) to provide compound **40** (26 mg, 82% yield) as a pale yellow oil. ^1H NMR (600 MHz, DMSO): δ 8.45 (s, 1H), 8.13 (s, 1H), 8.02 (s, 1H), 7.76 (d, J = 13.2 Hz, 1H), 7.44 (d, J = 6.9 Hz, 1H), 6.66 (s, 1H), 5.46 (s, 2H), 4.53 (t, J = 5.0 Hz, 2H), 3.81 (t, J = 5.2 Hz, 2H), 3.74 (s, 3H), 3.68 – 3.61 (m, 1H), 3.63 – 3.55 (m, J = 21.0 Hz, 2H), 3.51 – 3.41 (m, 10H), 3.23 (s, 4H), 2.53 – 2.48 (m, 4H), 1.31 – 1.23 (m, 4H). ^{13}C NMR (150 MHz, DMSO): δ 165.4, 148.8, 143.0, 138.5, 135.1, 128.5, 124.7, 109.5, 72.8, 72.8, 70.3, 70.1, 69.9, 69.1, 60.7, 60.6,

60.2, 51.7, 50.0, 42.8, 35.2, 22.9, 22.2, 21.2, 14.6, 8.0. MS (ESI): m/z calcd for $C_{32}H_{39}FN_8O_8Na$ $[M+Na]^+$: 705.2773; found: 705.2772.

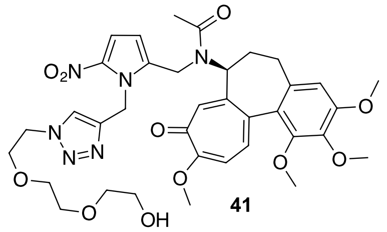

41

Compound ***41.*** **NPYM-35** (11 mg, 0.02 mmol) and azide **37** (4 mg, 0.02 mmol) were dissolved in dry DMF (2 mL) in a round-bottom flask under magnetic stirring and Ar atmosphere. The solution was degassed with three cycles of argon/vacuum. To this solution, a freshly prepared aqueous mixture (1 mL) of $Cu(OAc)_2$ (1.5 mg, 0.007 mmol) and sodium ascorbate (3 mg, 0.014 mmol), previously degassed les, was added dropwise. The reaction mixture was degassed and left to stir under argon at room temperature for 16 h. The solvent was evaporated and the crude reaction mixture was purified by silica gel flash chromatography (CH_2Cl_2:MeOH/9:1) to provide compound **41** (9 mg, 64% yield) as a pale yellow oil. ^1H NMR (400 MHz, MeOD): δ 8.06 (s, 1H), 7.43 (d, J = 10.9 Hz, 1H), 7.30 (s, 1H), 7.24 – 7.16 (m, 2H), 6.67 (s, 1H), 6.03 (d, J = 4.3 Hz, 1H), 4.59 – 4.47 (m, 4H), 4.00 (s, 2H), 3.90 – 3.78 (m, 5H), 3.76 – 3.70 (m, 1H), 3.58 – 3.51 (m, 15H), 3.51 – 3.46 (m, 2H), 2.57 (dd, J = 12.9, 6.8 Hz, 1H), 2.40 – 2.23 (m, 2H), 2.13 (s, 3H), 2.09 – 1.99 (m, 1H). ^{13}C NMR (150 MHz, CD_3OD): δ 180.0, 179.5, 173.5, 164.1, 153.8, 153.1, 150.8, 141.4, 139.0, 137.5, 137.2, 136.5, 134.3, 125.5, 124.1, 114.2, 113.8, 107.3, 80.2, 72.2, 70.0, 68.9, 60.7, 60.2, 55.6, 55.2, 49.9, 44.1, 40.6, 32.5, 29.3, 29.2, 29.0, 20.8. MS (ESI): m/z calcd for $C_{36}H_{44}N_6O_{11}Na$ $[M+Na]^+$: 759.2966; found: 759.2964.

HPLC-MS Analysis and release studies

Chromatographic analyses were performed at flow rate of 0.6 mL/min, injection volume of 5 µL, operating with a gradient elution of A: water (H_2O) and B: acetonitrile (ACN). UV detection was monitored at 254 nm. The analysis started with 5% of B, then B was increased to 95% (from t = 0 to t = 10 min), then kept at 95% (from t = 10 to t = 15 min) and finally return to 5% of eluent B in 1.0 min.

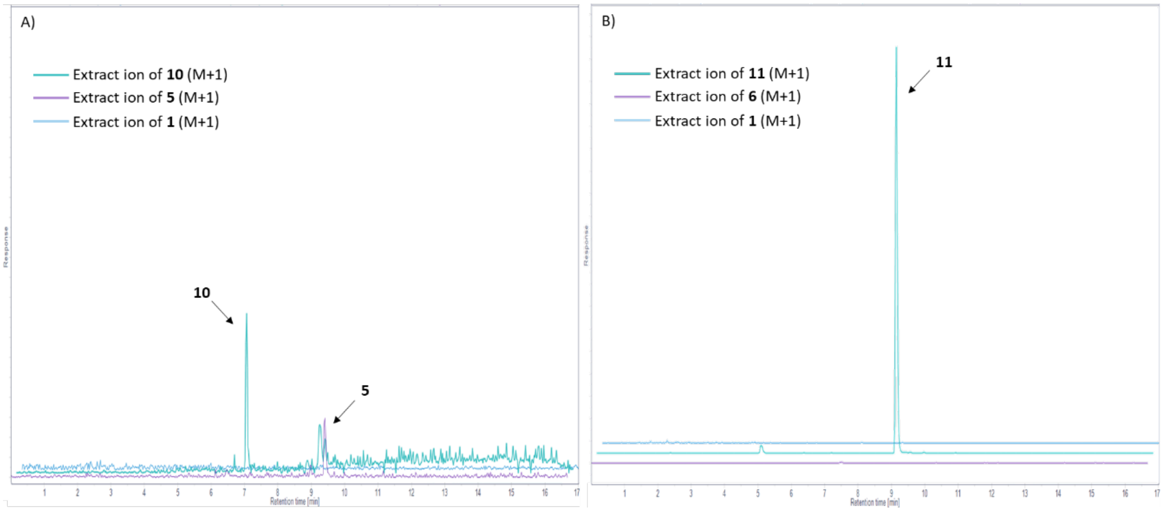

Figure S1: HPLC-MS extract ions chromatograms registered after 2 h of the reduction catalyses by NPs of compounds **5** and **6**. A) chromatograms that show the pick corresponding to amine **10** (green), the disappearance of nitro compound **5** (violet) and the absence of 1-Octanthiol **1** (light blue). B) chromatograms that show the pick corresponding to amine **11** (green), the disappearance of nitro compound **6** (violet) and the absence of 1-Octanthiol **1** (light blue).

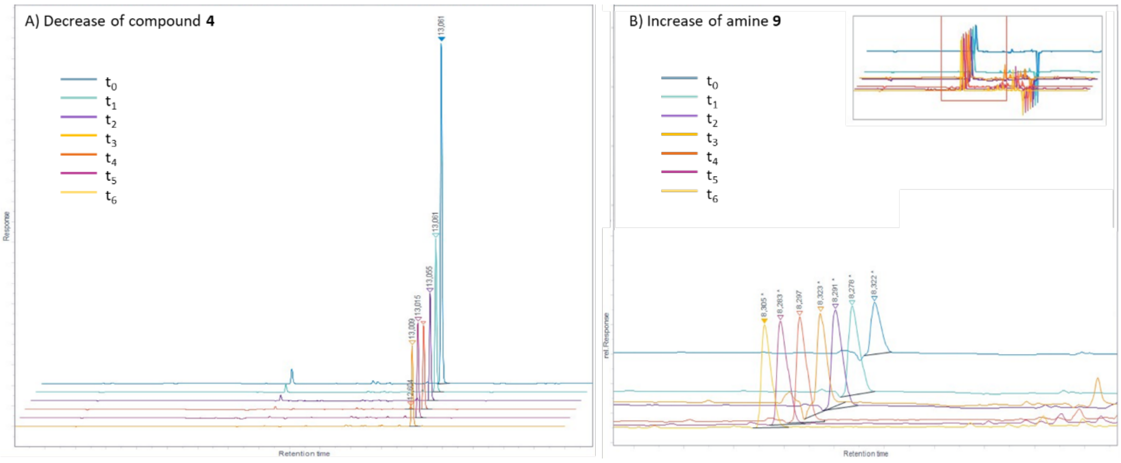

Figure S2: HPLC-MS extract ion chromatograms of the reduction catalyses by NPs of imidazole derivative **4** at set time point. A) Decrease of the nitro compound **4** during the reduction. B) Increase of the amine **9**.

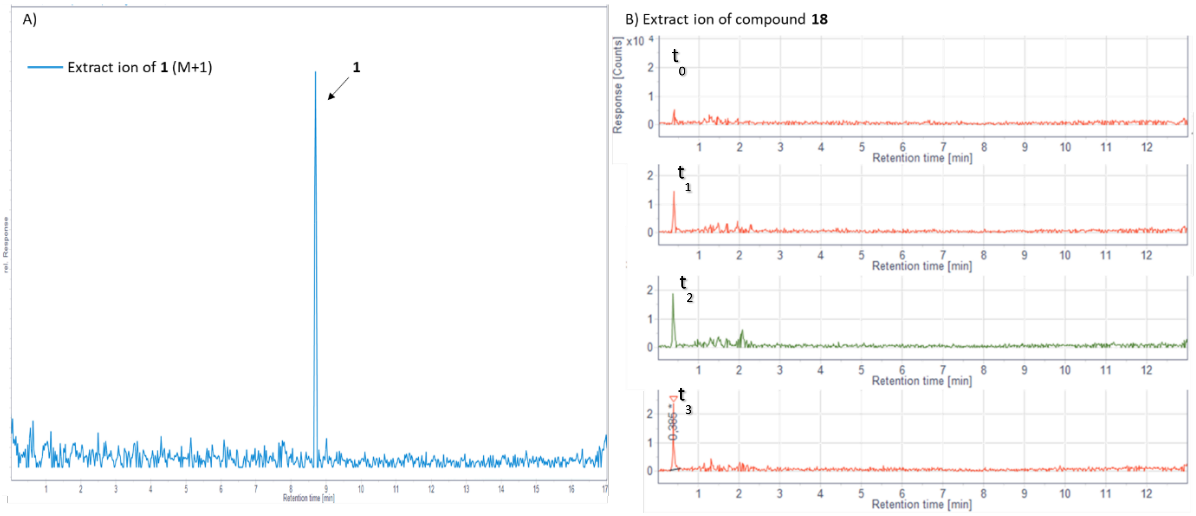

Figure S3: Extract-ion chromatograms of the compound **18** ([MS+1] = 133, R. t. = 0.385) for the reduction of **NPYM-1** with Fe/Pd nanoparticles for the release of 1-octanthiol **1**. The chromatograms were recorded at different time (t_0 = 0 h, t_1 = 0.25 h, t_2 = 0.5 h, t_3 = 1 h).

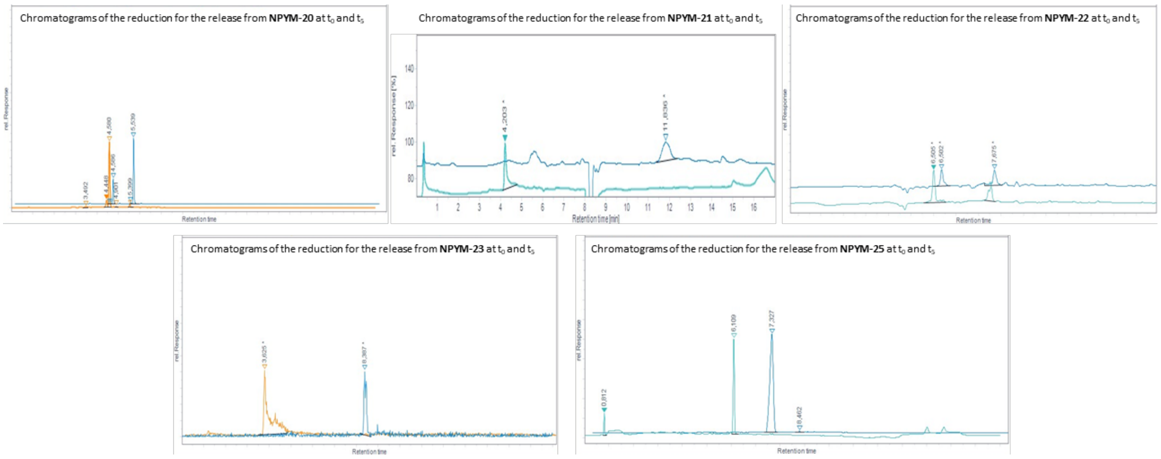

Figure S4: t_0 and t_5 chromatograms for the release of thiols, phenols, sulphonamide, and amines.

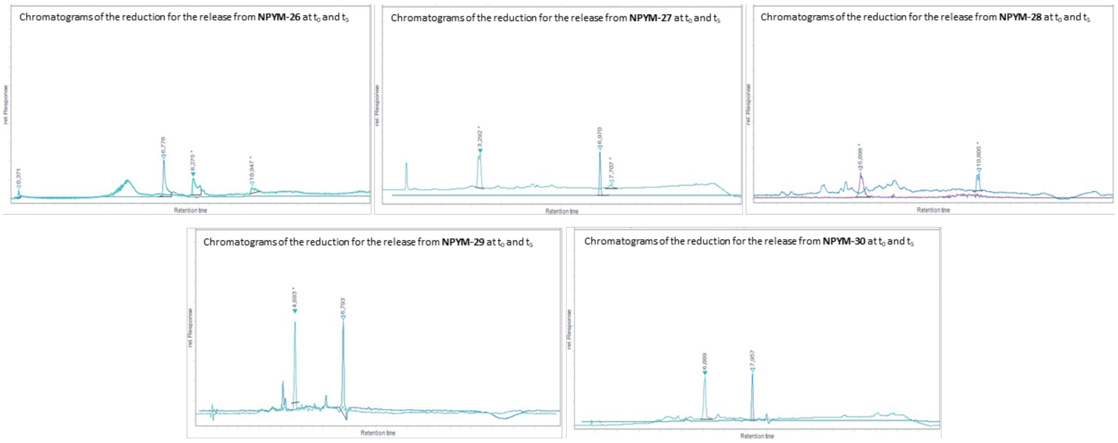

Figure S5: t_0 and t_5 chromatograms for the release of amides

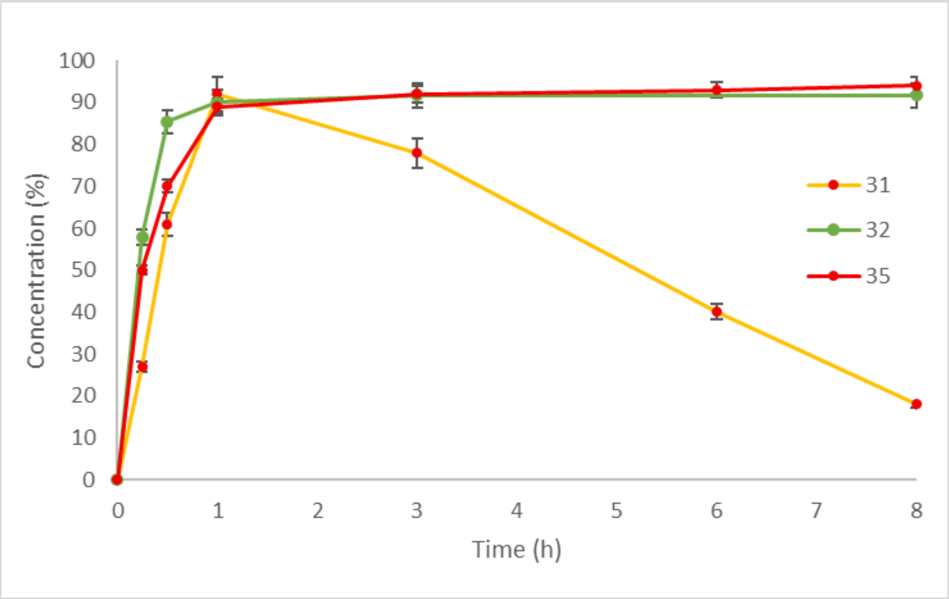

Figure S6: Release profiles of compounds **31-35** from the corresponding NPYM adducts. Compound **31** decrease contemporary to formation of the corresponding disulphide.

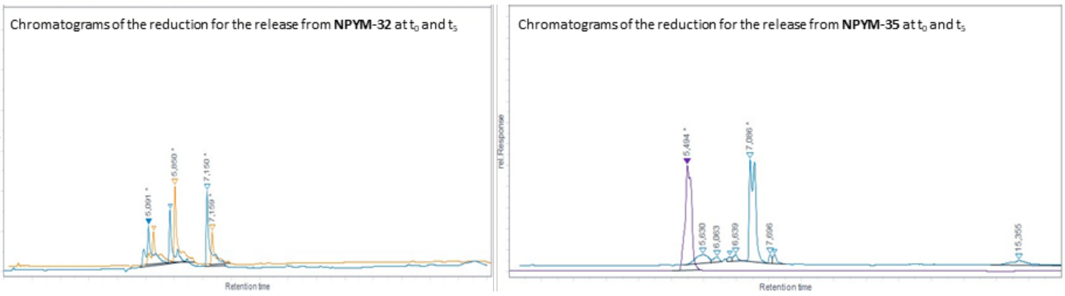

Figure S7: t_0 and t_5 chromatograms for the release of **32** and **35**

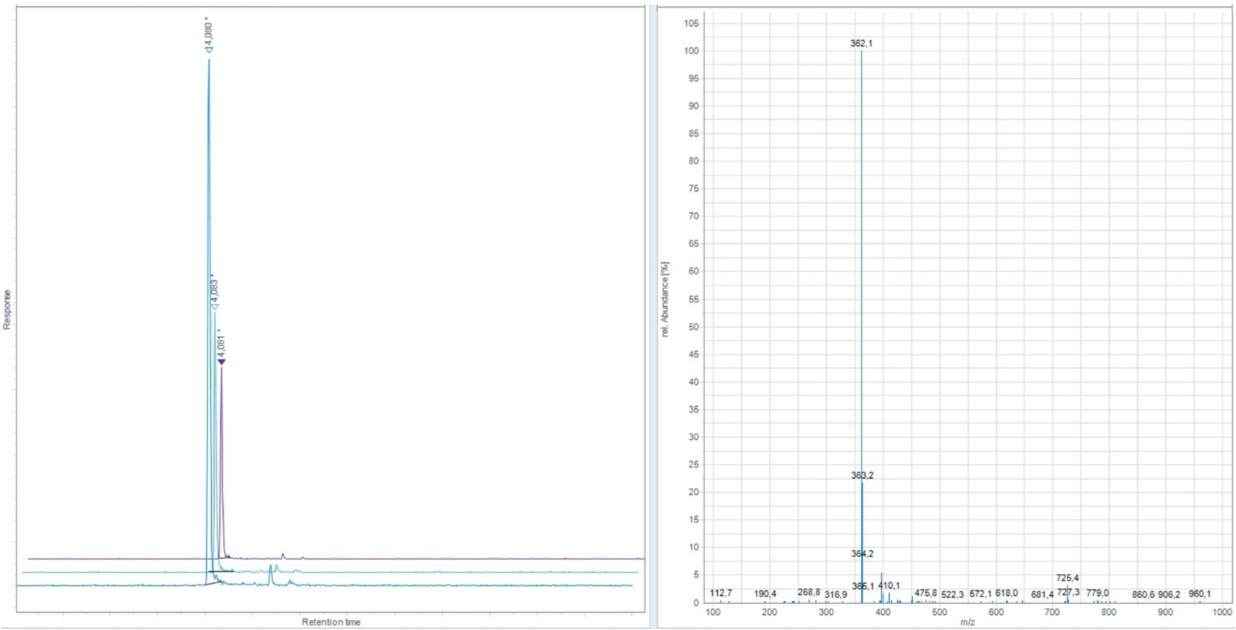

Figure S8: HPLC-MS cromatograms for costructing the calibration curve of active thiol **31**.

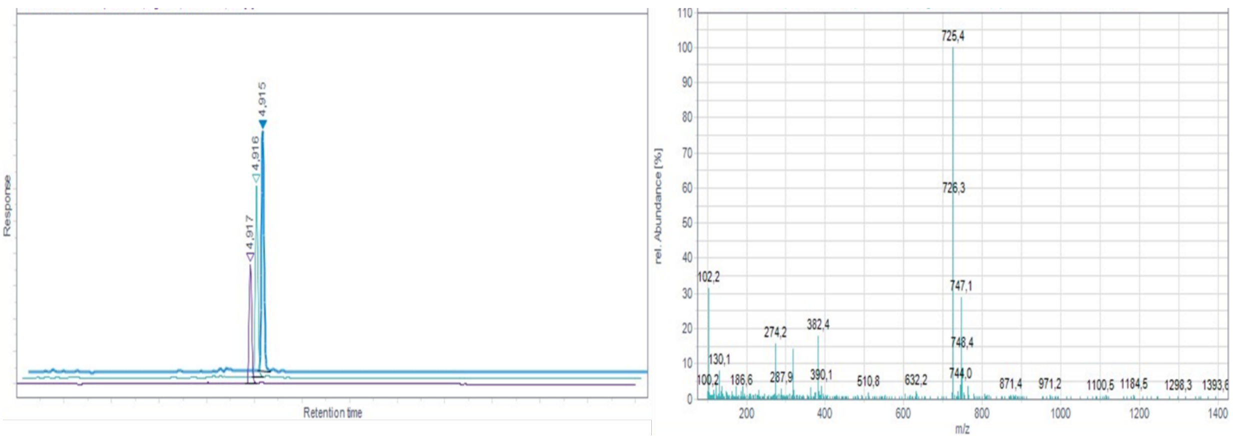

Figure S9: HPLC-MS cromatograms for costructing the calibration curve of disulfide corresponding to the thiol **31**.

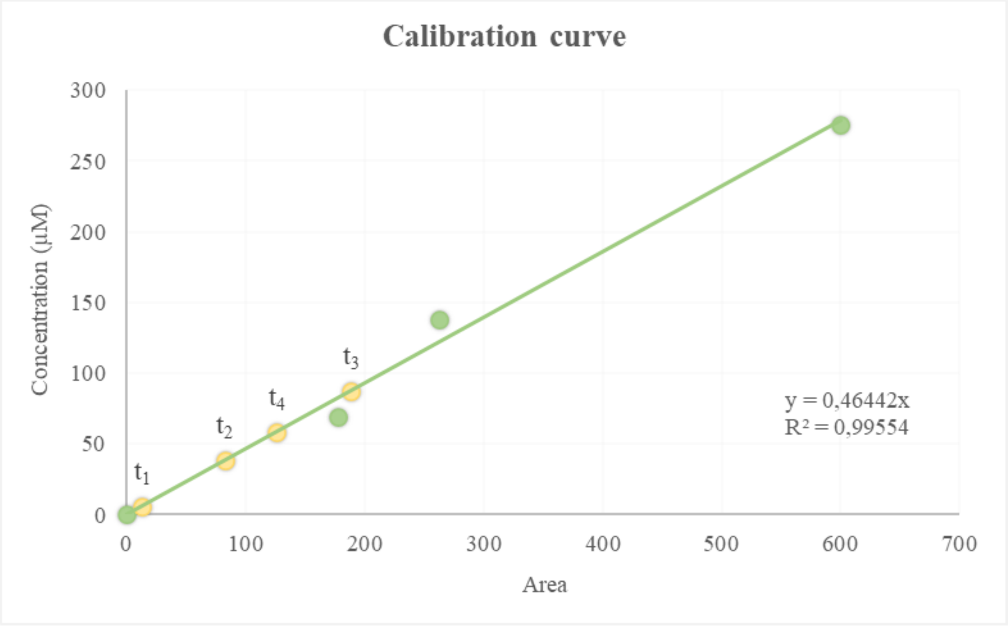

Figure S10: Calibration curve (green line and points, R^2 = 0.99554) for the compound **31** and its concentration monitored at different time during the reduction with nanoparticles (yellow points).

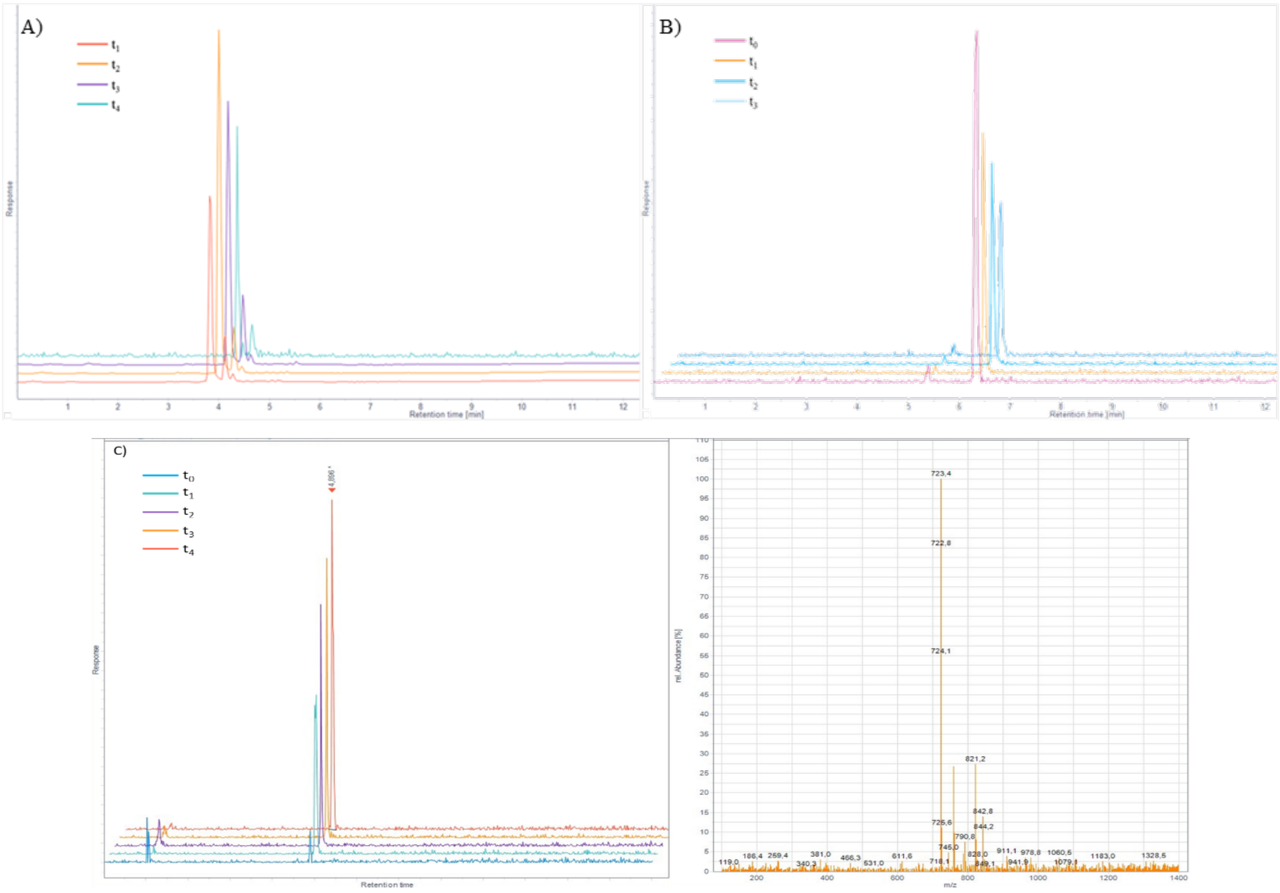

Figure S11: HPLC-MS extract ions chromatograms of the reduction catalyses by NPs of prodrug **31**. A) spectra of **31** at different time showing the increase of the active thiol. Red t_1 (0.25 h), orange t_2 (0.5 h), violet t_3 (1 h) and green t_4 (2 h). B) spectra of **NPYM-31** at different time showing the decrease of the starting compound. Violet t_0 (0 h), orange t_1 (0.25 h), blue t_2 (0.5 h) and light blue t_3 (1 h). C) Chromatograms of the increase of disulfide derivative of thiol **31** at different set time.

Stability in H_2O and PBS

Chemicals: all solvents and reagents were purchased from Sigma-Aldrich Srl (Milan. Italy). Milli-Q quality water (Millipore. Milford. MA. USA), acetonitrile (ACN) and formic acid (FA) were used for the chromatographic analyses.

UV/LC-MS method: for the quantitative analysis was used a UV/LC-MS system. LC analysis was performed by Agilent 1100 LC/MSD VL system (G1946C) (Agilent Technologies. Palo Alto. CA) constituted by a vacuum solvent degassing unit, a binary high-pressure gradient pump, an 1100 series UV detector, and an 1100 MSD model VL benchtop mass spectrometer. The Agilent 1100 series mass spectra detection (MSD) single-quadrupole instrument was equipped with the orthogonal spray API-ES (Agilent Technologies. Palo Alto. CA). Nitrogen was used as nebulizing and drying gas. Chromatographic separation was performed using a Phenomenex Kinetex EVO C18-100Å (150 x 4.6 mm, 5 µm particle size) at room temperature and gradient elution with a binary solution; eluent A was H_2O, while eluent B consisted of ACN (both eluents were acidified with FA 0.1%$_{v/v}$). The analysis started with 5% of B for 3 minute, then rapidly increased up to 95% of B in 10 minutes remaining until 19 minutes; finally, in one minute came back to the initial conditions of 95% of A. The analysis was performed at a flow rate of 0.6 mL/min. UV detection was monitored at 254 nm. Spectra were acquired over the scan range *m/z* 100-1000 both in positive and negative mode.

H_2O and PBS stability: DMSO-stock compounds' solutions were diluted at room temperature (RT) with H_2O or PBS (25 mM, pH 7.4) up to a final concentration of 200 µM. Aliquot samples (50 µL) were taken at fixed time points (time 0 and 24h) and by UV/LC-MS to monitor the amount of unmodified compound.

Discussion: Compounds when incubated for 24 hours in presence of H_2O or PBS (25 mM, pH 7.4) showed high percentages of stability. No significant modifications were appreciated by UV/LC-MS method. Data obtained led us to assume that both **39** and **41** resulted in a stability major than 99.9 % after 24 hours.

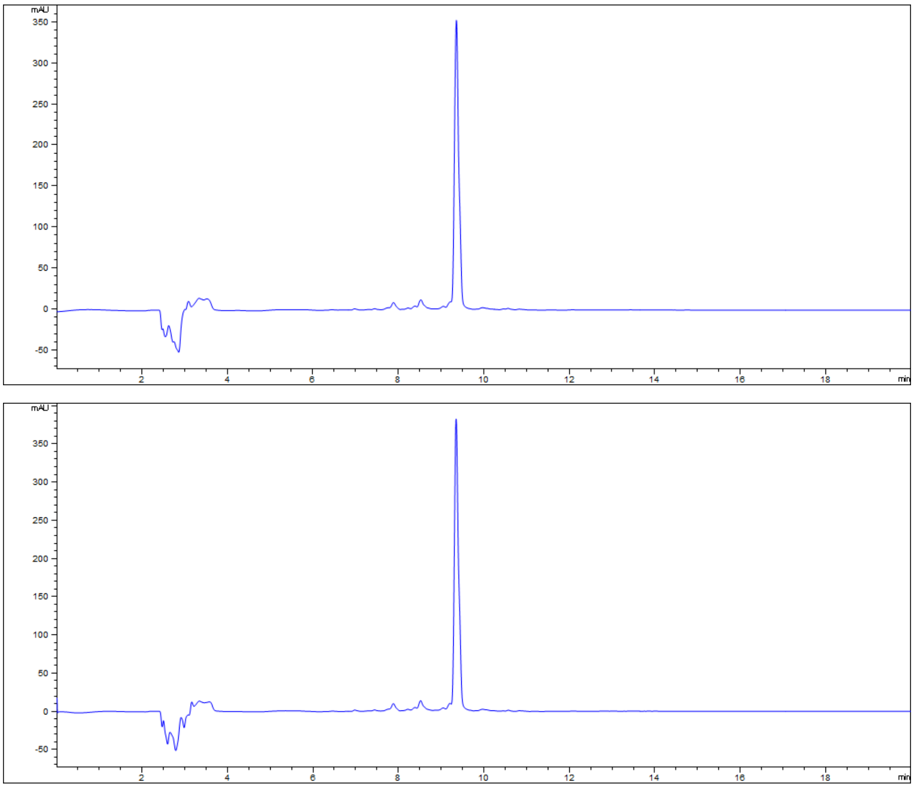

Figure S12: Compound **39** in H_2O at t_0 and 24h

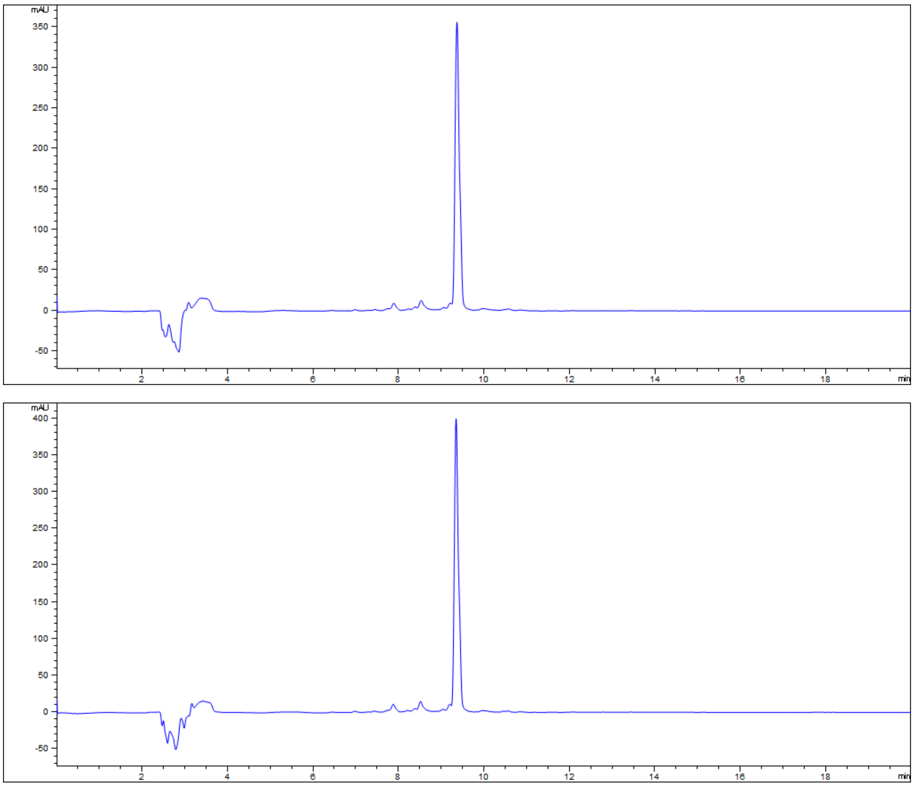

Figure S13: Compound **39** in PBS at t_0 and 24h

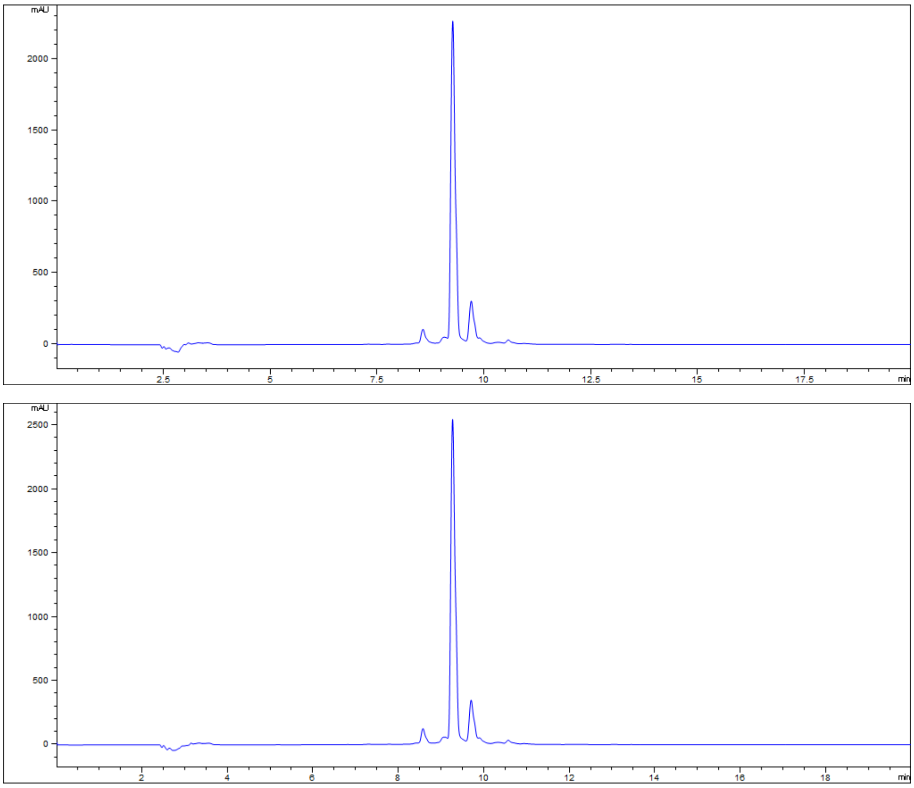

Figure S14: Compound **41** in H_2O at t_0 and 24h

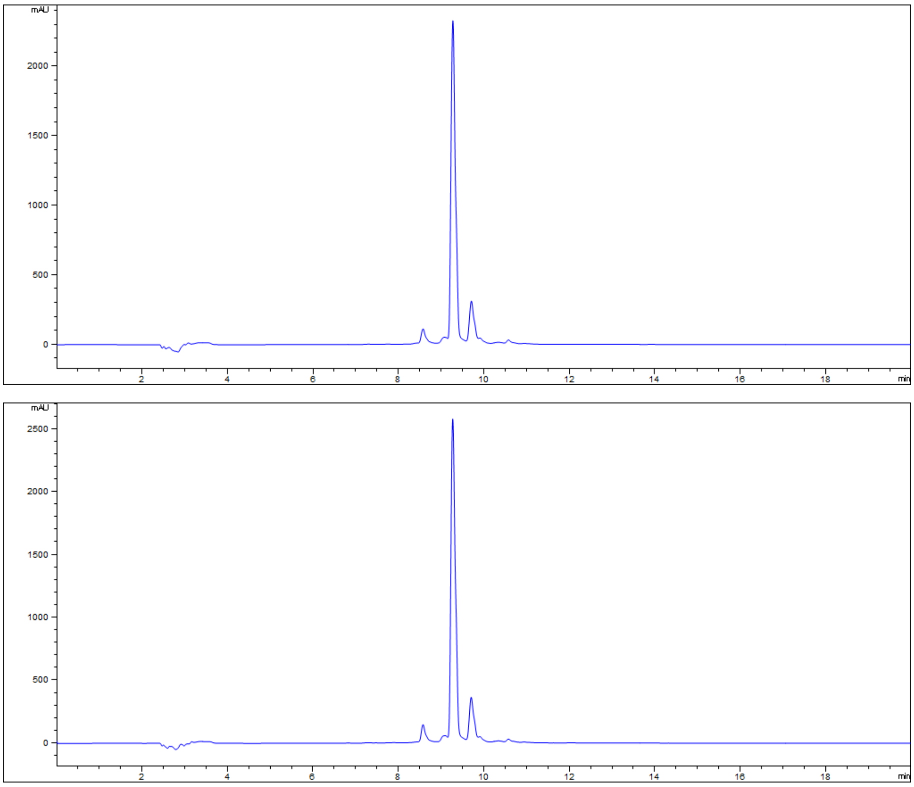

Figure S15: Compound **41** in PBS at t_0 and 24h

Stability in Human Plasma

Pooled human plasma (0.9 mL, 55.7 µg protein/mL) purchased from Sigma-Merck, hepes buffer (1.0 mL, 25 mM, NaCl 140 mM, pH 7.4) and tested compound dissolved in DMSO (100 µL, 2.0 mM) were mixed in a test tube that was incubated at 37 °C under continuous mechanical agitation. At set time points (0.0, 0.25, 0.50, 1.0, 3.0, 5.0, 8.0, and 24.0 h), samples of 100 µL were taken, mixed with 400 µL of cold acetonitrile and centrifuged at 5000 rpm for 15 min. The supernatant was collected and analysed by UV/LC-MS to monitor the amount of unmodified compound. For each compound, the determination was performed in three independent experiments.

UV/LC-MS methods: LC analyses of plasma stability tests were performed by using Agilent 1100 LC/MSD VL system (G1946C) (Agilent Technologies, Palo Alto, CA) constituted by a vacuum solvent degassing unit, a binary high-pressure gradient pump, an 1100 series UV detector, and an 1100 MSD model VL benchtop mass spectrometer. MSD single-quadrupole instrument was equipped with the orthogonal spray API-ES (Agilent Technologies, Palo Alto, CA). The pressure of the nebulizing gas and the flow of the drying gas (nitrogen used for both) were set at 40 psi, 9 L/min, respectively. The capillary voltage, the fragmentor voltage, and the vaporization temperature were 3000 V, 10 V, and 350 °C, respectively. MSD was used in the positive and negative ion mode. Spectra were acquired over the scan range m/z 100-2000 using a step size of 0.1. Chromatographic analyses were performed using a Phenomenex Kinetex EVO C18-100Å (150 x 4.6 mm, 5 µm particle size) at room temperature, at flow rate of 0.6 mL/min, and injection volume of 10 µL, operating with a gradient elution of A: water (H2O) and B: acetonitrile (ACN). Both solvents were acidified with 0.1% v/v of formic acid. UV detection was monitored at 254 nm. The analysis started with 0% of B, then B was increased to 80% (from t = 0 to t = 20 min), then kept at 80% (from t = 20 to t = 25 min) and finally return to 0% of eluent B in 5.0 min.

Kinetic Solubility: The aqueous solubility was determined by adding a DMSO stock solution (initial concentration of 10 mM) to MillQ-H_2O in order to obtain a final concentration of 200 µM (final DMSO concentration of 2% $_{v/v}$). The mix solutions were incubated at RT for 3 hrs. The samples were filtered with 0.45-µm PTFE filters (VWR, Radnor, PA, USA) and analyzed with the UV/LC-MS method reported above. The amount of the solubilized compound was quantified using the appropriate calibration curve realized in DMSO.

Plasma Stability: each compound stock solution in DMSO at the final concentration of 2 mM was incubated in presence of human plasma (55.7 µg protein/mL) and HEPES buffer (25 mM, 140 mM NaCl, pH 7.4) at 37°C under shaking. At selected time points (0, 5, 15, 60, 120, 360, 1440 minutes), 50 µL of the mix solution were added with cold ACN and centrifuged at 5000 rpm for 10 min. The supernatant was removed and analyzed by UV/LC-MS to monitor the amount of unmodified compound. Data were calculated with Excel and plotted using GraphPad Prism 8.0 (GraphPad Software Inc., San Diego, CA, USA). The half-life value ($t_{1/2}$) was calculated with the following formula:

$$t_{1/2} = 0.693/b$$

Where b is the slope found in the linear fit of the natural logarithm of the fraction remaining of the parent compound vs. incubation time.

Discussion: all compounds were incubated at a fixed concentration in presence of human plasma at different time points (from time 0 to 1440 minutes). All compounds showed high percentages of plasma stability until 8 h of incubation but moving to 24 h of treatment the derivatives resulted undergo the hydrolytic action of plasma esterases (**Table S1.** [aHalf-life] (h) expressed as the amount of time it takes before half of the drug is hydrolysed/degraded.**Table S1, Figure S16**). In fact, the percentages of the unmodified compound were reduced for all the derivatives. Compound **41** resulted in the most stable of the series, with more than 70% of plasma stability after 24 and a half-life ($t_{1/2}$) major than 48 h.

Lower plasma stability characterized compounds **39** and **38**, with percentages of the unmodified compound close to 50% and $t_{1/2}$ values of 24.31 h and 33.80 h, respectively.

	**Plasma Stability (% ± SD)**		
**Time (min)**	**41**	**39**	**38**
**0’**	100.00 ± 1.40	100.00 ± 0.31	100.00 ± 0.87
**15’**	101.12 ± 0.86	91.97 ± 0.75	99.44 ± 0.76
**60’**	99.88 ± 1.15	98.70 ± 0.56	98.00 ± 0.83
**120’**	99.60 ± 0.65	97.27 ± 0.60	93.74 ± 0.70
**360’**	100.60 ± 0.94	85.31 ± 0.55	94.73 ± 0.80
**1440’**	75.79 ± 0.64	53.37 ± 0.86	59.61 ± 0.55
**$t_{1/2}$[a]**	>48	24.31	33.80

Table S1. [a]Half-life (h) expressed as the amount of time it takes before half of the drug is hydrolysed/degraded.

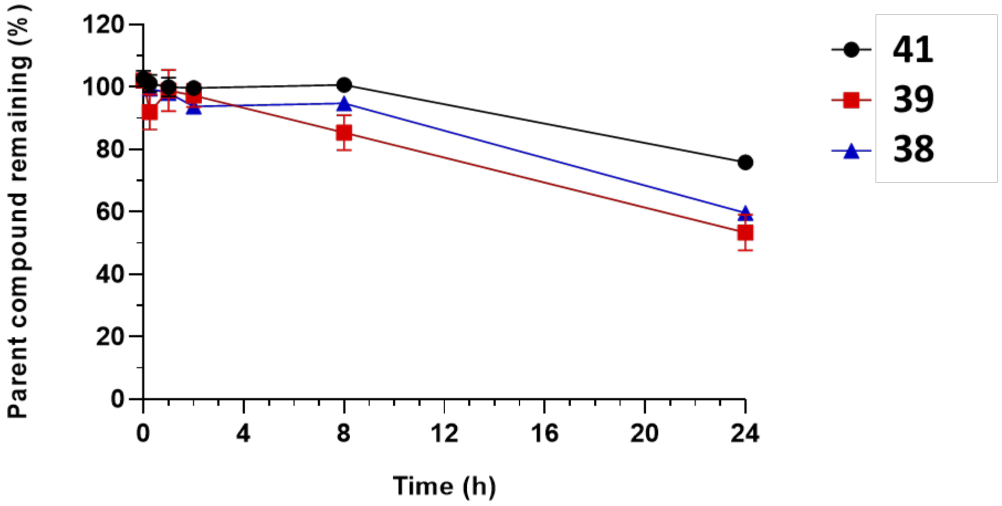

Figure S16: Stability profiles of compounds **41** (black), **39** (red), and **38** (blue) obtained in human plasma. The values the mean +- SD. The experiment runs in triplicate.

Metabolic Stability in human liver microsomes (HLM). In the presence of phosphate buffer (PBS 10 mM, pH 7.4), human liver microsomes (HLM) (0.2 mg/mL), and a NADPH solution in $MgCl_2$ 48 mM. each DMSO compound solution was incubated at 37 °C for 60 minutes under shaking. By adding 1 mL of cold ACN, the metabolizing reactions were stopped. After centrifuging and drying the reaction mixes under an N_2 flow, the quantitative analyses were performed using the UV/LC-MS method previously described. The percentages of the metabolized and unmetabolized compounds were calculated as previously described.[4]

Discussion: Firstly, the aqueous solubility of compounds was predicted by the web tool SwissADME commonly used to evaluate drug-likeness, ADME, and PK properties of small molecules. The predicted logarithm of Solubility (LogS) values was -3.53 (**Table S2**). In support of the above data, the kinetic solubility was assessed for compound **38**. Thus, after incubating a stock solution of **38** in presence of Mill-Q water for 3 hrs, the quantitative analysis conducted with the HPLC-UV-MS method above reported confirmed that this derivative may be classified as a soluble compound, with LogS value of -3.95.

	LogS[a] (SwissADME)	LogS[a]	Papp[b]	Metabolic Stability (%)	Metabolite Formation (%)[c]
**38**	-3.53	-3.95	0.15	93.5	M_1 = 6.5

Table S2. *[a]Logarithm of Solubility expressed as mol/L. [b]Apparent permeability (Papp) reported in 10^{-6} cm/sec. [c]M_1 = M + OH (+ 16). n.d. means not determined.*

Compound **38** slightly underwent a phase I metabolism leading to great percentages of metabolic stability (93.5%). The mono-oxidate derivative (M_1 = M + OH) obtained as a probable consequence of the introduction of a -OH group on the aromatic ring, was detected and quantified with the HPLC-UV-MS method above described.

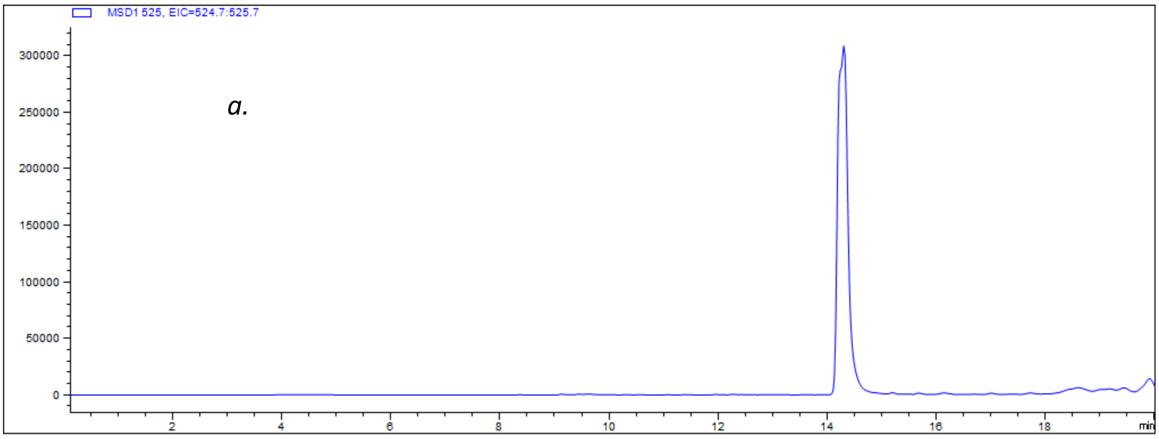

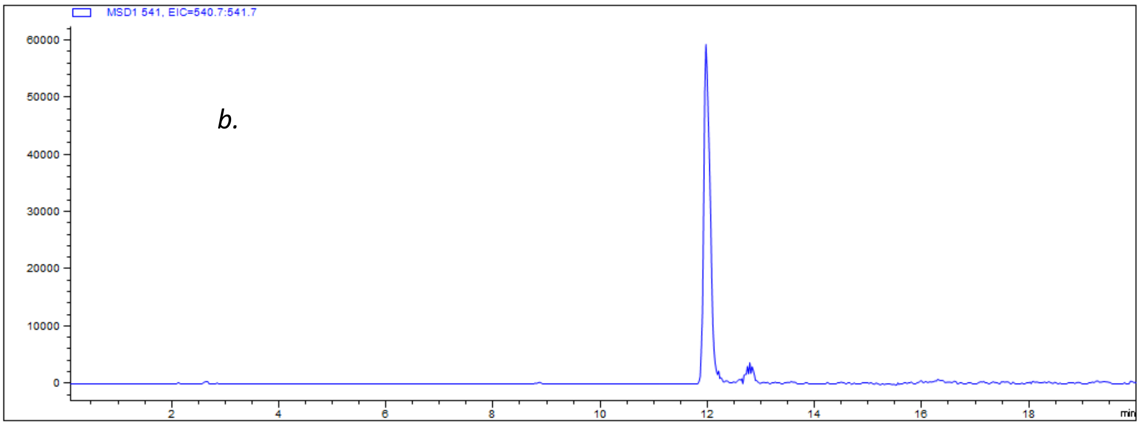

Figure S17: Metabolic Stability *a)* parent compound **38** (M^+ 525); *b)* mono-oxidate metabolite (M_1 = M + OH) (M^+ 541)

Cell culture, MTT assay and Western blotting. A431 epidermoid carcinoma cells and FU human fibroblast (ATCC, Rockville, MD, USA) were cultured in DMEM (Euroclone) supplemented with 10% fetal bovine serum (FBS, Euroclone), 100 U/ml penicillin/streptomycin (Euroclone), and 4 mM L-glutamine (Euroclone). HT29 colorectal adenocarcinoma cells (ATCC, Rockville, MD, USA) were cultured in RPMI-1640 (Euroclone) medium supplemented with 10% fetal bovine serum (FBS, Euroclone), 100 U/ml penicillin/streptomycin (Euroclone), and 4 mM L-glutamine (Euroclone). All cell lines were grown at 37°C and 5% CO_2.

MTT assay. A431 (3.0×10^3 cells/well), HT29 (3.5×10^3 cells/well) and FU (1.5×10^3 cells/well) were plated in 96-well multiplates in medium containing 10% FBS. After adherence, the medium was removed, and cells were incubated for 24h in 0.1% FBS. Then cells were treated with different concentrations of ST7612AA1, compounds **38** and **16** (0.1, 1 and 10 µM), nitroreductase (2 µM) and NADH (100 µM) in presence of 0.1% FBS. When indicated, cells were incubated in hypoxic conditions at 94% N_2, 5% CO_2, 1% O_2 and 37°C for 24 h. After 48 h, the medium was removed, and cells were incubated for 2 h with fresh medium in the presence of 1.2 mM MTT (3-(4,5-dimethylthiazol-2-yl)-2,5-diphenyltetrazolium bromide) (Sigma-Aldrich). The MTT solution was then removed and 50 µL of DMSO were added to each well to dissolve the blue formazan crystals. The absorbance of the formazan dye was measured at 570 nm with a microplate reader (EnVision, PerkinElmer, Waltham, MA, USA). Data were expressed as a percentage of the basal control.

Evaluation of nitroreductase activation. HT29 (1.5×10^5 cells/well) were seeded on a glass bottom dish and incubated overnight in a CO_2 incubator at 37°C. Then cells were treated with Image-iT^{TM} Green Hypoxia Reagent (Invitrogen) at the final concentration of 4 µM and incubated in hypoxic chamber (1% O_2, 5% CO_2 and 37°C) for 24h. Subsequently, the nitroreductase activation through hypoxia was detected by a microscope (Nikon, Eclipse Ts2) with FITCH filter set.

Western Blotting. HT29 (3.5×10^5 cells/well) were seeded in 6-well multiplates in RPMI-1640 added with 10% serum. After adhesion cells were incubated in hypoxic chamber (1% O_2, 5% CO_2 and 37°C) for 24h. Subsequently cells were treated with ST7612AA1 and **38** (10 µM). After 48 h cells were lysated and centrifuged at 15,000×g for 15 min at 4 °C. Protein content was measured using a BCA protein assay kit (Thermo Scientific). For Western blotting analysis, aliquots of cell extract supernatants containing an equal amount of proteins (50 µg) were treated with Laemmli buffer, boiled for 10 min, resolved on 15% stain-free gel and then blotted onto a nitrocellulose membrane. Membranes were incubated with 1:1000 dilution of anti-Acetyl Histone H4 (Cell Signaling) and with 1:400 dilution of anti-β actin (Invitrogen). The membranes were then incubated with 1:3,000 dilutions of horseradish peroxidase-conjugated secondary antibody (Cell Signaling) for 1 h at RT. Chemiluminescence was detected by Image Quant Las 4000 imager (GE Healthcare).

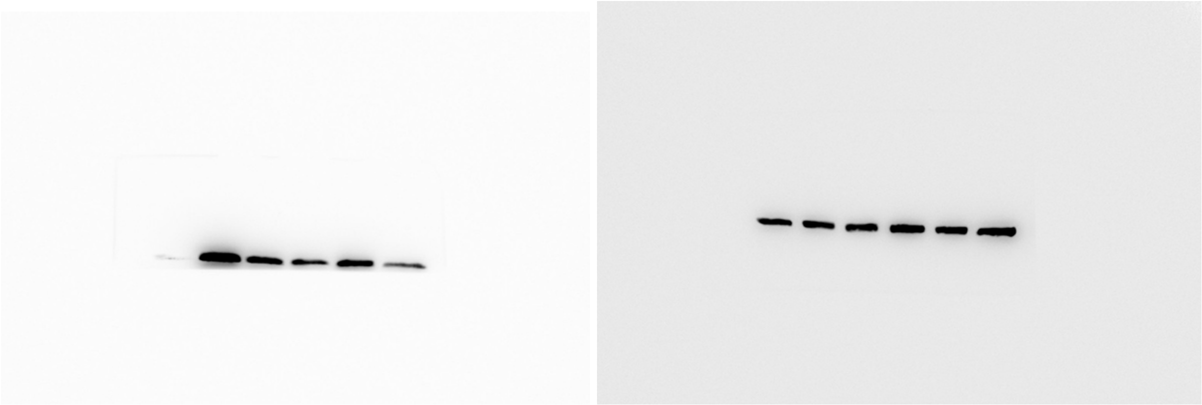

Figure S18. Comparison of the original gel of Figure 4c treated with anti-Acetyl Histone H4 (left) with the treatment with anti β-actin (right) used for normalization. 1 Control, 2 ST7612AA1, 3 compound **38**. Other spots are HDAC inhibitors not related to this publication.

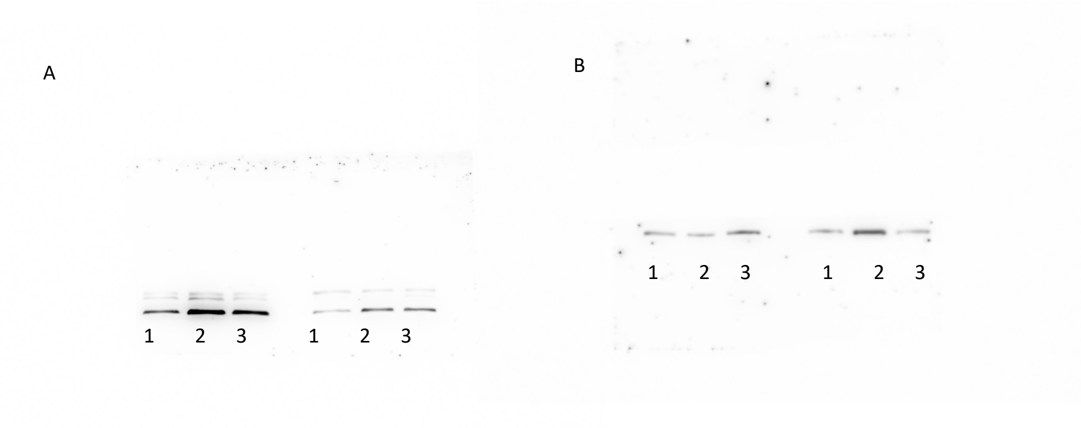

Figure S19. Gel of two independent experiments on the same samples.

Indirect validation of the computational data

The level of $\Delta G^{\ddagger}$ values calculated as described in the main text was compared with the half-life times measured for the three PABA-like linkers **H**, **I** and **J** (Scheme S20) described in ref. 5. The values obtained, reported in Table S2, shows a similar trend with the experimental findings, confirming the value of our computational approach to predict the behavior of different linkers for 1-6 self immolative release.

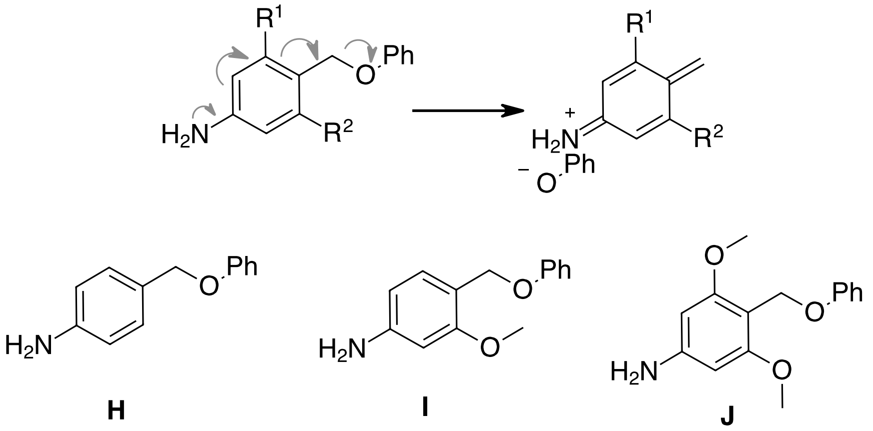

Figure S20. Model reaction and substrates described in ref.5. The only difference in calculation is the constraint on the benzyl C-O distance used in the search of the TS, which was fixed at 2 Å.

Table S2. Activation free energies ($\Delta G^{\ddagger}$) computed from thermochemical analyses of substrates and transitions states obtained at B3LYP-D3BJ/def2-TZVPP (geometry optimisations and frequency calculations, including water through the IEF-PCM solvation model).

Substrate	$\Delta G^{\ddagger}$ (kJ mol^{-1})
H	89.46
I	85.38
J	72.36

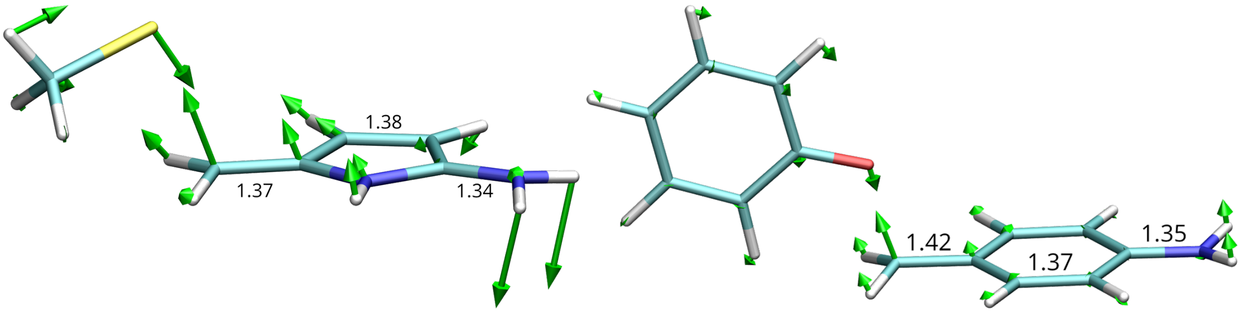

Figure S21. Examples of identified transition states for the reaction of aminopyrrole (left) and aniline (rigth), along with some relevant bond distances (in Å). Green arrows represent atomic displacements of the imaginary mode describing the reaction coordinate, and have been arbitrarily scaled for visualisation purposes.

References

1. J. Feng, S. Handa, F. Gallou, B. H. Lipshutz, *Angew. Chem. Int. Ed*. 2016, **55**, 8979.
2. C. Schmuck, J. Dudaczek, *Tetrahedron Lett.* 2005, **46**, 7101.
3. D-J. Chang, E-Y. Yoon, -GB. Lee, S-O. Kim, W-J. Kim, Y-M. Kim, J-W. Jung, H. An, Y-G. Suh, *Bioorg. Med. Chem. Lett*. 2009***, 19***, 4416.
4. S. Murthy, MG. Nizi, MM. Maksimainen, S. Massari, J. Alaviuhkola, BE. Lippok, C. Vagaggini, ST. Sowa, A. Galera-Prat, Y. Ashok, H. Venkannagari, R. Prunskaite-Hyyryläinen, E. Dreassi, B. Lüscher, P. Korn, O. Tabarrini, L. Lehtiö. *J Med Chem*. 2023, **66**, 1301. A. Brai, V. Riva, F. Saladini, C. Zamperini, CI. Trivisani, A. Garbelli, C. Pennisi, A. Giannini, A. Boccuto, F. Bugli, M. Martini, M. Sanguinetti, M. Zazzi, E. Dreassi, M. Botta, G. Maga, *Eur J Med Chem*. 2020, **200**, 112319.
5. K. M. Schmid, L. Jensen and S. T. Phillips, *J.Org. Chem.*, 2012, **77**, 4363–4374.

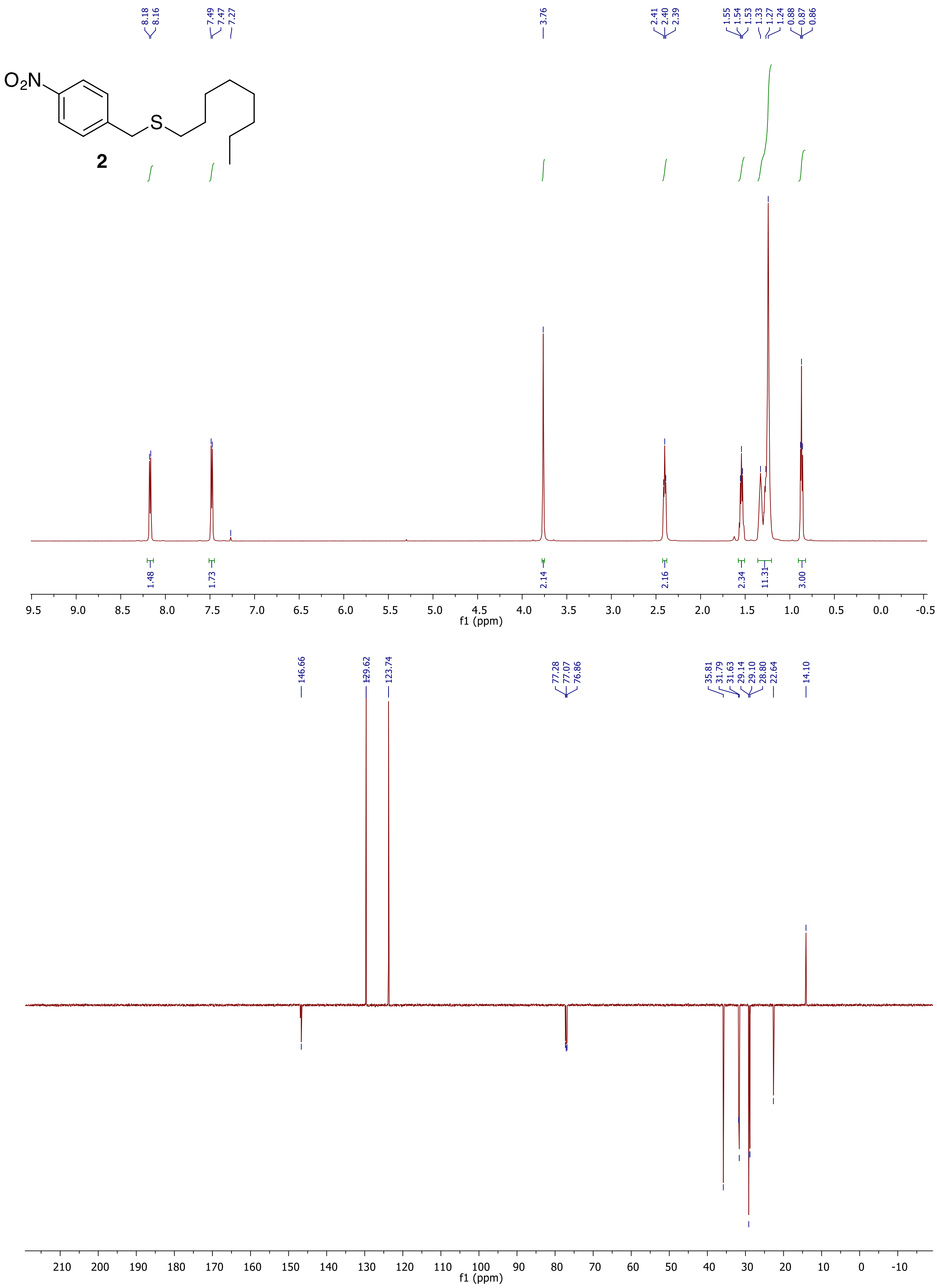
O2N
S
2
8.18
8.16
7.49
7.47
7.27
3.76
2.41
2.40
2.39
1.55
1.54
1.53
1.33
1.27
1.24
0.88
0.87
0.86
1.48
1.73
2.14
2.16
2.34
11.31
3.00
f1 (ppm)
146.66
129.62
123.74
77.28
77.07
76.86
35.81
31.79
31.63
29.14
29.10
28.80
22.64
14.10
f1 (ppm)

3

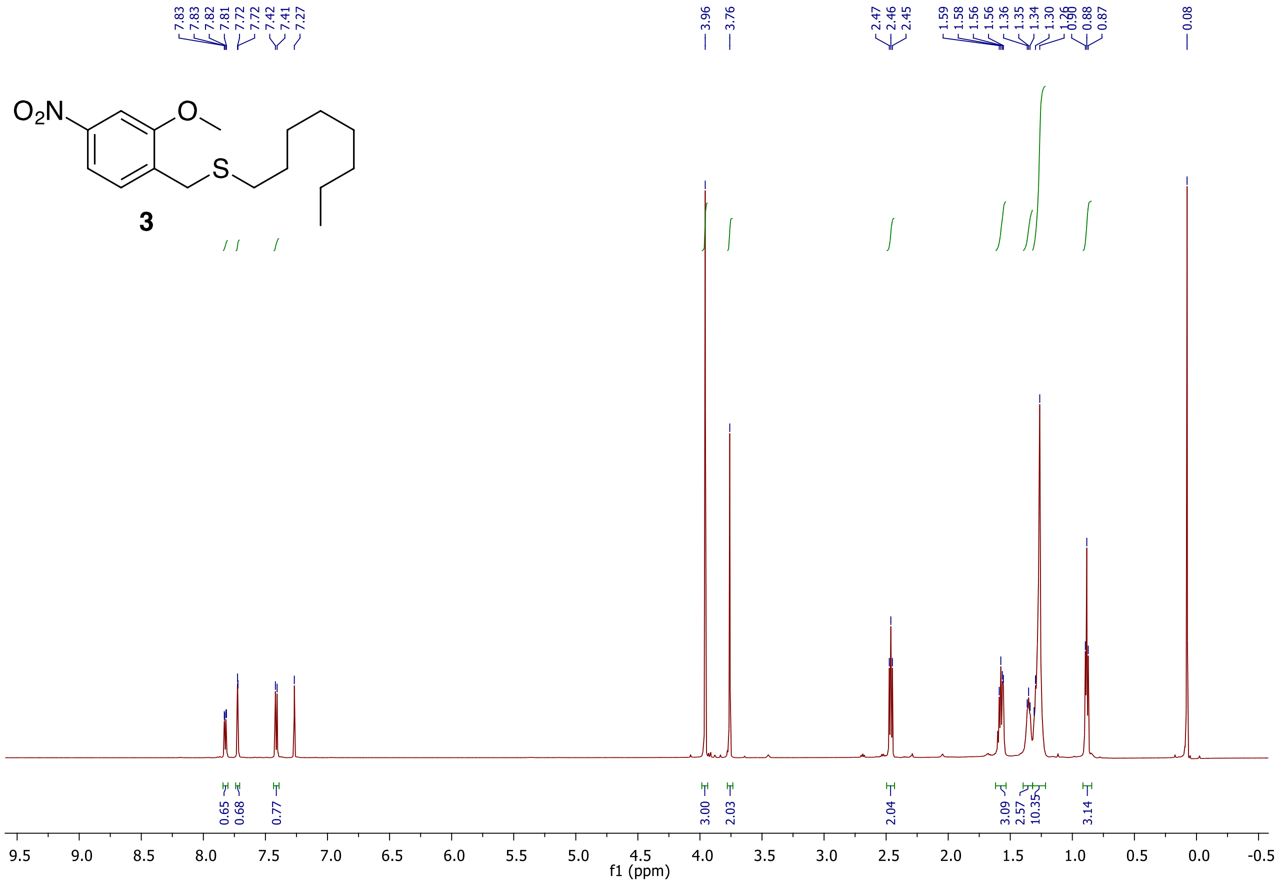
O2N
O
S
7.83
7.83
7.82
7.81
7.72
7.72
7.42
7.41
7.27
3.96
3.76
2.47
2.46
2.45
1.59
1.58
1.56
1.56
1.36
1.35
1.34
1.30
1.26
0.90
0.88
0.87
0.08
0.65
0.68
0.77
3.00
2.03
2.04
3.09
2.57
10.35
3.14
f1 (ppm)

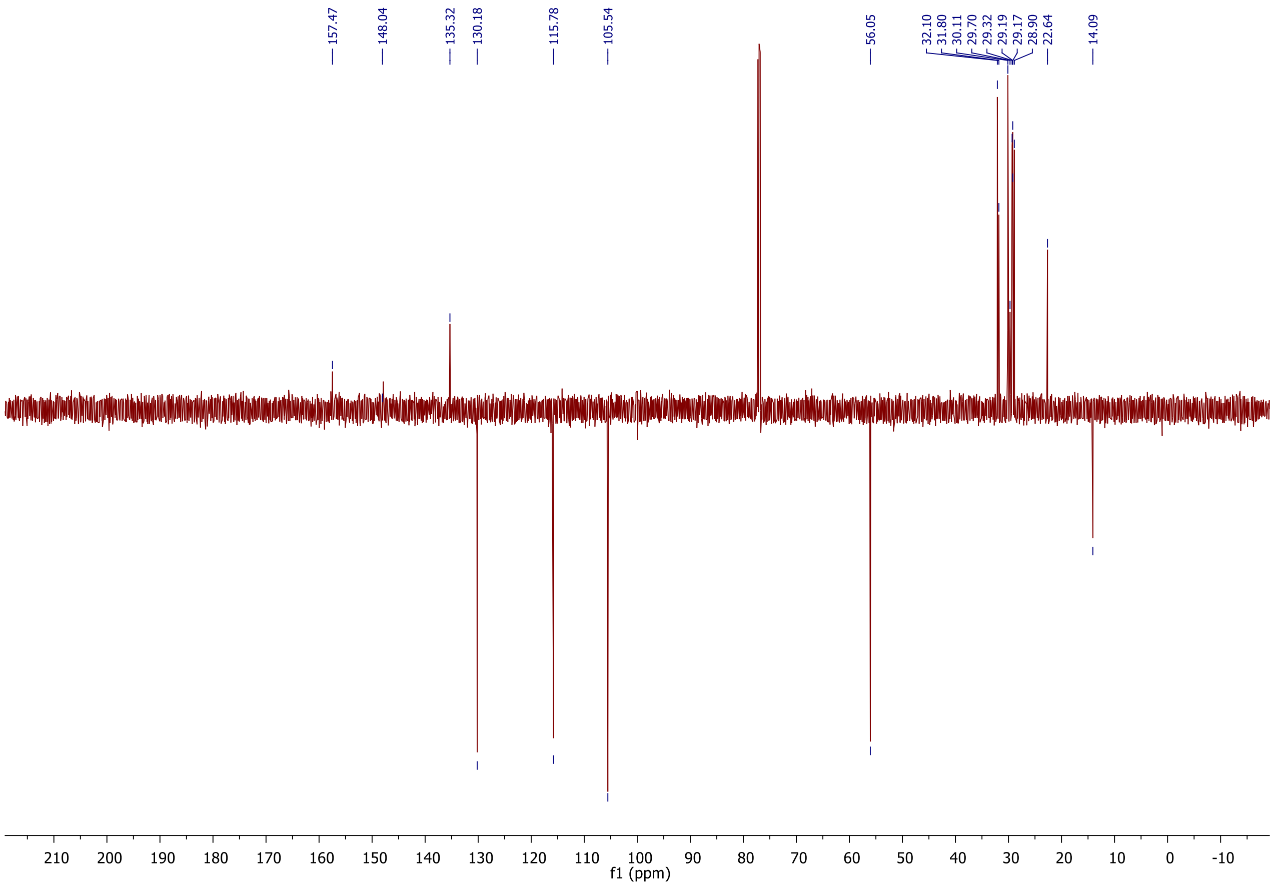
157.47
148.04
135.32
130.18
115.78
105.54
56.05
32.10
31.80
30.11
29.70
29.32
29.19
29.17
28.90
22.64
14.09
f1 (ppm)

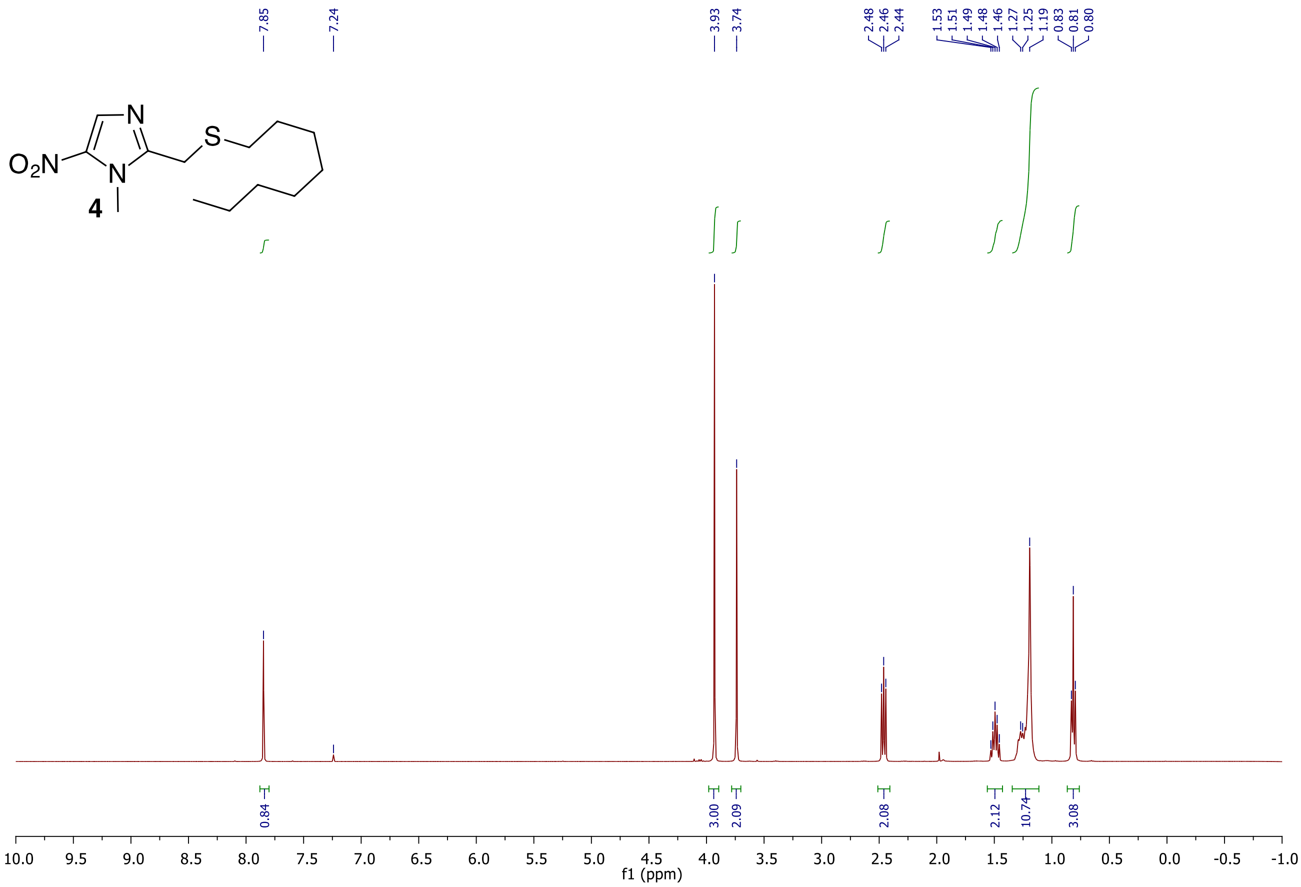

4

7.85
7.24
3.93
3.74
2.48
2.46
2.44
1.53
1.51
1.49
1.48
1.46
1.27
1.25
1.19
0.83
0.81
0.80
0.84
3.00
2.09
2.08
2.12
10.74
3.08
f1 (ppm)

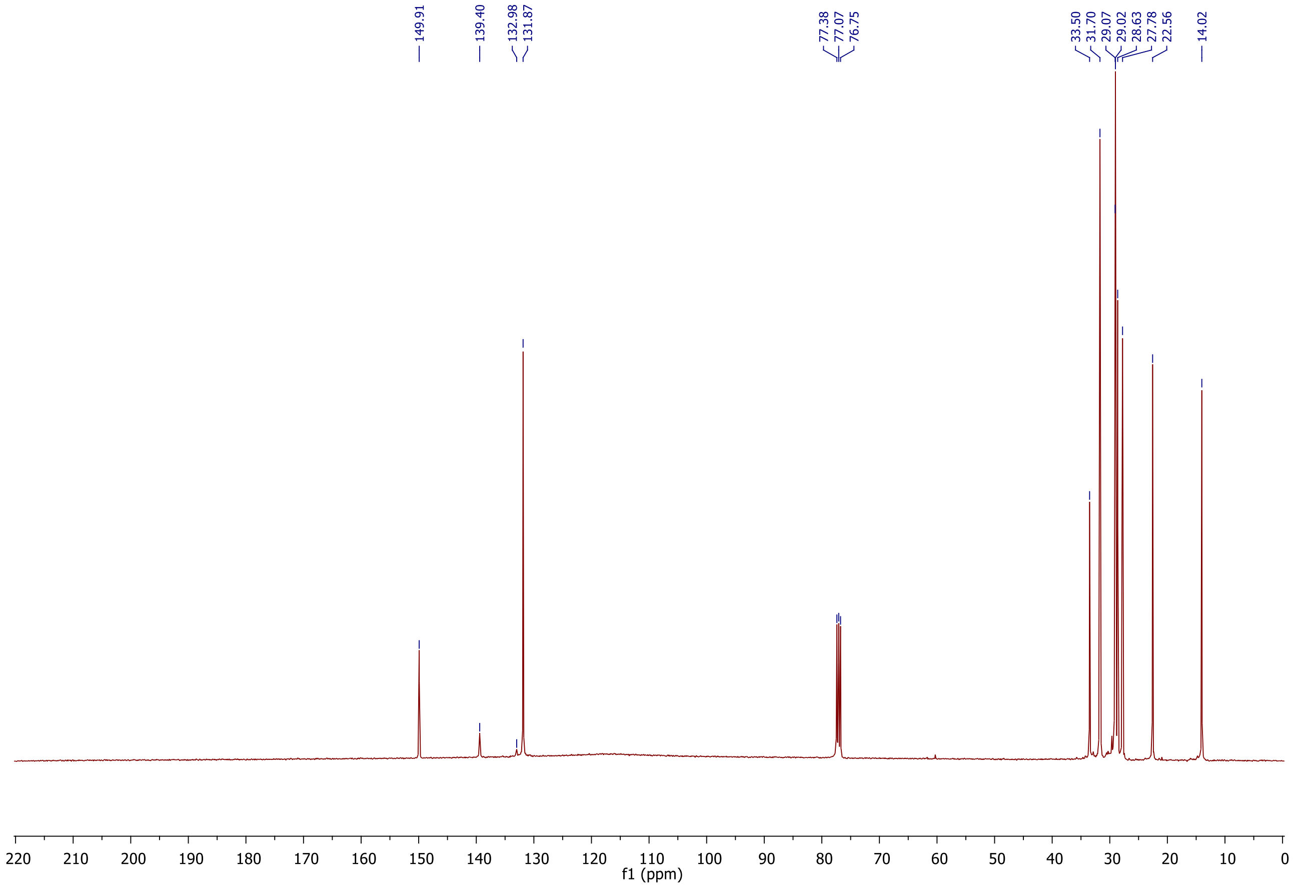

149.91
139.40
132.98
131.87
77.38
77.07
76.75
33.50
31.70
29.07
29.02
28.63
27.78
22.56
14.02
f1 (ppm)

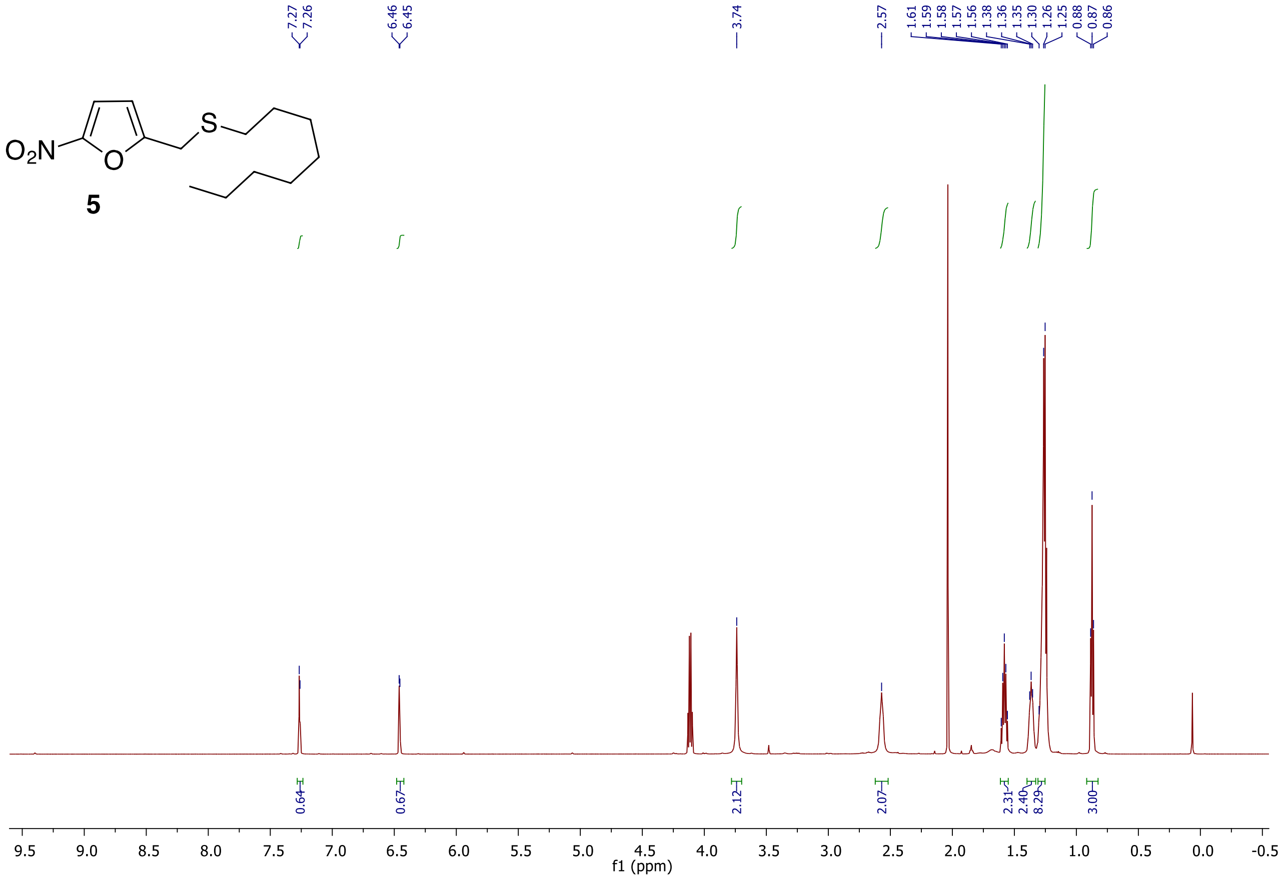

5

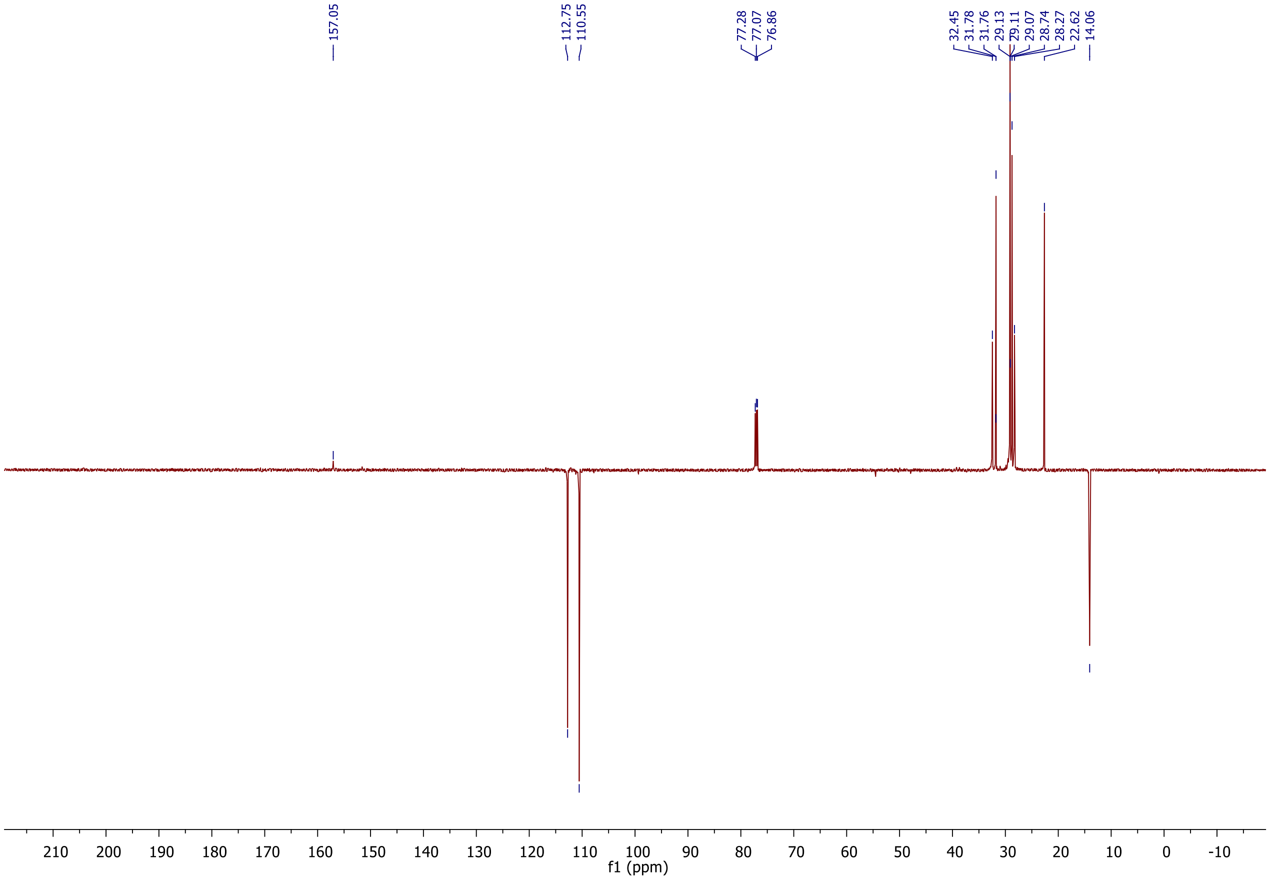

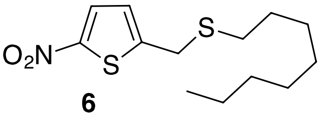
O2N
S
S

6

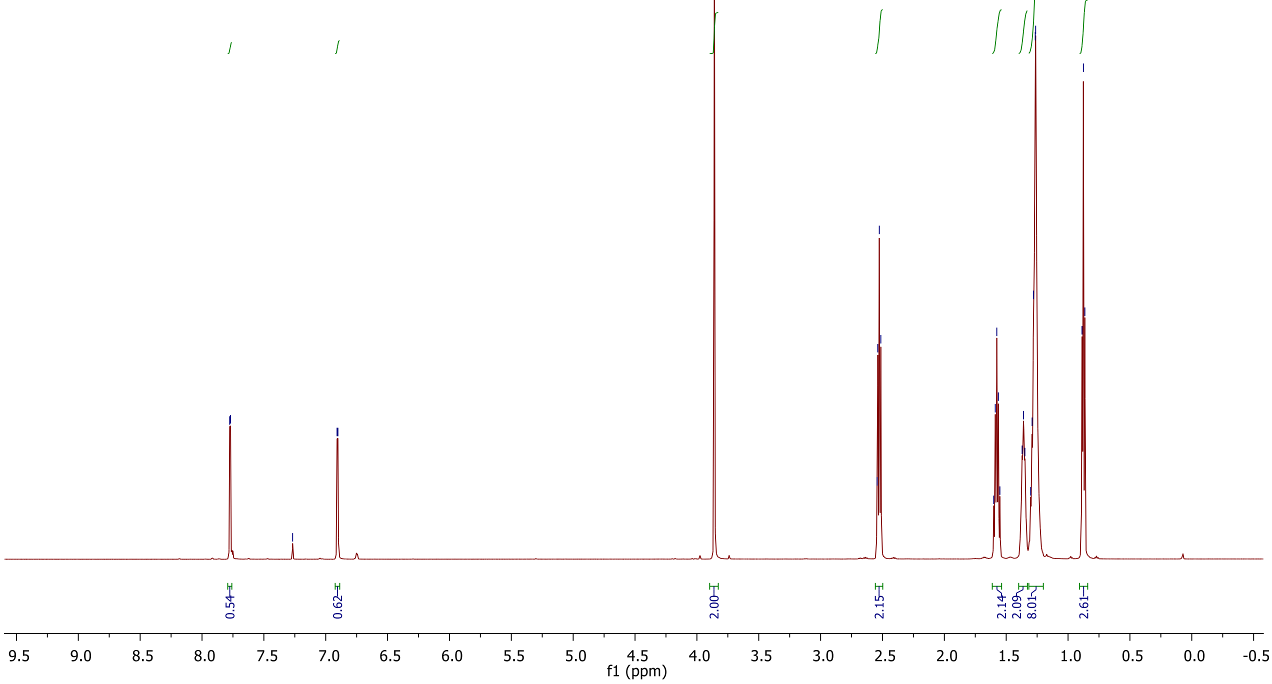
7.78
7.77
7.27
6.91
6.90
3.86
2.54
2.54
2.53
2.51
1.59
1.58
1.56
1.37
1.36
1.35
1.29
1.28
1.27
1.26
0.89
0.88
0.86
0.54
0.62
2.00
2.15
2.14
2.09
8.01
2.61
9.5 9.0 8.5 8.0 7.5 7.0 6.5 6.0 5.5 5.0 4.5 4.0 3.5 3.0 2.5 2.0 1.5 1.0 0.5 0.0 -0.5
f1 (ppm)

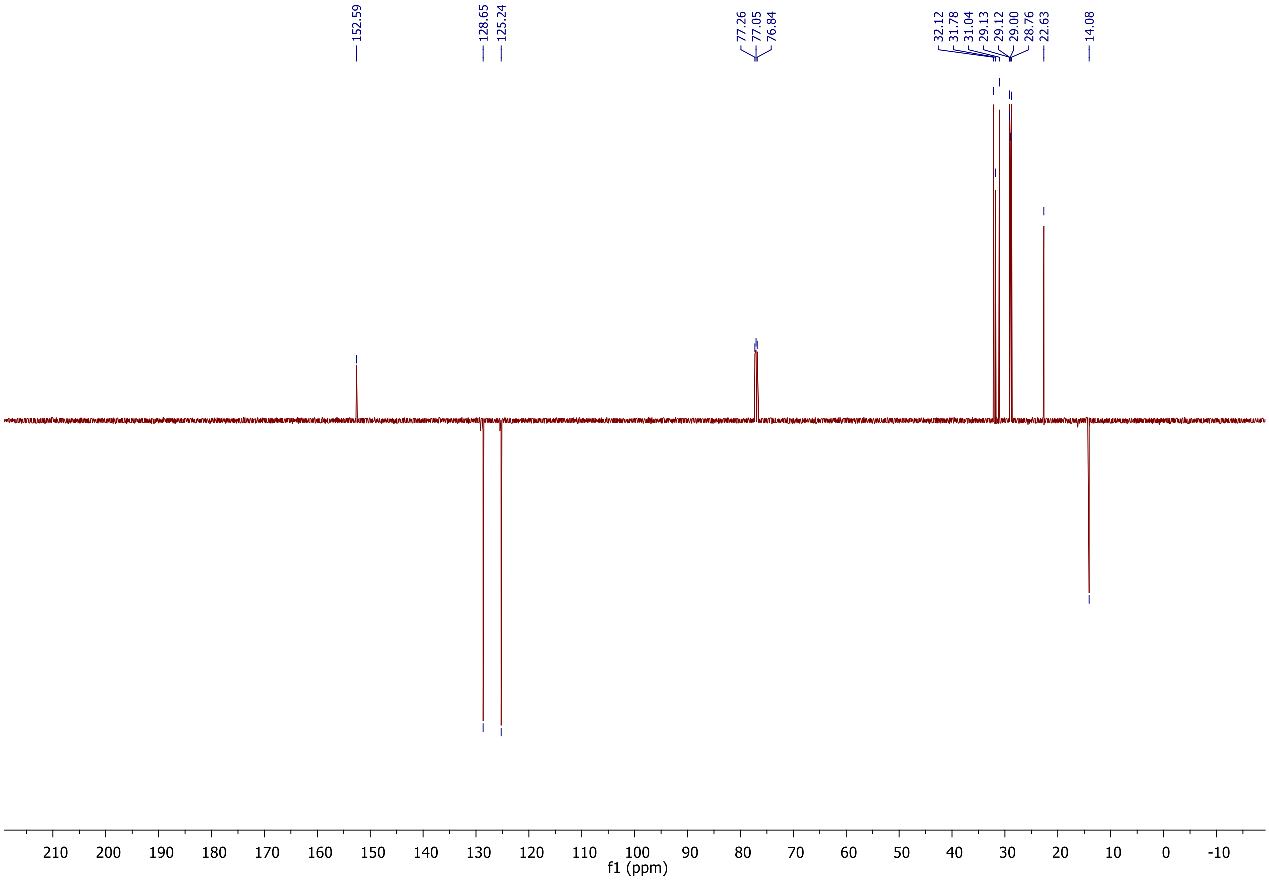
152.59
128.65
125.24
77.26
77.05
76.84
32.12
31.78
31.04
29.13
29.12
29.00
28.76
22.63
14.08
210 200 190 180 170 160 150 140 130 120 110 100 90 80 70 60 50 40 30 20 10 0 -10
f1 (ppm)

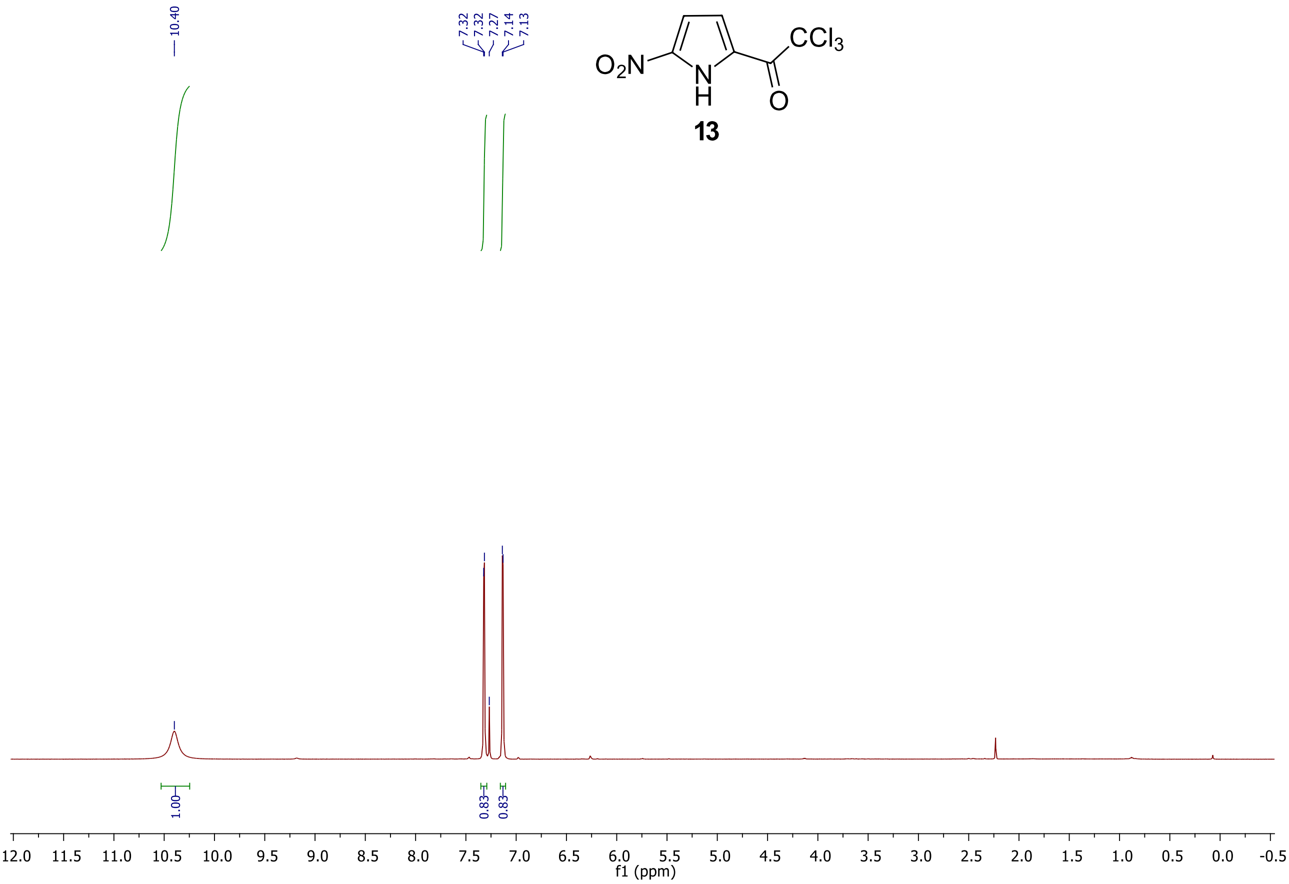

10.40
7.32
7.32
7.27
7.14
7.13
1.00
0.83
0.83
12.0 11.5 11.0 10.5 10.0 9.5 9.0 8.5 8.0 7.5 7.0 6.5 6.0 5.5 5.0 4.5 4.0 3.5 3.0 2.5 2.0 1.5 1.0 0.5 0.0 -0.5
f1 (ppm)

13

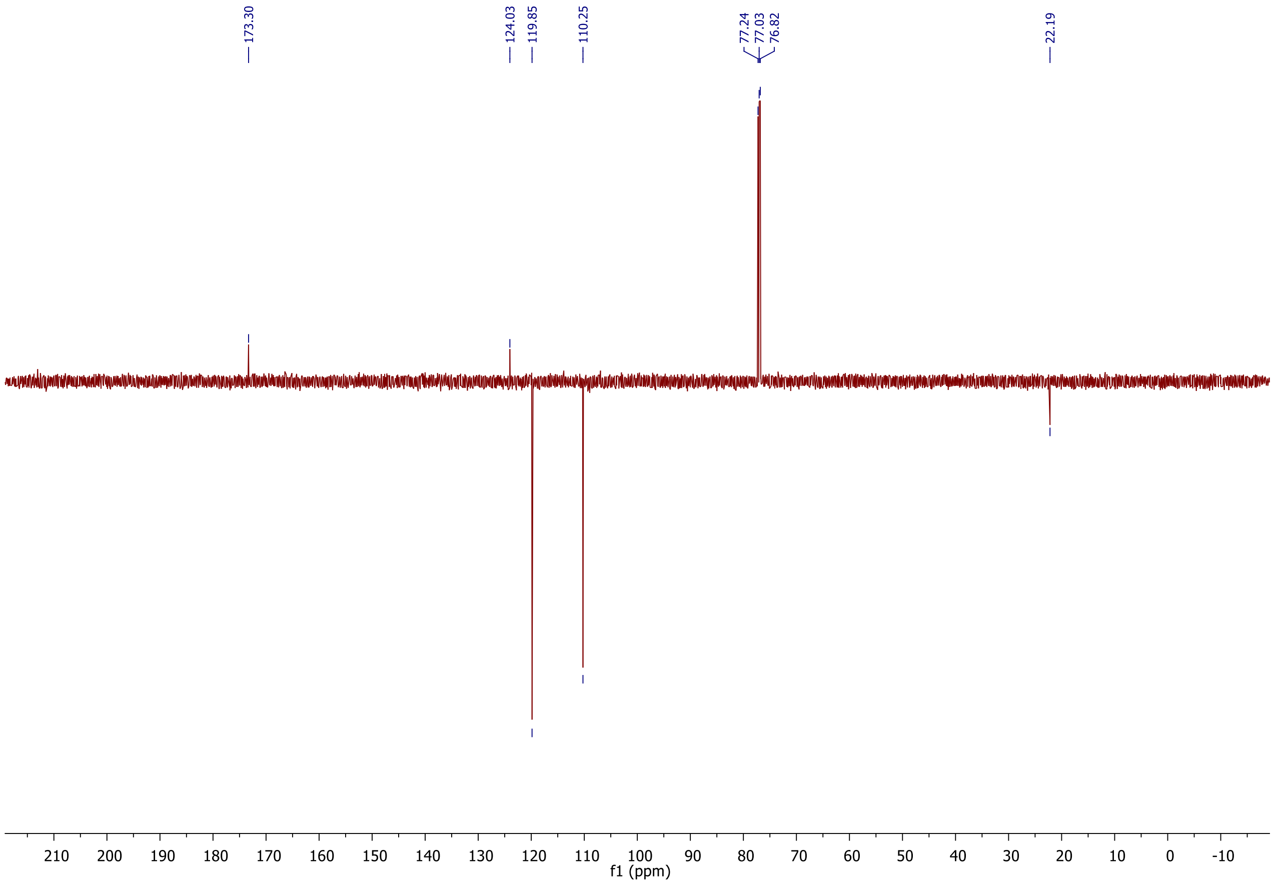

173.30
124.03
119.85
110.25
77.24
77.03
76.82
22.19
210 200 190 180 170 160 150 140 130 120 110 100 90 80 70 60 50 40 30 20 10 0 -10
f1 (ppm)

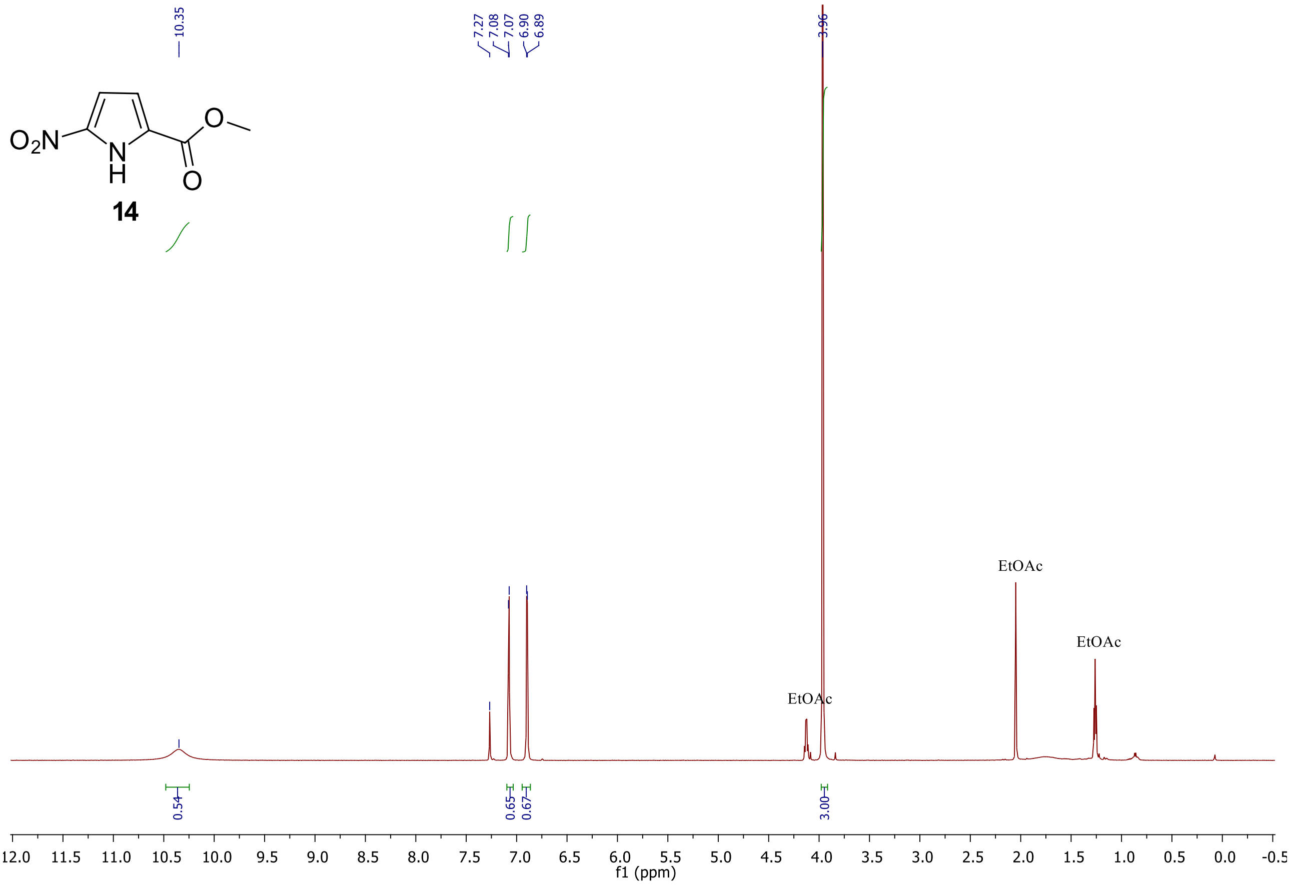
10.35
7.27
7.08
7.07
6.90
6.89
3.96
O_2N
N
H
O
O
14
EtOAc
EtOAc
EtOAc
0.54
0.65
0.67
3.00
12.0 11.5 11.0 10.5 10.0 9.5 9.0 8.5 8.0 7.5 7.0 6.5 6.0 5.5 5.0 4.5 4.0 3.5 3.0 2.5 2.0 1.5 1.0 0.5 0.0 -0.5
f1 (ppm)

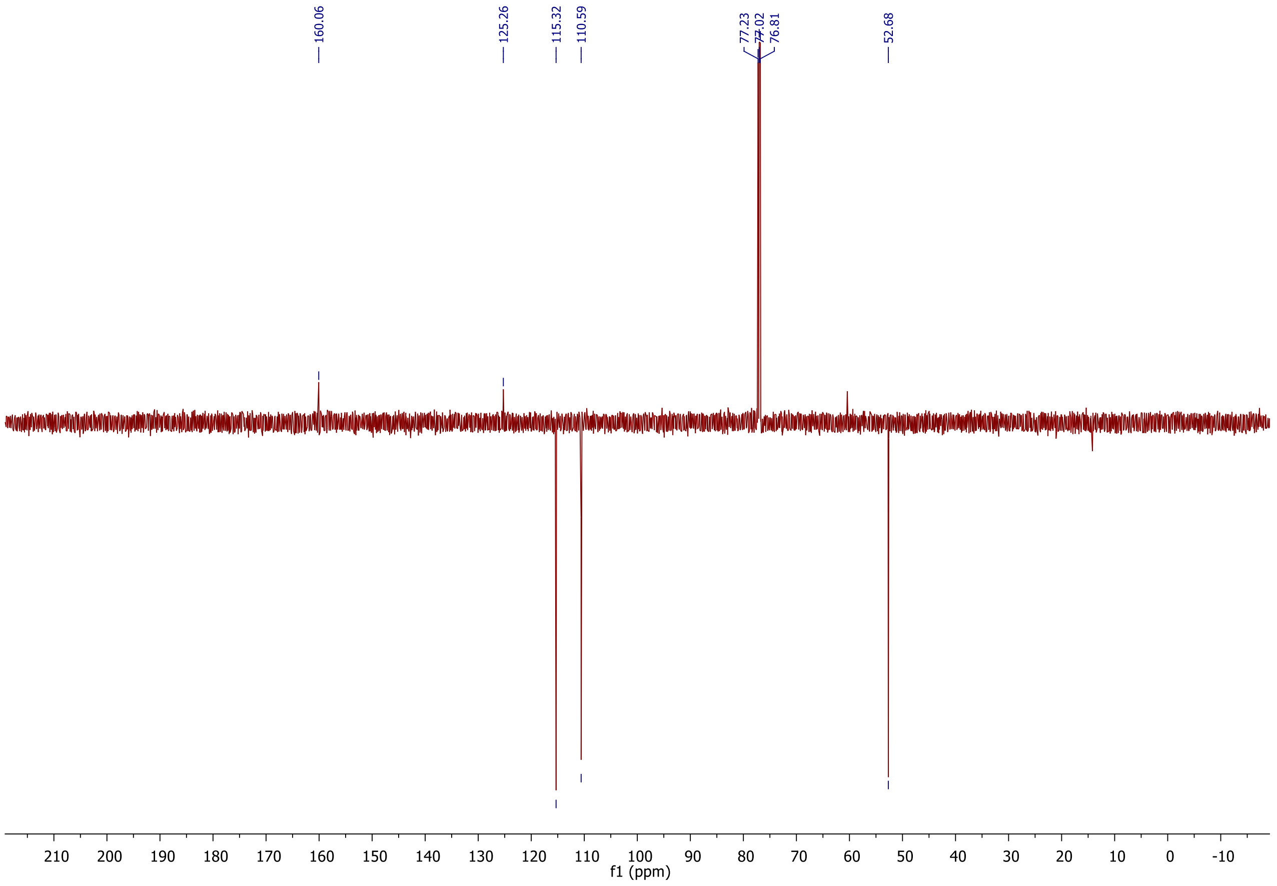
160.06
125.26
115.32
110.59
77.23
77.02
76.81
52.68
210 200 190 180 170 160 150 140 130 120 110 100 90 80 70 60 50 40 30 20 10 0 -10
f1 (ppm)

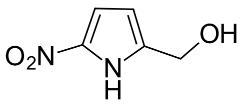
O2N
OH
N
H

15

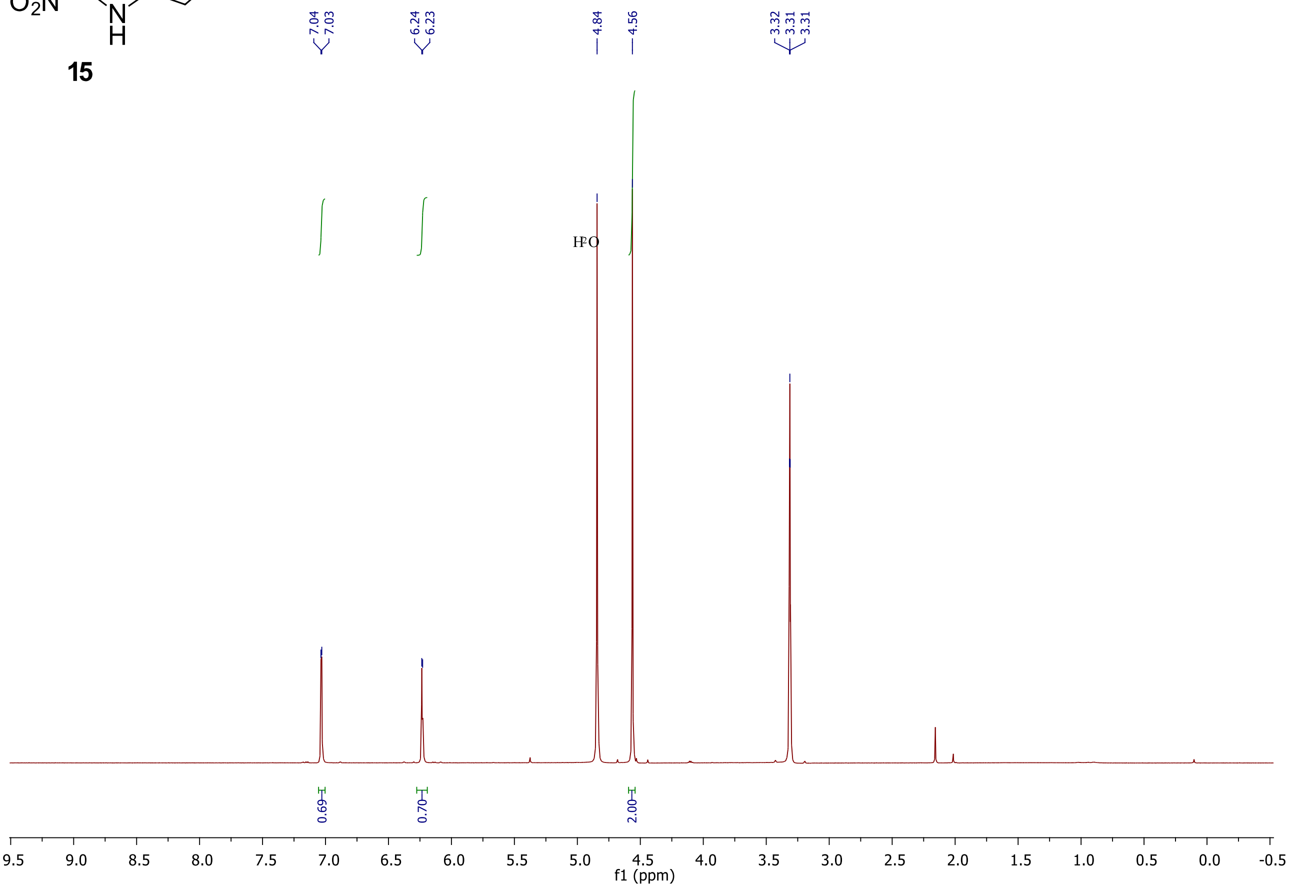
7.04
7.03
6.24
6.23
4.84
4.56
3.32
3.31
3.31
H2O
0.69
0.70
2.00
9.5 9.0 8.5 8.0 7.5 7.0 6.5 6.0 5.5 5.0 4.5 4.0 3.5 3.0 2.5 2.0 1.5 1.0 0.5 0.0 -0.5
f1 (ppm)

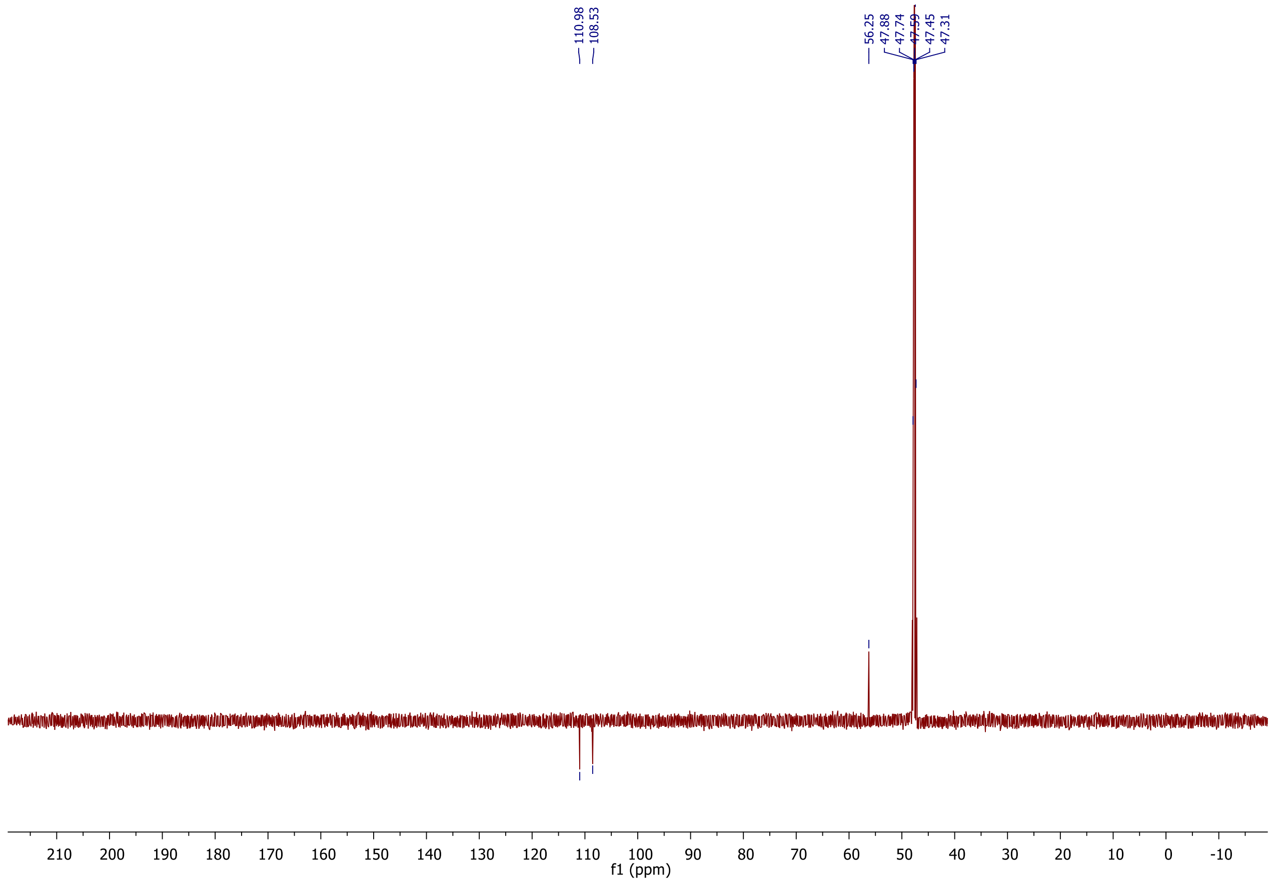
110.98
108.53
56.25
47.88
47.74
47.59
47.45
47.31
210 200 190 180 170 160 150 140 130 120 110 100 90 80 70 60 50 40 30 20 10 0 -10
f1 (ppm)

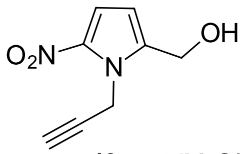
O2N
N
OH

16 NPYM-OH

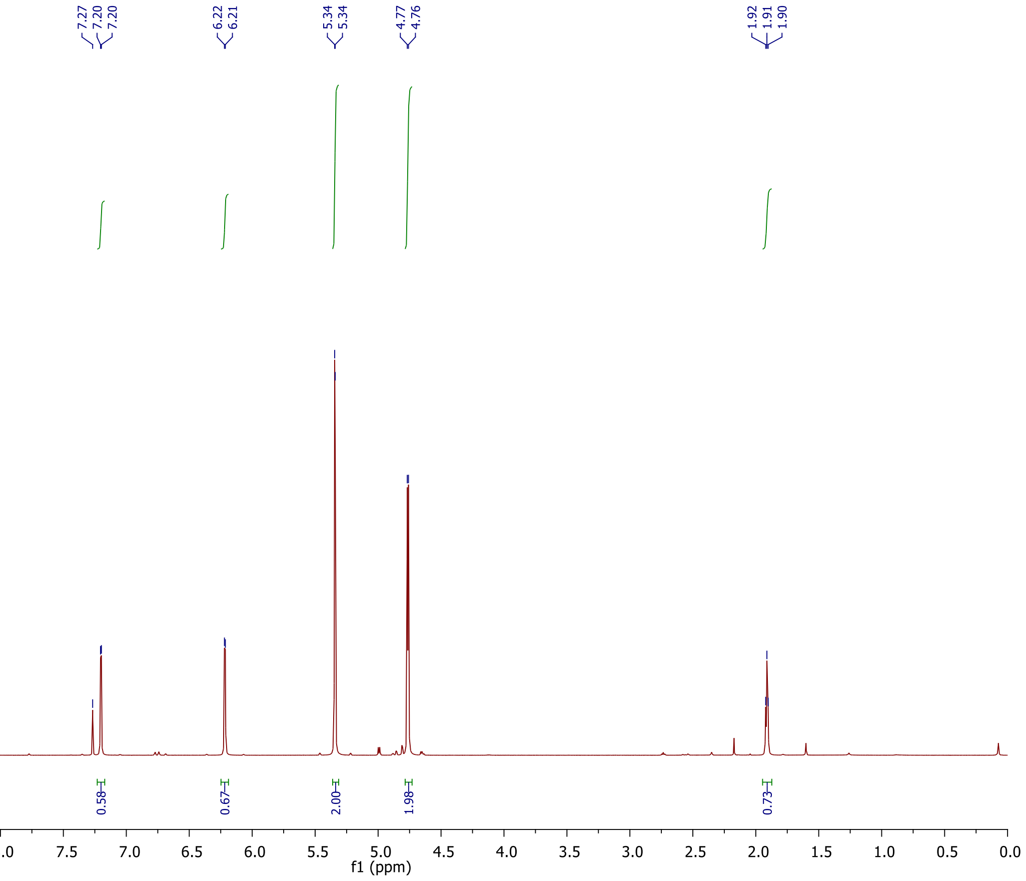
7.27
7.20
7.20
6.22
6.21
5.34
5.34
4.77
4.76
1.92
1.91
1.90
0.58
0.67
2.00
1.98
0.73
10.0 9.5 9.0 8.5 8.0 7.5 7.0 6.5 6.0 5.5 5.0 4.5 4.0 3.5 3.0 2.5 2.0 1.5 1.0 0.5 0.0
f1 (ppm)

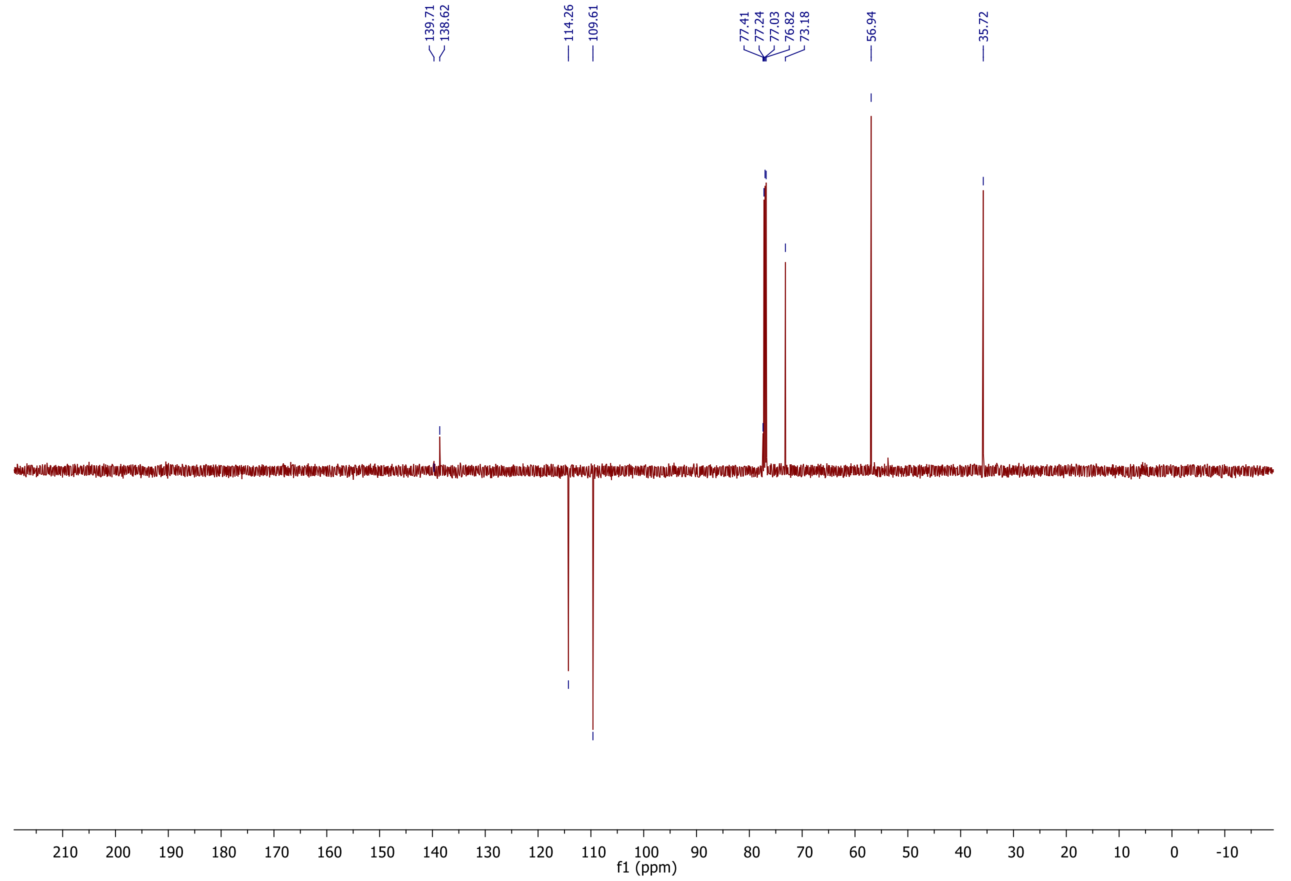
139.71
138.62
114.26
109.61
77.41
77.24
77.03
76.82
73.18
56.94
35.72
210 200 190 180 170 160 150 140 130 120 110 100 90 80 70 60 50 40 30 20 10 0 -10
f1 (ppm)

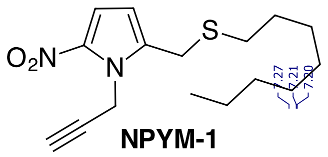

NPYM-1

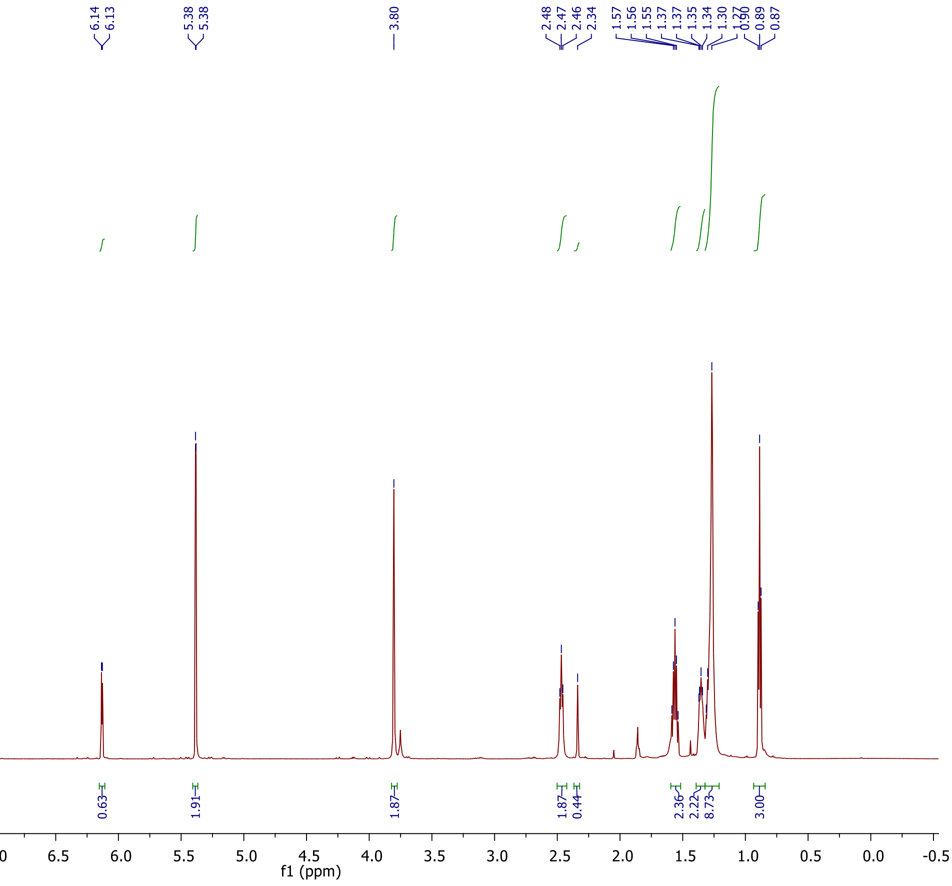

7.27
7.21
7.20
6.14
6.13
5.38
5.38
3.80
2.48
2.47
2.46
2.34
1.57
1.56
1.55
1.37
1.37
1.35
1.34
1.30
1.27
0.90
0.89
0.87
0.49
0.63
1.91
1.87
1.87
0.44
2.36
2.22
8.73
3.00
f1 (ppm)

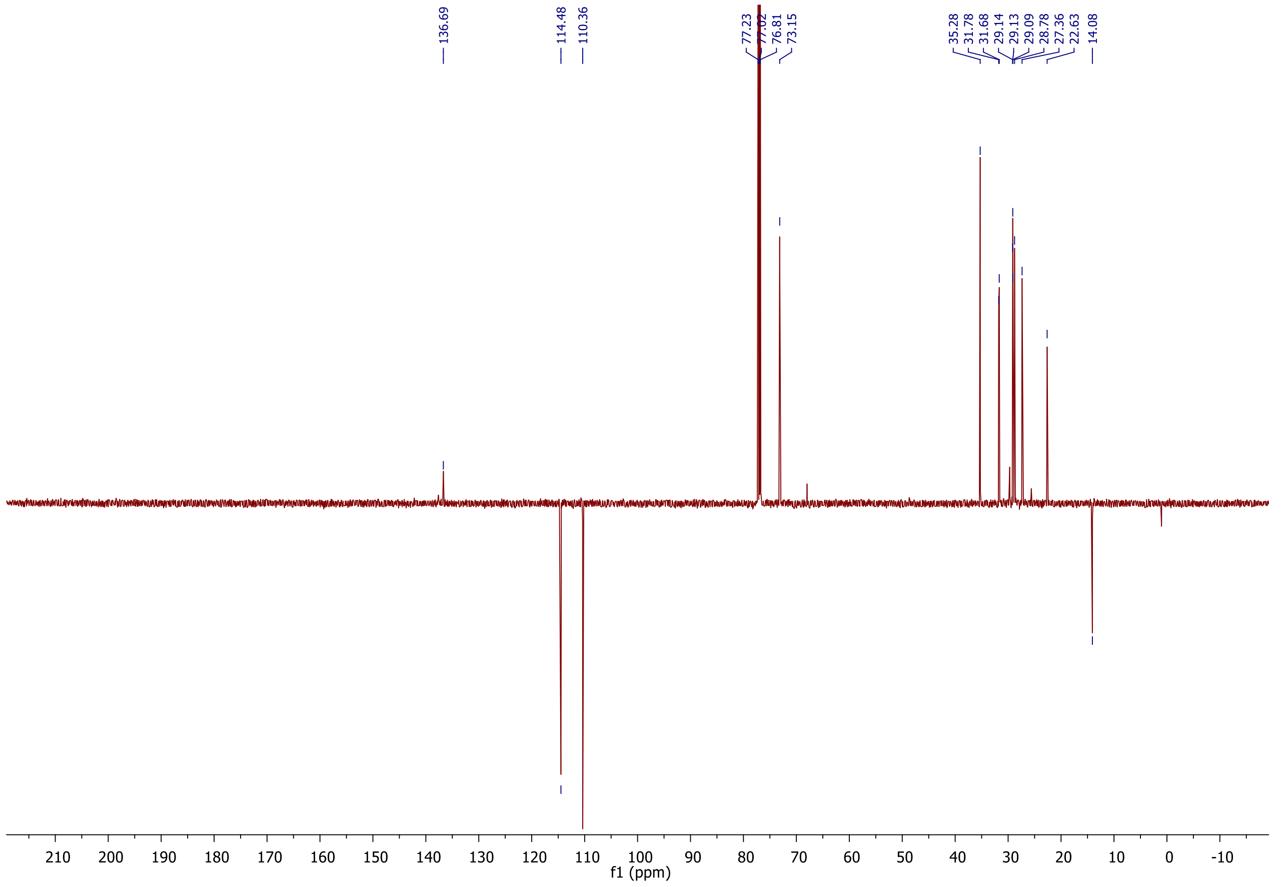

136.69
114.48
110.36
77.23
77.02
76.81
73.15
35.28
31.78
31.68
29.14
29.13
29.09
28.78
27.36
22.63
14.08
f1 (ppm)

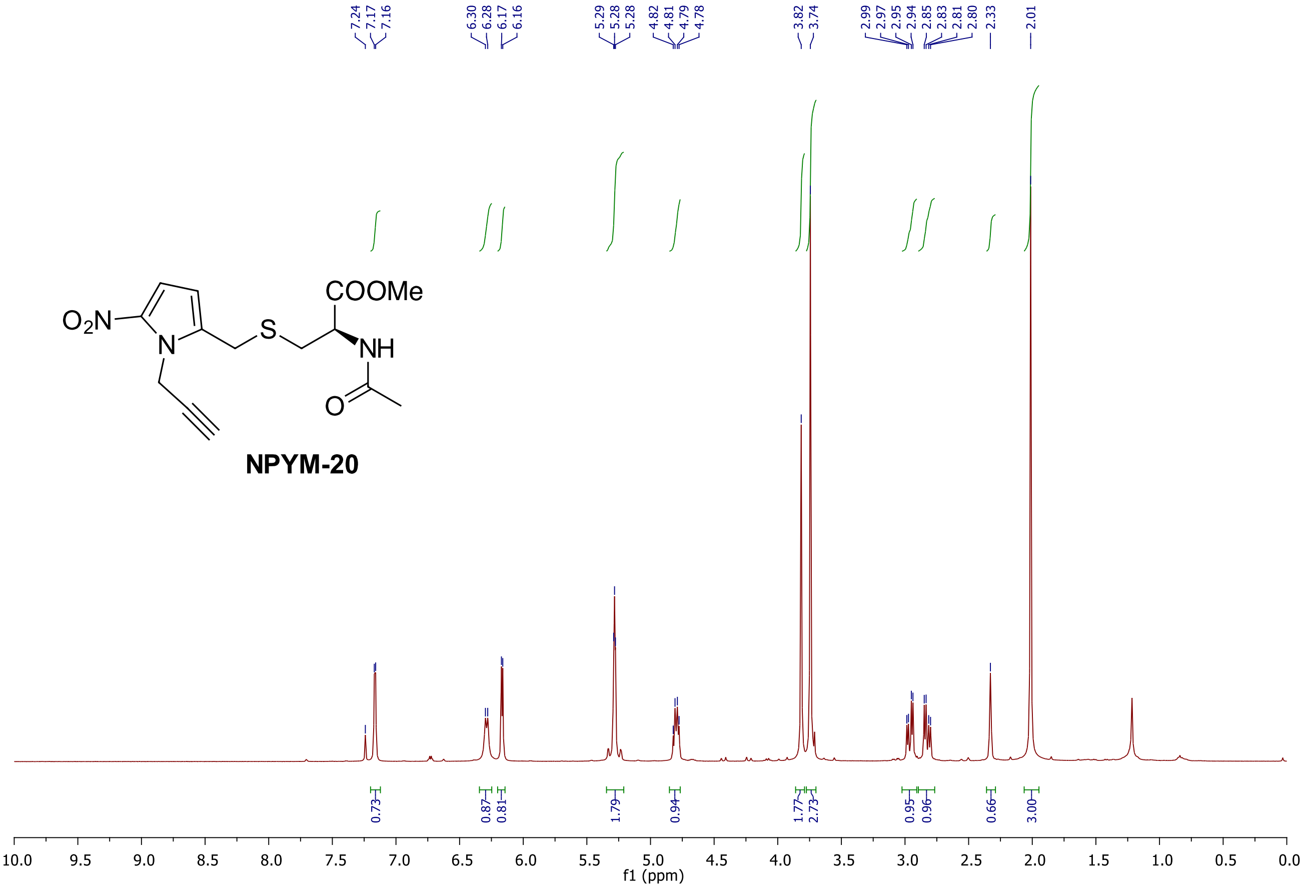
7.24
7.17
7.16
6.30
6.28
6.17
6.16
5.29
5.28
5.28
4.82
4.81
4.79
4.78
3.82
3.74
2.99
2.97
2.95
2.94
2.85
2.83
2.81
2.80
2.33
2.01
0.73
0.87
0.81
1.79
0.94
1.77
2.73
0.95
0.96
0.66
3.00
10.0 9.5 9.0 8.5 8.0 7.5 7.0 6.5 6.0 5.5 5.0 4.5 4.0 3.5 3.0 2.5 2.0 1.5 1.0 0.5 0.0
f1 (ppm)
COOMe
O_2N
N
S
NH
O

NPYM-20

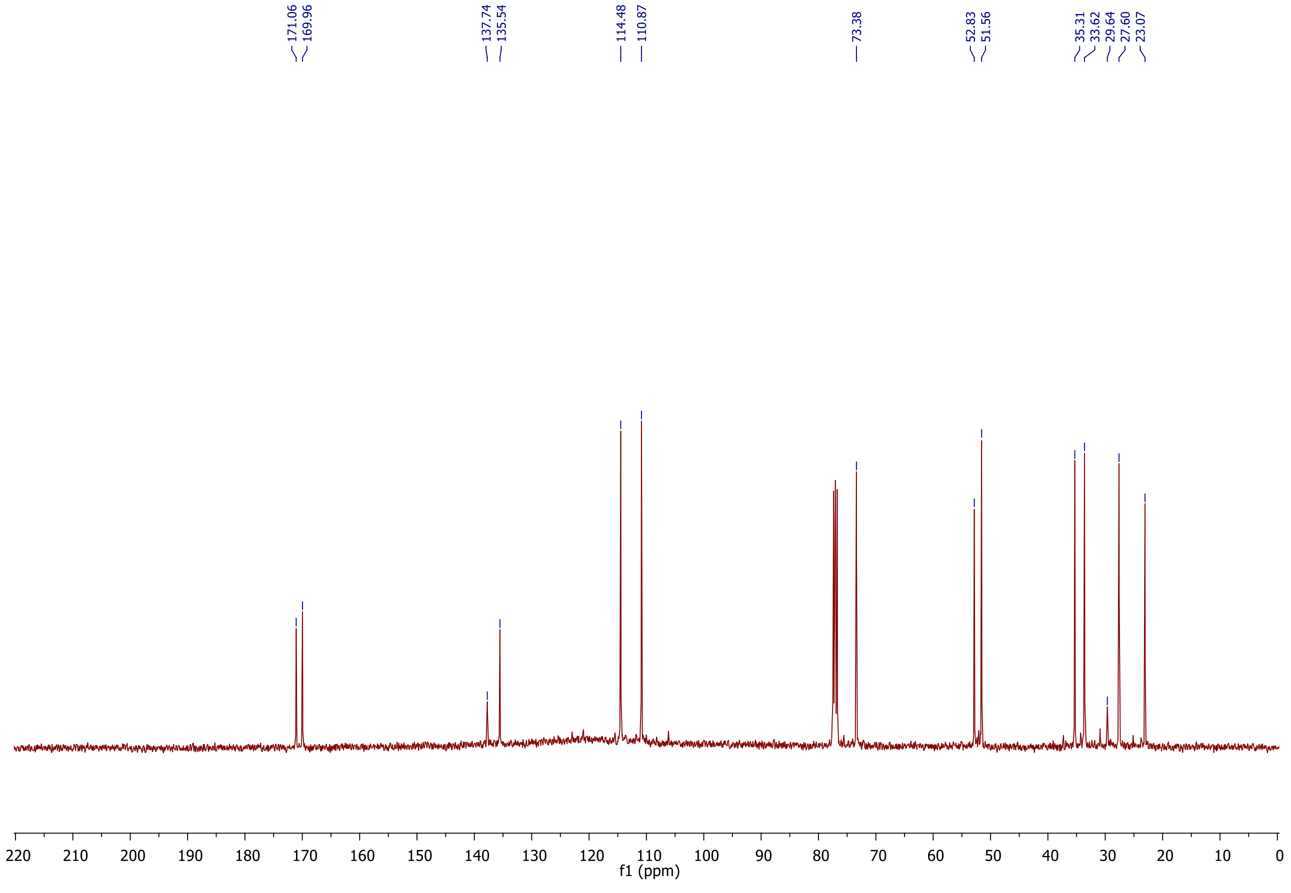
171.06
169.96
137.74
135.54
114.48
110.87
73.38
52.83
51.56
35.31
33.62
29.64
27.60
23.07
220 210 200 190 180 170 160 150 140 130 120 110 100 90 80 70 60 50 40 30 20 10 0
f1 (ppm)

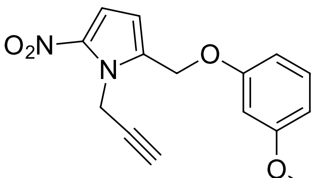
O2N
N
O
O

NPYM-21

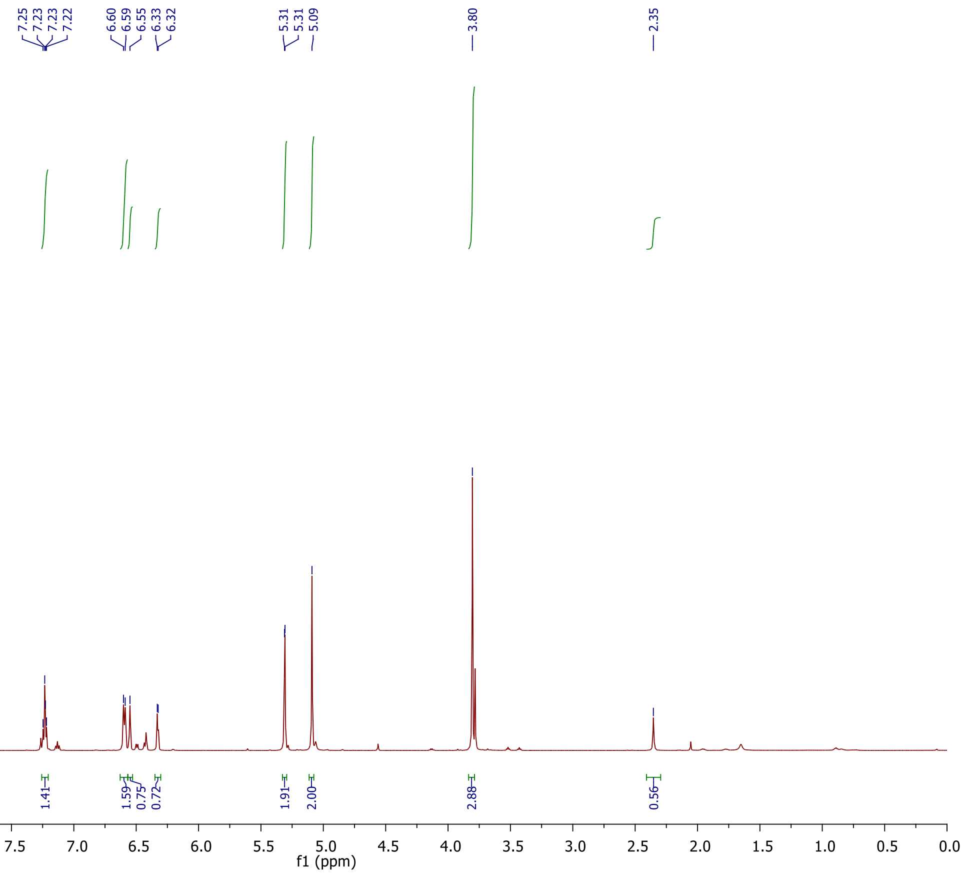
7.25
7.23
7.23
7.22
6.60
6.59
6.55
6.33
6.32
5.31
5.31
5.09
3.80
2.35
1.41
1.59
0.75
0.72
1.91
2.00
2.88
0.56
10.0 9.5 9.0 8.5 8.0 7.5 7.0 6.5 6.0 5.5 5.0 4.5 4.0 3.5 3.0 2.5 2.0 1.5 1.0 0.5 0.0
f1 (ppm)

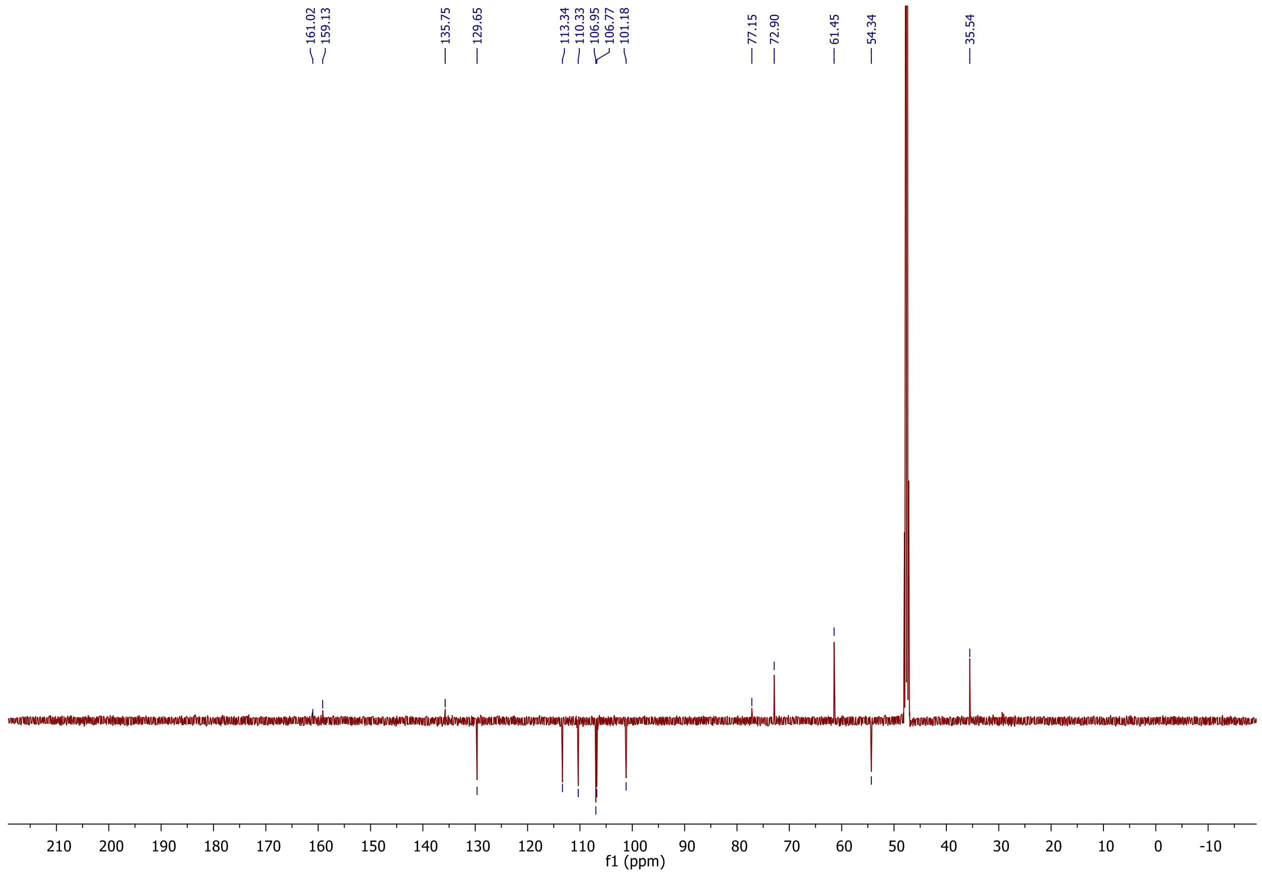
161.02
159.13
135.75
129.65
113.34
110.33
106.95
106.77
101.18
77.15
72.90
61.45
54.34
35.54
210 200 190 180 170 160 150 140 130 120 110 100 90 80 70 60 50 40 30 20 10 0 -10
f1 (ppm)

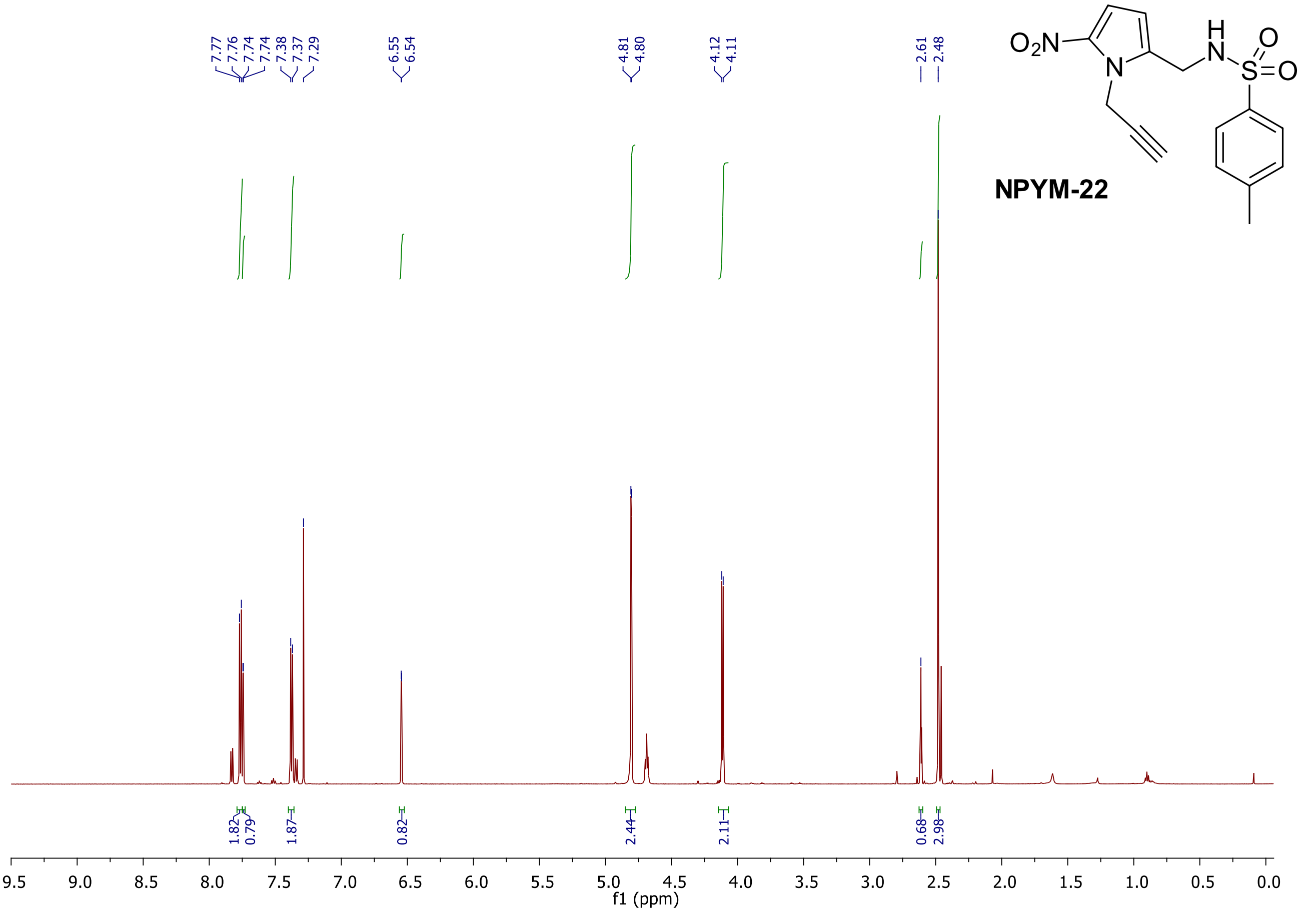
7.77
7.76
7.74
7.74
7.38
7.37
7.29
6.55
6.54
4.81
4.80
4.12
4.11
2.61
2.48
O_2N
N
H
N
S
O
O
1.82
0.79
1.87
0.82
2.44
2.11
0.68
2.98
9.5 9.0 8.5 8.0 7.5 7.0 6.5 6.0 5.5 5.0 4.5 4.0 3.5 3.0 2.5 2.0 1.5 1.0 0.5 0.0
f1 (ppm)

NPYM-22

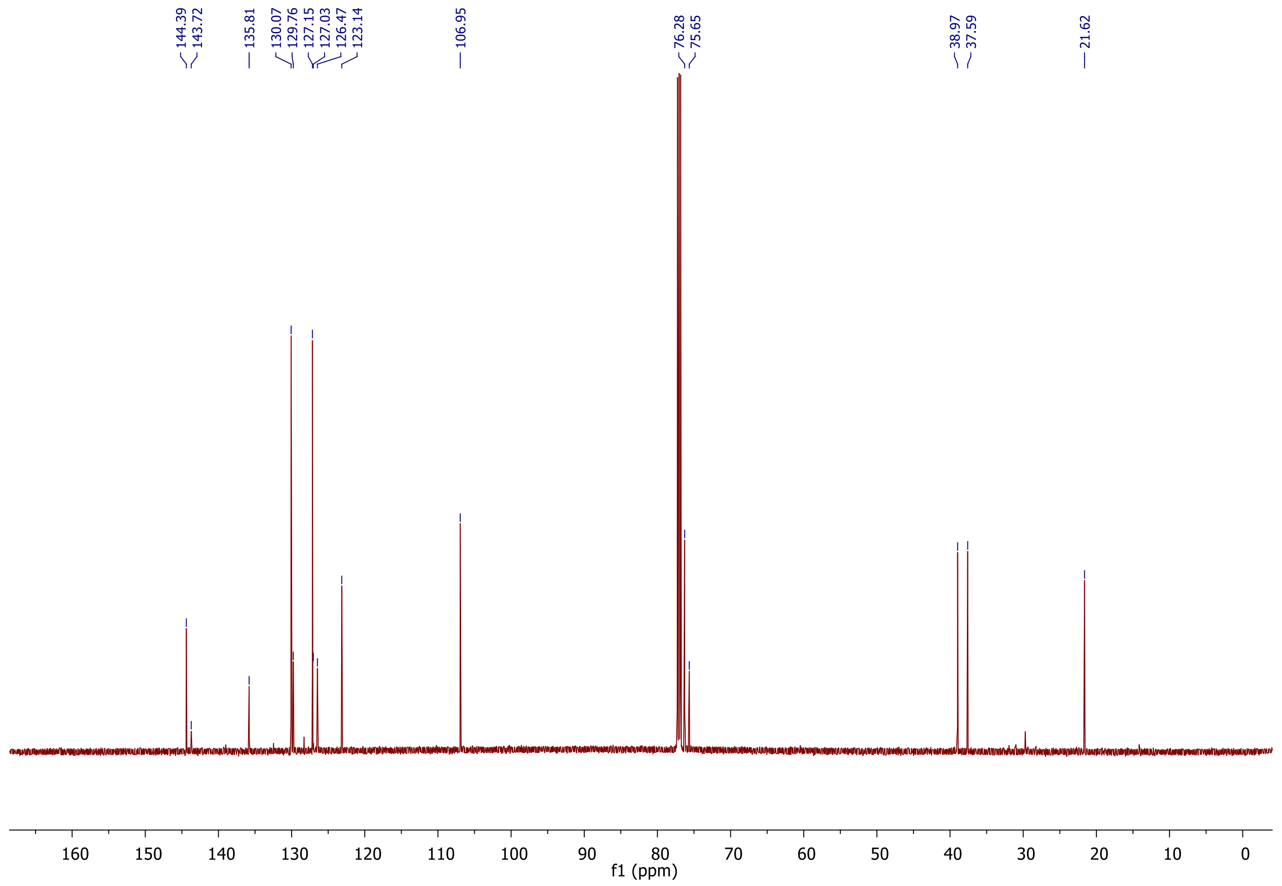
144.39
143.72
135.81
130.07
129.76
127.15
127.03
126.47
123.14
106.95
76.28
75.65
38.97
37.59
21.62
160 150 140 130 120 110 100 90 80 70 60 50 40 30 20 10 0
f1 (ppm)

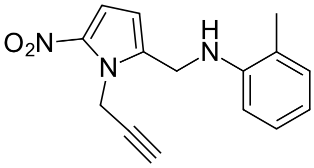

NPYM-23

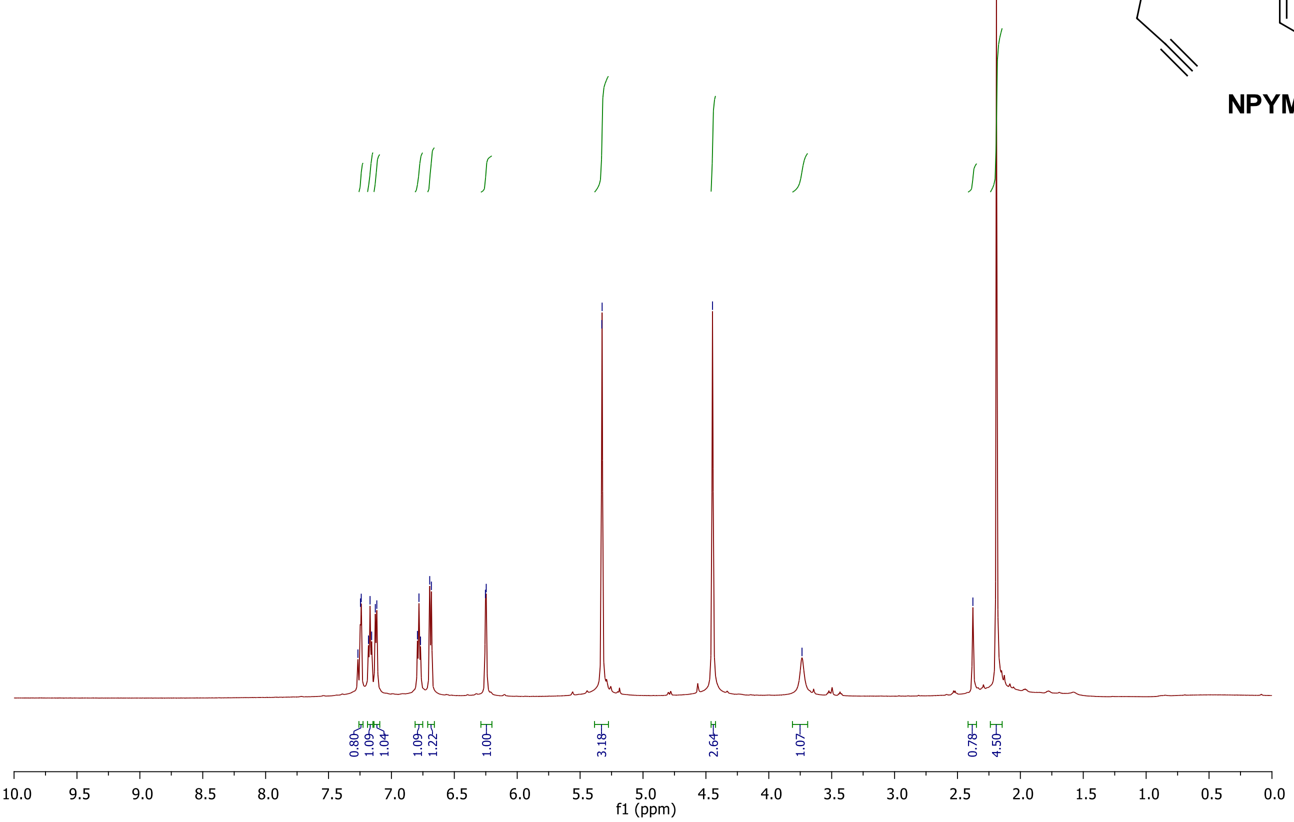

7.27
7.25
7.24
7.18
7.17
7.16
7.13
7.12
6.79
6.78
6.70
6.68
6.25
6.25
5.33
5.32
4.45
3.74
2.38
2.19
0.80
1.09
1.04
1.09
1.22
1.00
3.18
2.64
1.07
0.78
4.50
f1 (ppm)

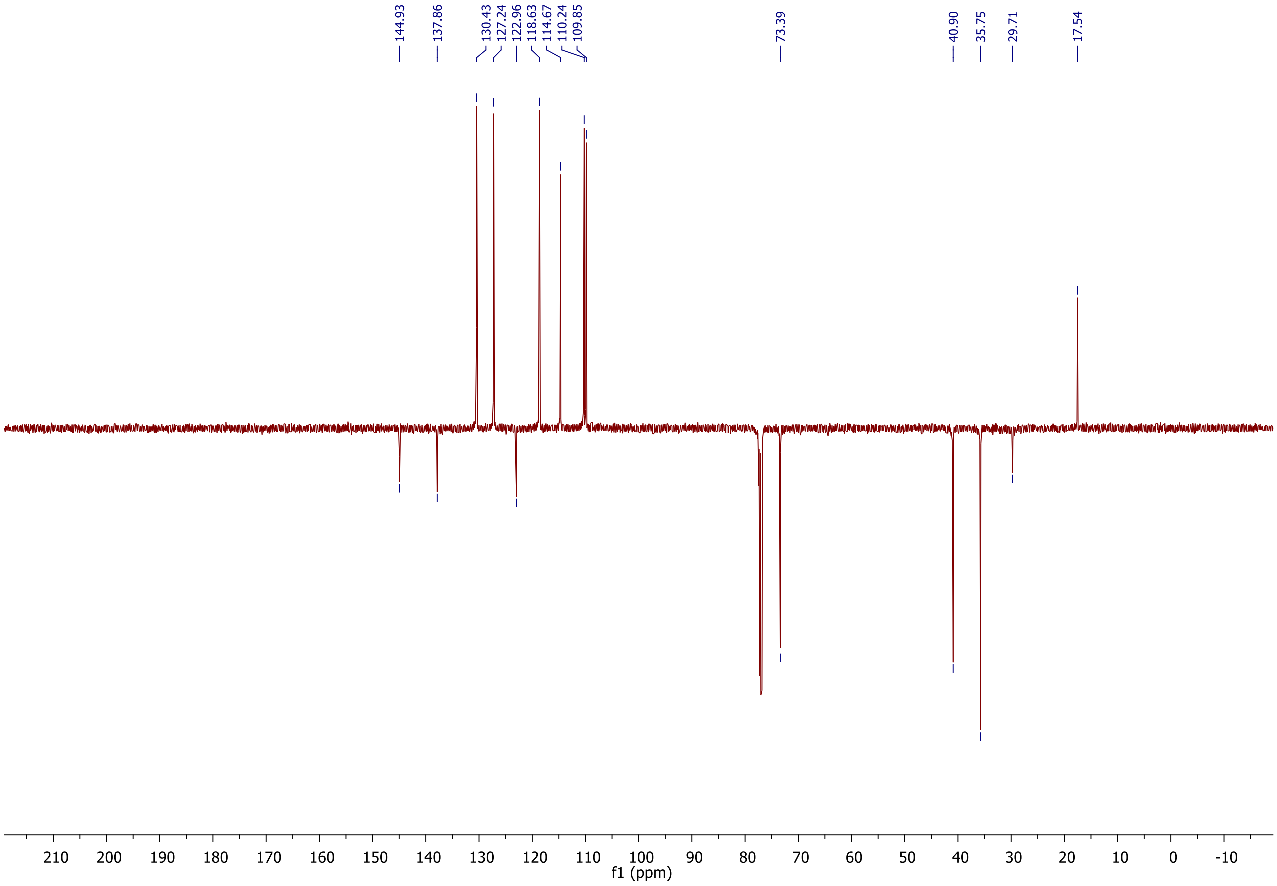

144.93
137.86
130.43
127.24
122.96
118.63
114.67
110.24
109.85
73.39
40.90
35.75
29.71
17.54
f1 (ppm)

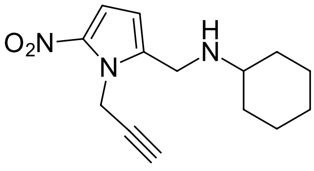
O2N
N
H
N

NPYM-24

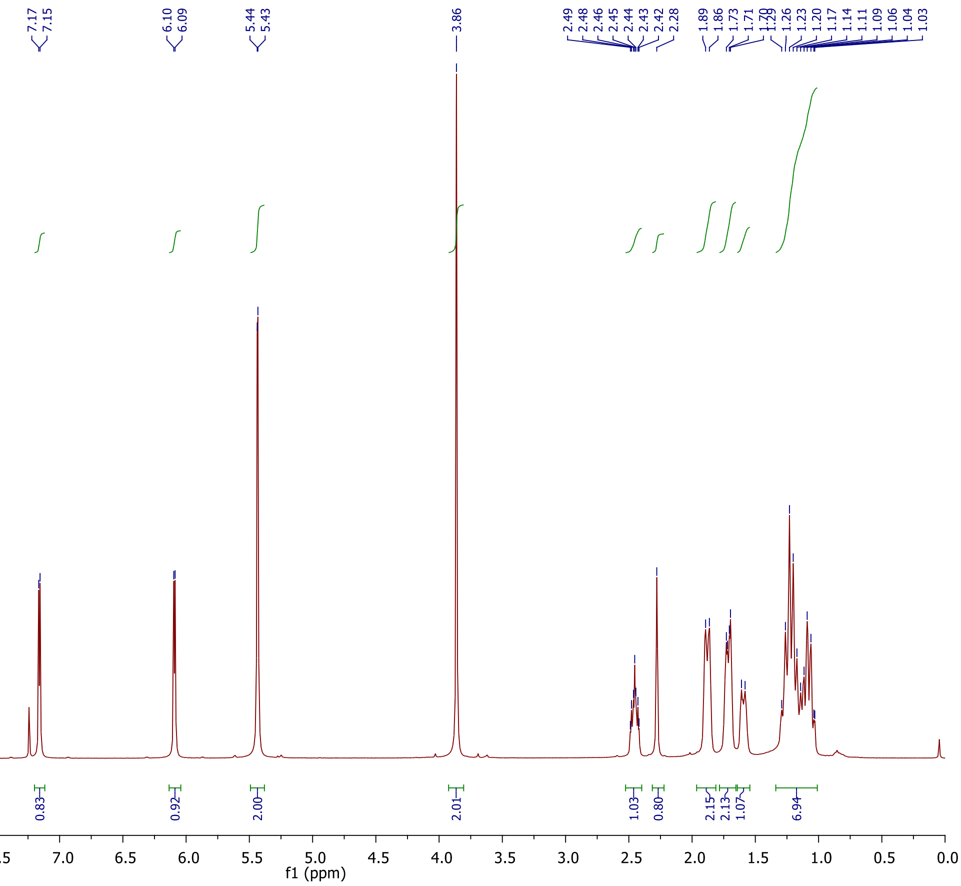
7.17
7.15
6.10
6.09
5.44
5.43
3.86
2.49
2.48
2.46
2.45
2.44
2.43
2.42
2.28
1.89
1.86
1.73
1.71
1.26
1.23
1.20
1.17
1.14
1.11
1.09
1.06
1.04
1.03
0.83
0.92
2.00
2.01
1.03
0.80
2.15
2.13
1.07
6.94
f1 (ppm)

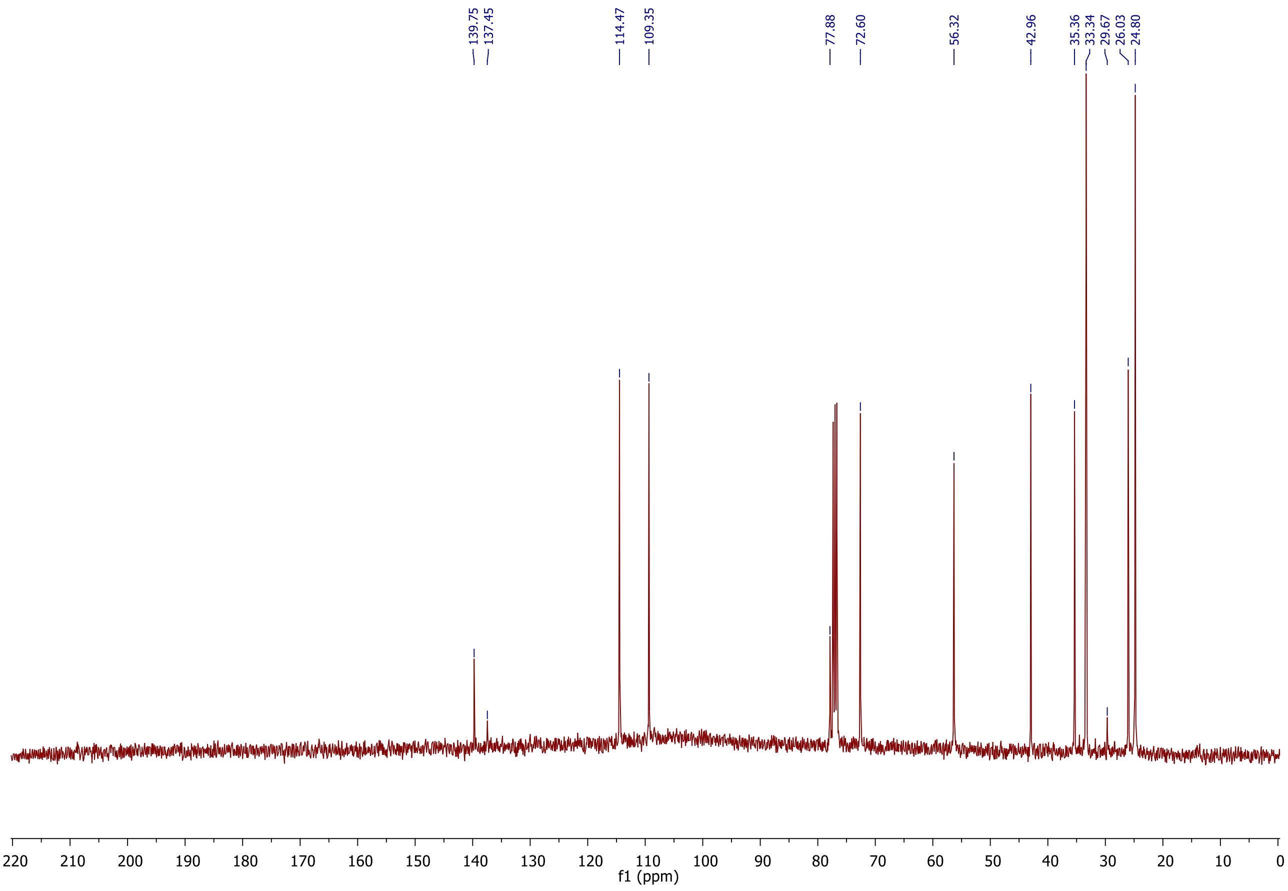
139.75
137.45
114.47
109.35
77.88
72.60
56.32
42.96
35.36
33.34
29.67
26.03
24.80
f1 (ppm)

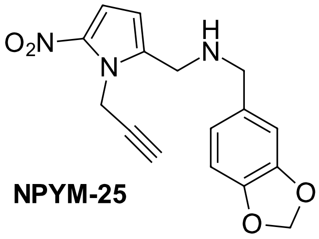
O2N
N
H
N
O
O

NPYM-25

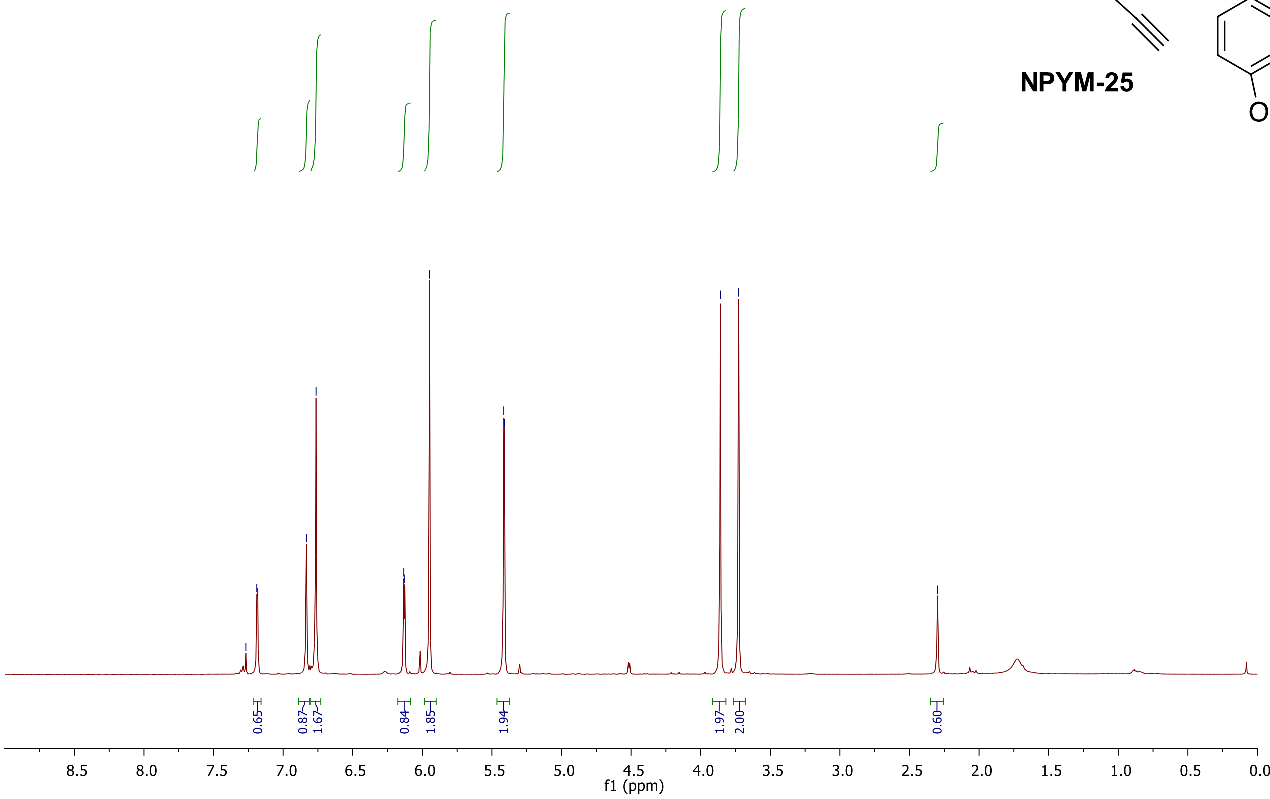
7.27
7.19
7.18
6.83
6.76
6.13
6.13
5.95
5.41
5.41
3.86
3.73
2.30
0.65
0.87
1.67
0.84
1.85
1.94
1.97
2.00
0.60
8.5 8.0 7.5 7.0 6.5 6.0 5.5 5.0 4.5 4.0 3.5 3.0 2.5 2.0 1.5 1.0 0.5 0.0
f1 (ppm)

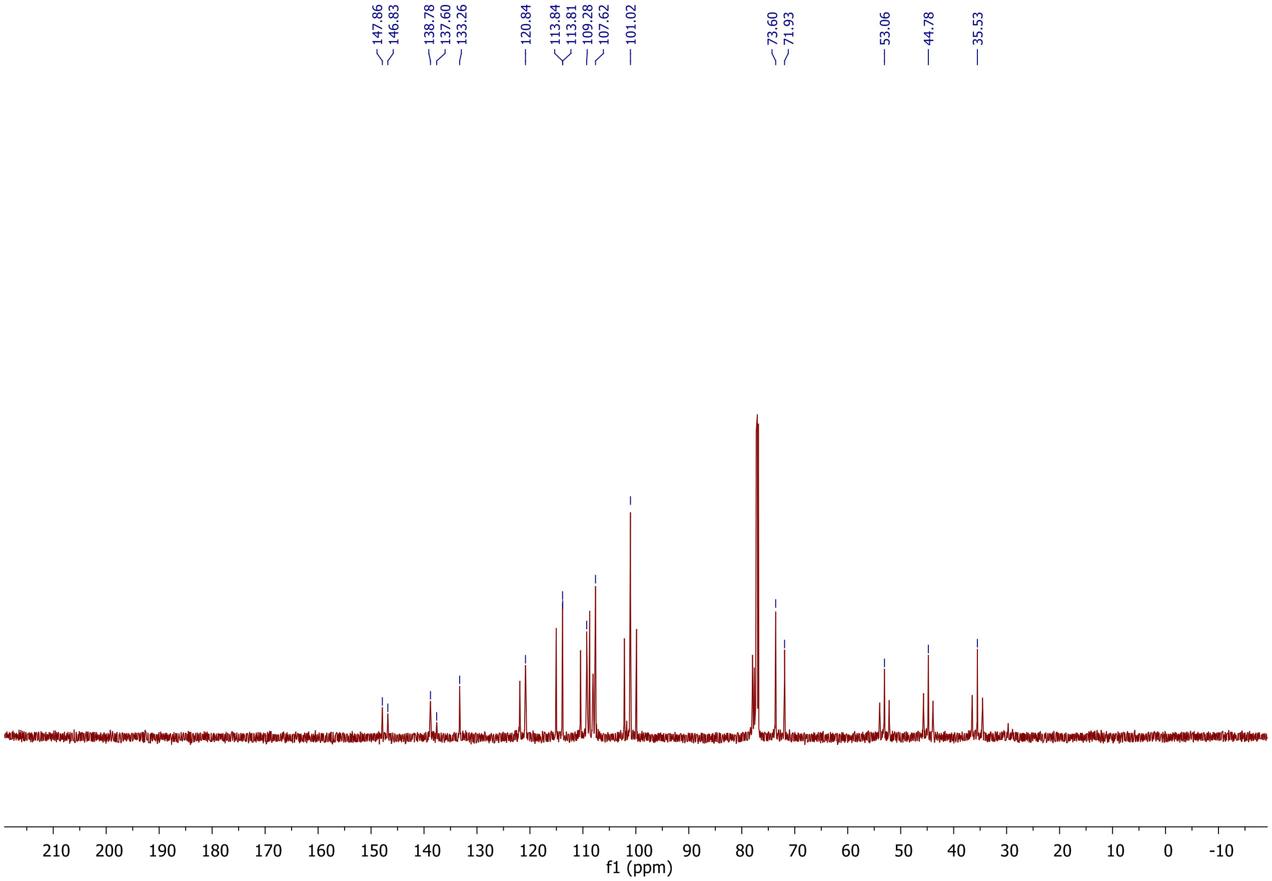
147.86
146.83
138.78
137.60
133.26
120.84
113.84
113.81
109.28
107.62
101.02
73.60
71.93
53.06
44.78
35.53
210 200 190 180 170 160 150 140 130 120 110 100 90 80 70 60 50 40 30 20 10 0 -10
f1 (ppm)

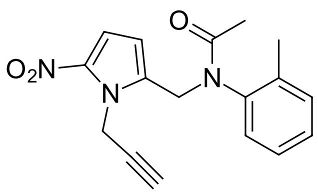

NPYM-26

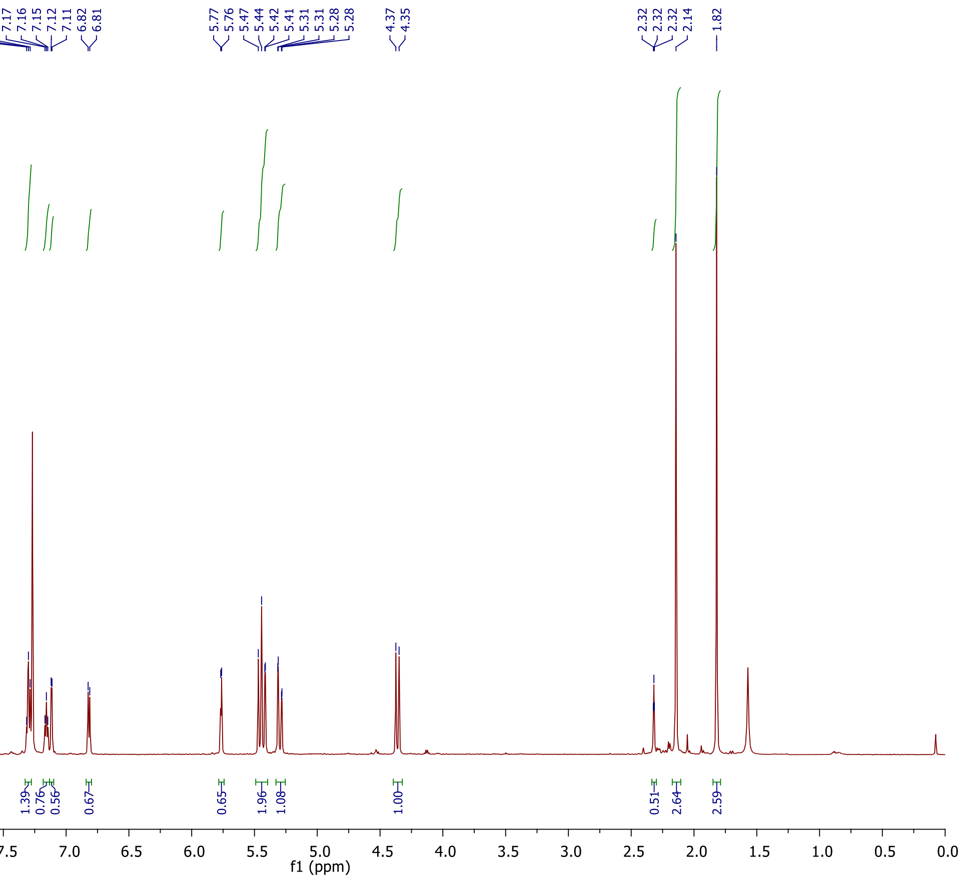

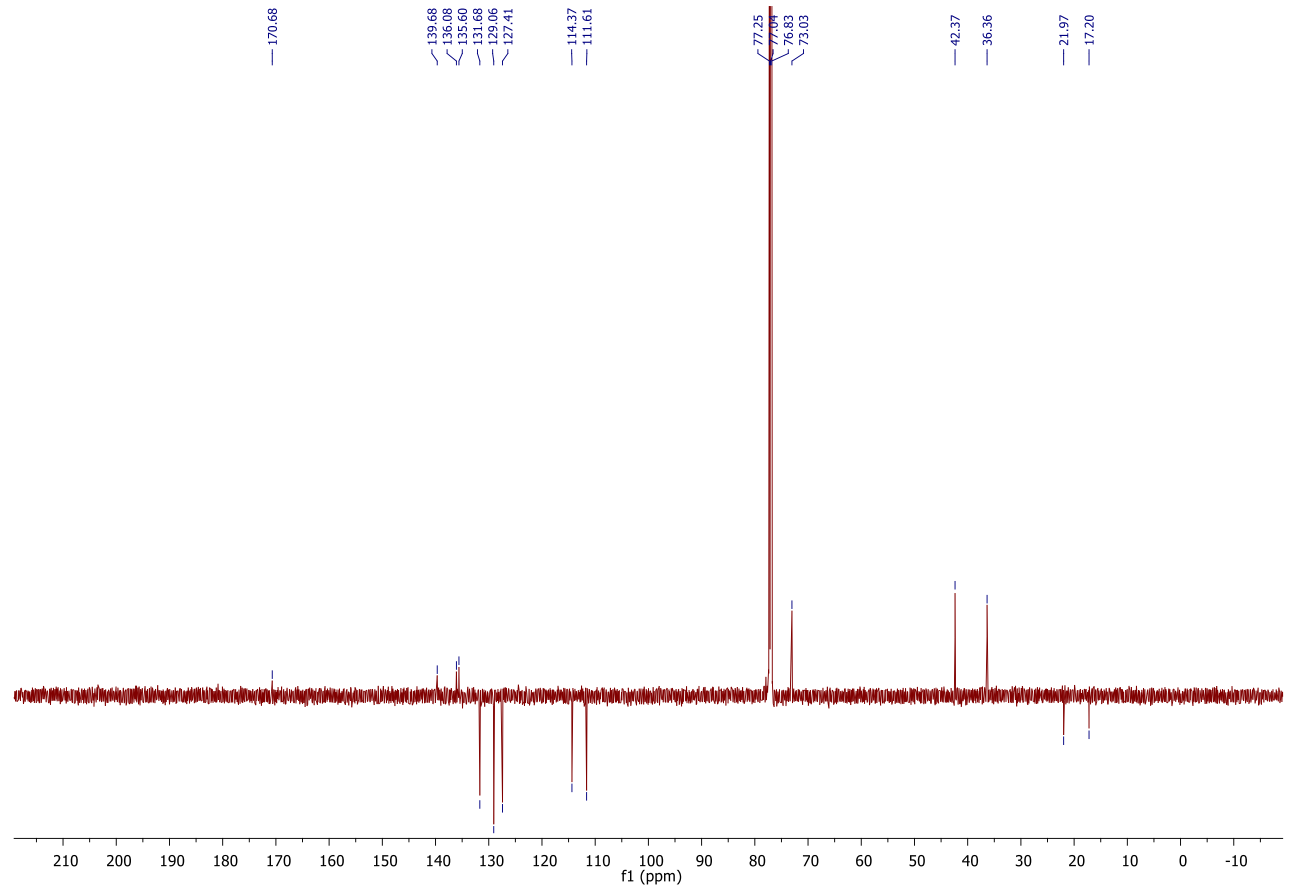

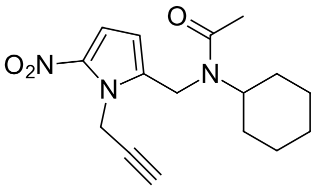
O2N
N
O
N

NPYM-27

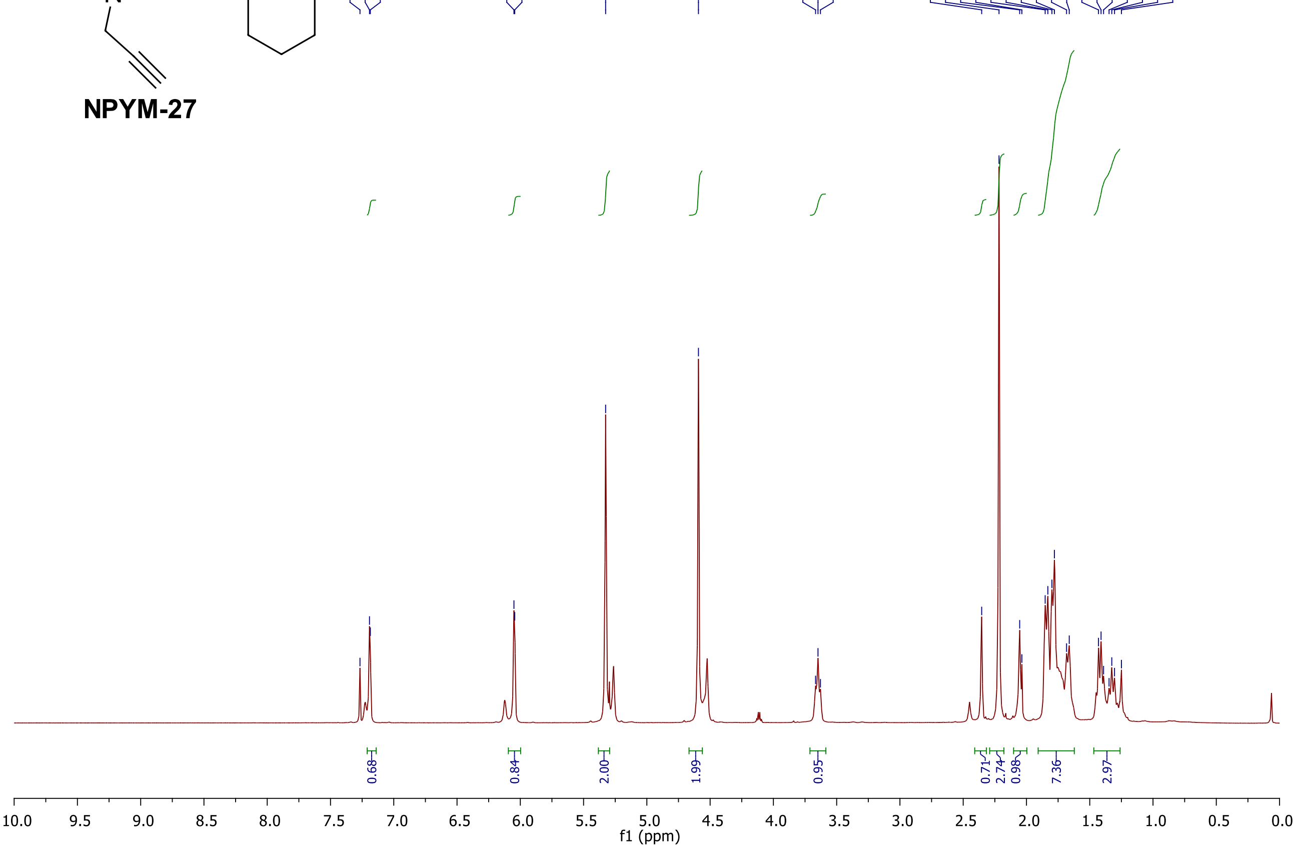
7.27
7.19
7.19
6.05
6.04
5.33
4.59
3.67
3.65
3.63
2.35
2.22
2.05
2.04
1.85
1.83
1.80
1.78
1.68
1.66
1.43
1.41
1.39
1.35
1.33
1.30
1.25
0.68
0.84
2.00
1.99
0.95
0.71
2.74
0.98
7.36
2.97
f1 (ppm)

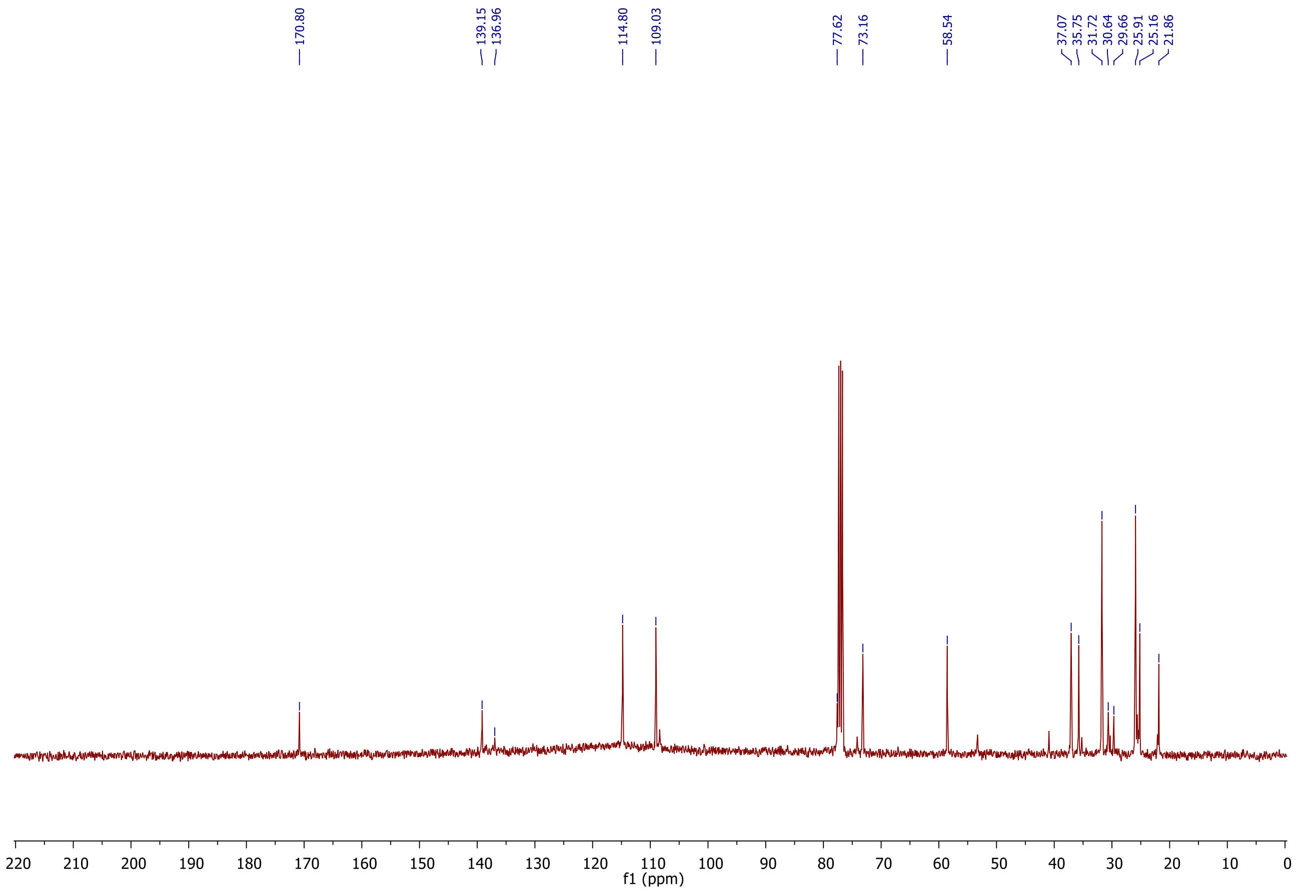
170.80
139.15
136.96
114.80
109.03
77.62
73.16
58.54
37.07
35.75
31.72
30.64
29.66
25.91
25.16
21.86
f1 (ppm)

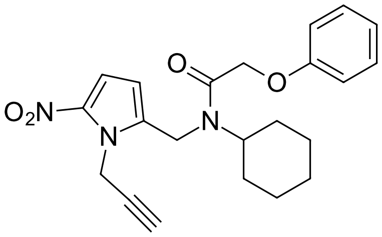

NPYM-28

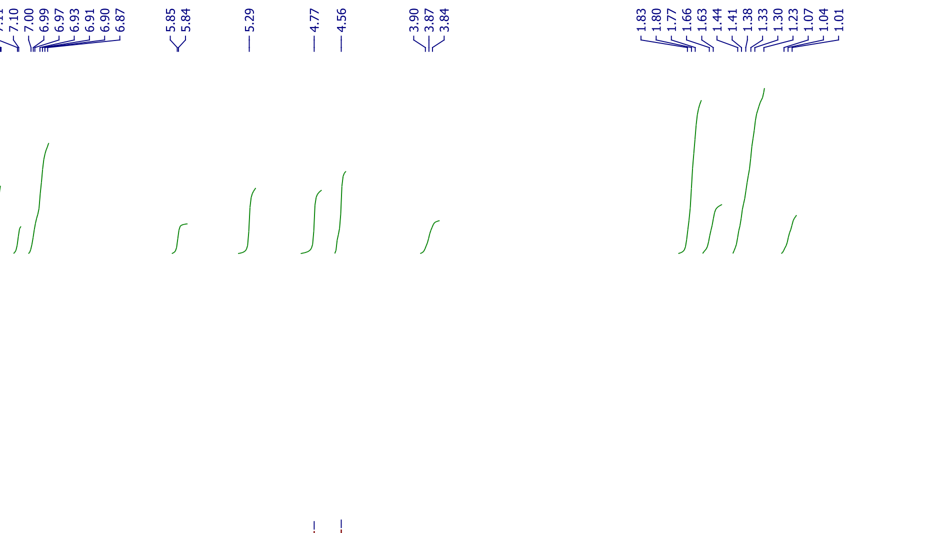
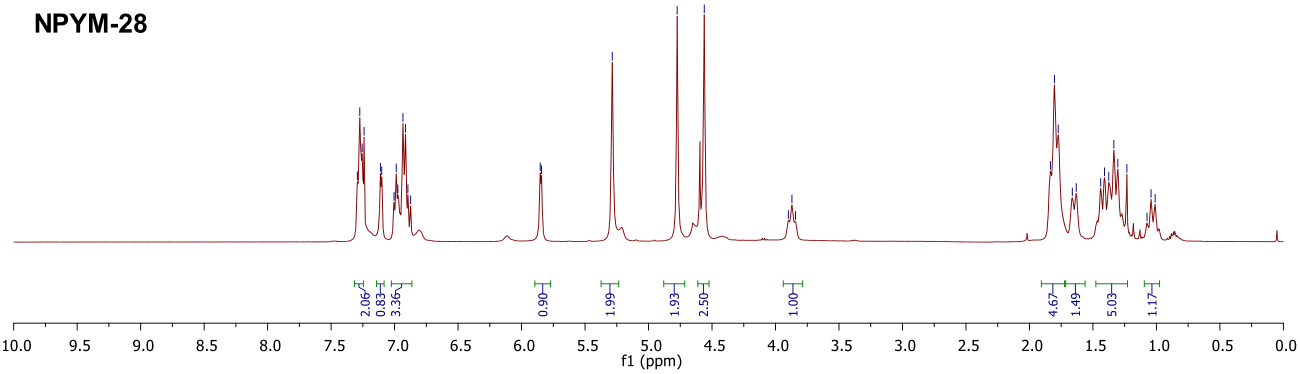
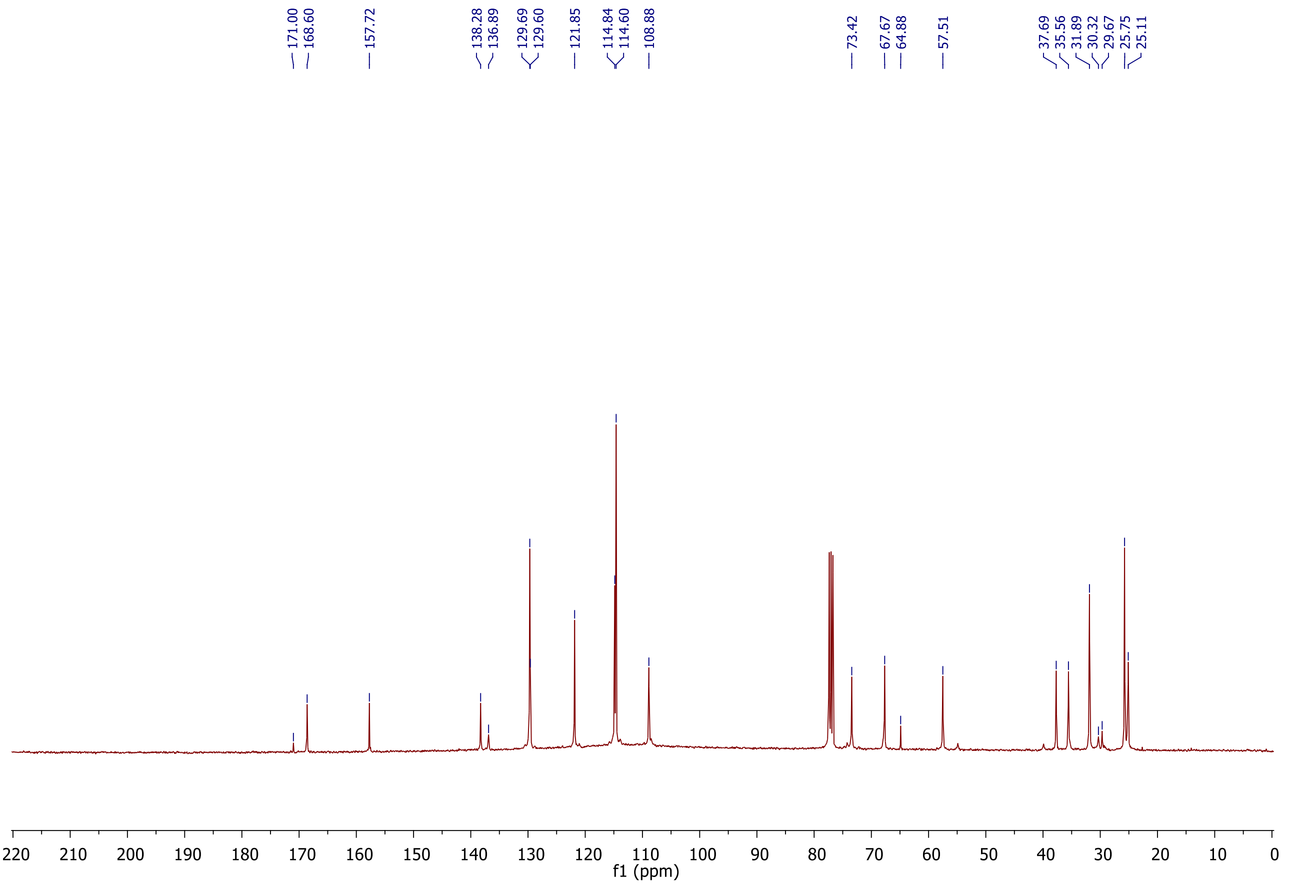
7.29
7.27
7.26
7.25
7.24
7.11
7.10
7.00
6.99
6.97
6.93
6.91
6.90
6.87
5.85
5.84
5.29
4.77
4.56
3.90
3.87
3.84
1.83
1.80
1.77
1.66
1.63
1.44
1.41
1.38
1.33
1.30
1.23
1.07
1.04
1.01
2.06
0.83
3.36
0.90
1.99
1.93
2.50
1.00
4.67
1.49
5.03
1.17
f1 (ppm)
171.00
168.60
157.72
138.28
136.89
129.69
129.60
121.85
114.84
114.60
108.88
73.42
67.67
64.88
57.51
37.69
35.56
31.89
30.32
29.67
25.75
25.11
f1 (ppm)

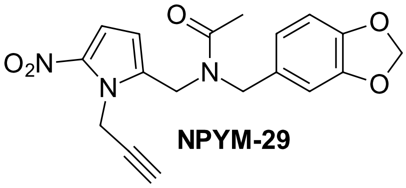

O_2N
O
N
O
O

NPYM-29

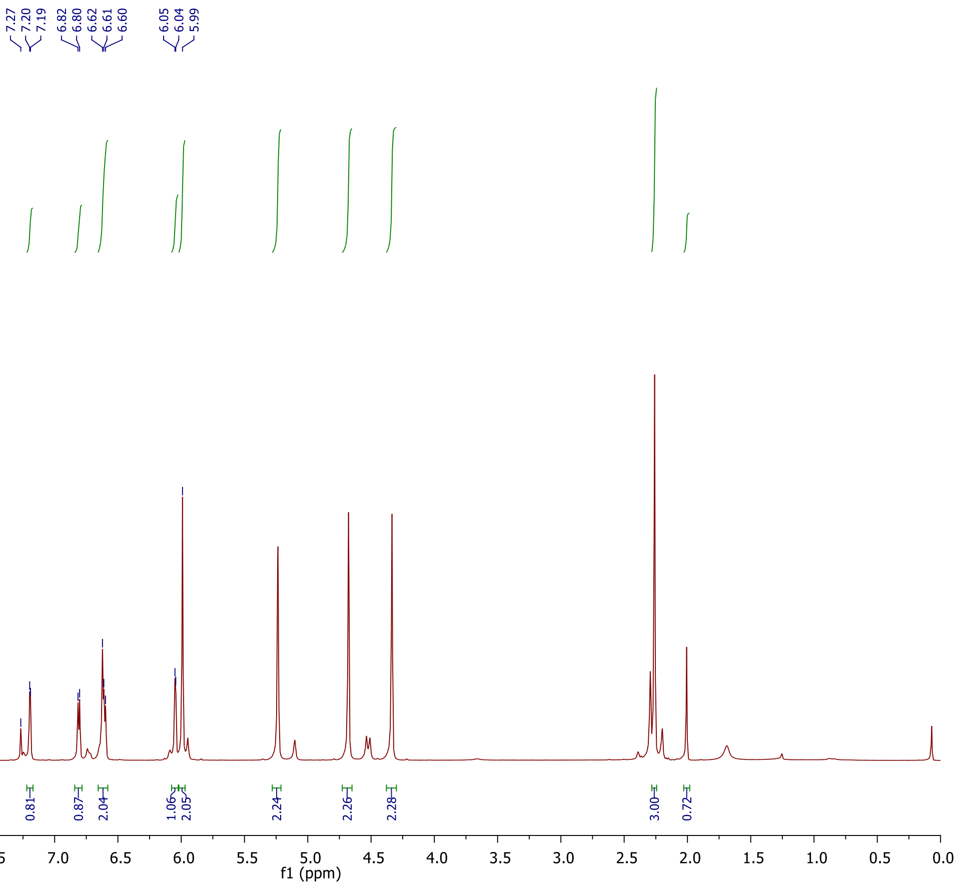

7.27
7.20
7.19
6.82
6.80
6.62
6.61
6.60
6.05
6.04
5.99
0.81
0.87
2.04
1.06
2.05
2.24
2.26
2.28
3.00
0.72
10.0 9.5 9.0 8.5 8.0 7.5 7.0 6.5 6.0 5.5 5.0 4.5 4.0 3.5 3.0 2.5 2.0 1.5 1.0 0.5 0.0
f1 (ppm)

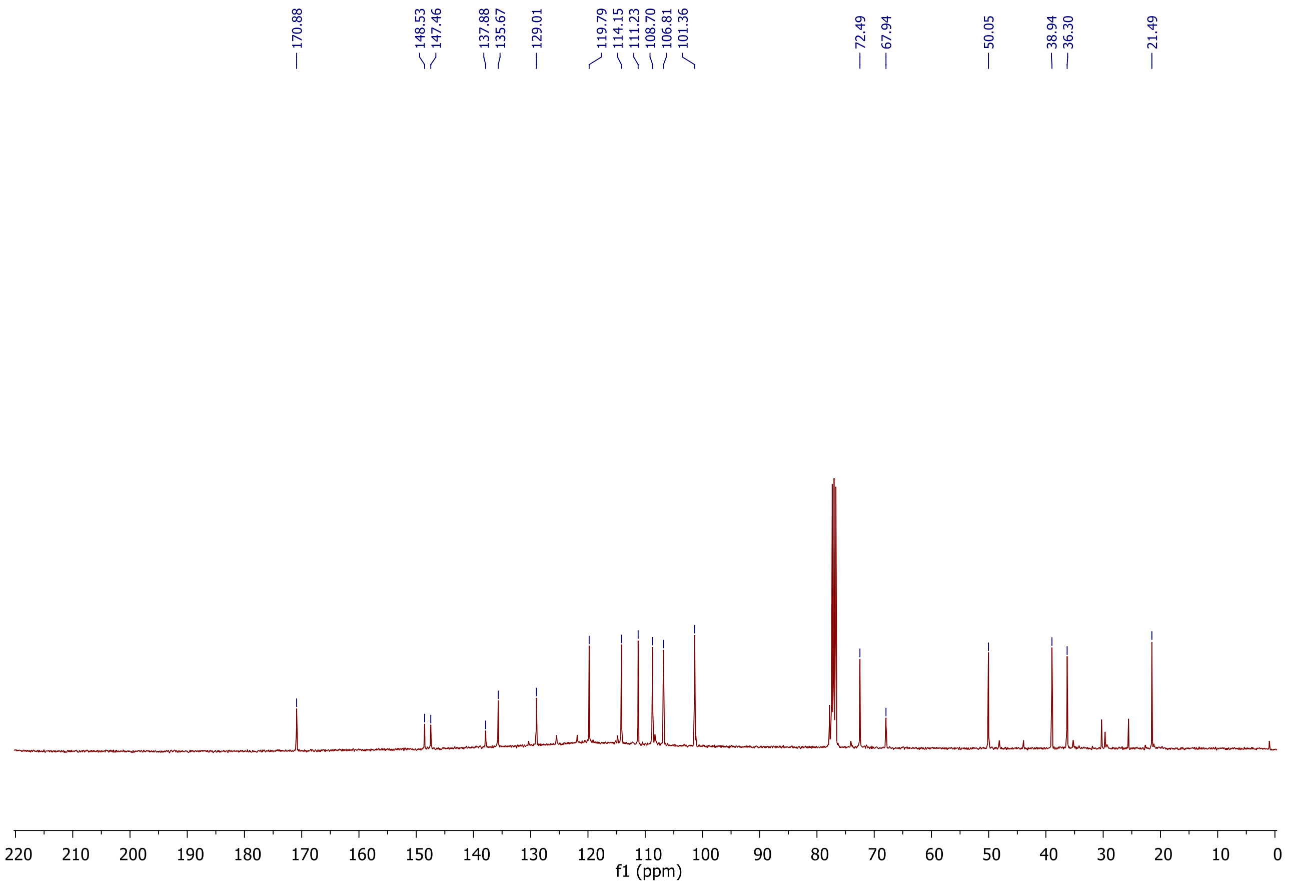

170.88
148.53
147.46
137.88
135.67
129.01
119.79
114.15
111.23
108.70
106.81
101.36
72.49
67.94
50.05
38.94
36.30
21.49
220 210 200 190 180 170 160 150 140 130 120 110 100 90 80 70 60 50 40 30 20 10 0
f1 (ppm)

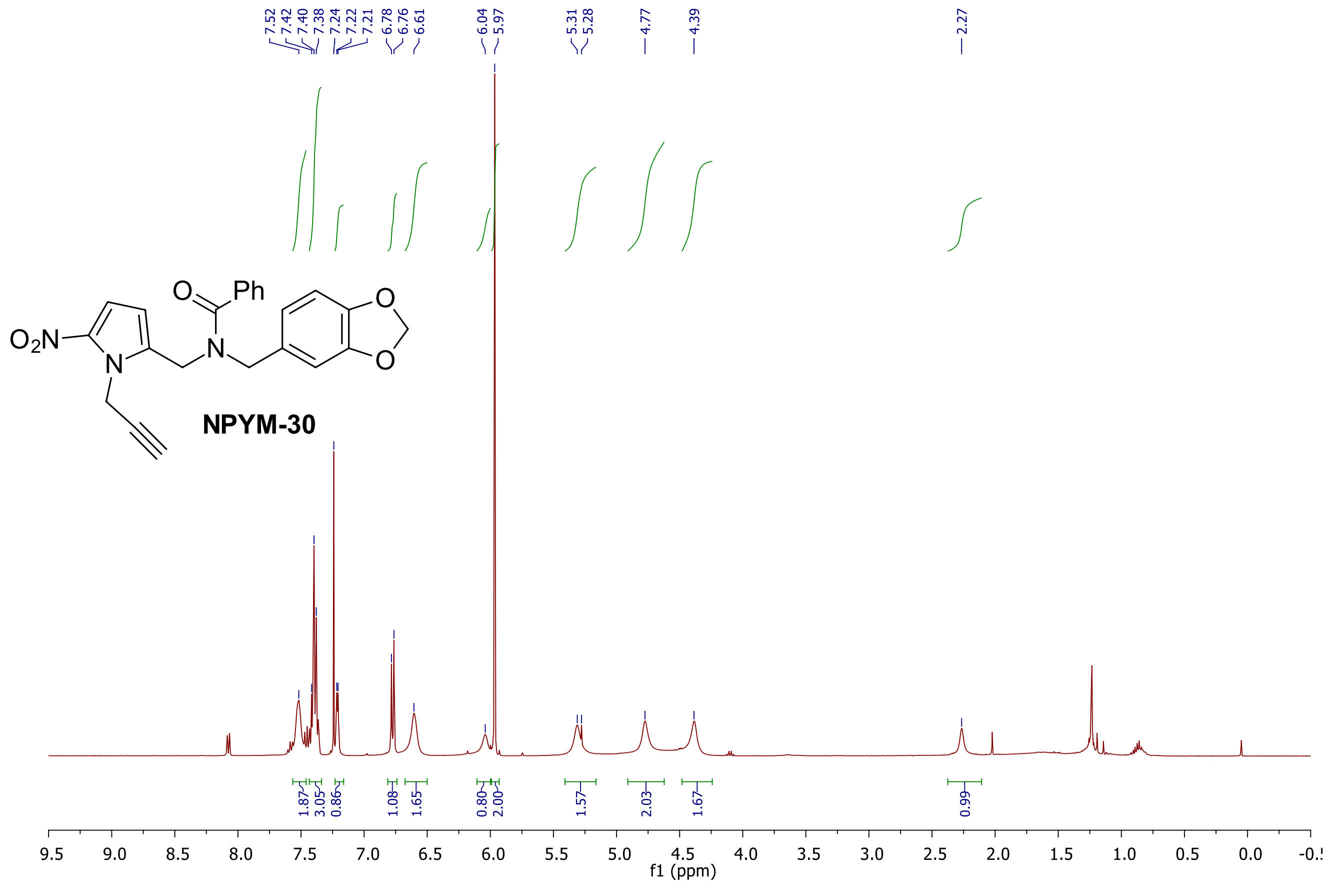
7.52 7.42 7.40 7.38 7.24 7.22 7.21 6.78 6.76 6.61 6.04 5.97 5.31 5.28 4.77 4.39 2.27
1.87 3.05 0.86 1.08 1.65 0.80 2.00 1.57 2.03 1.67 0.99
f1 (ppm)

NPYM-30

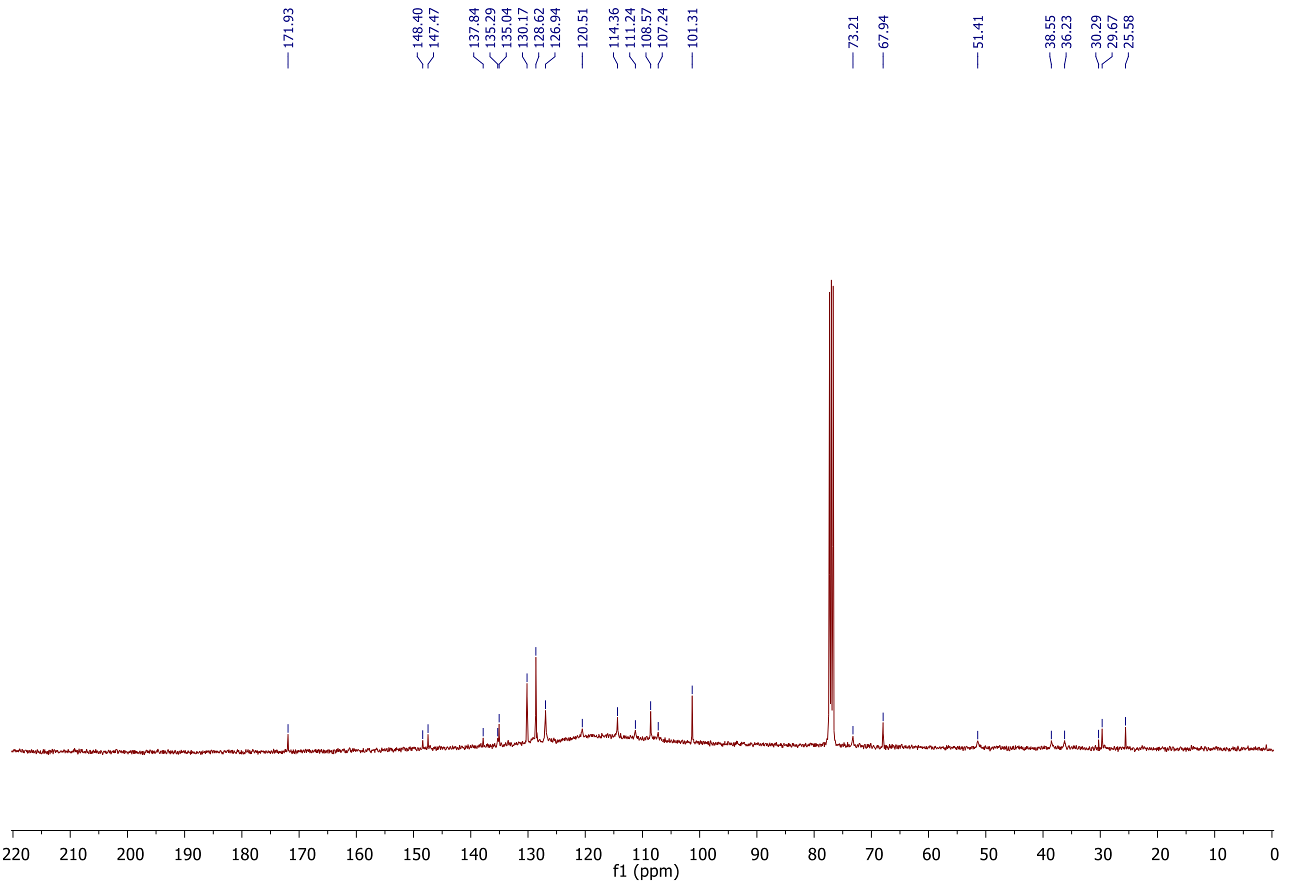
171.93 148.40 147.47 137.84 135.29 135.04 130.17 128.62 126.94 120.51 114.36 111.24 108.57 107.24 101.31 73.21 67.94 51.41 38.55 36.23 30.29 29.67 25.58
f1 (ppm)

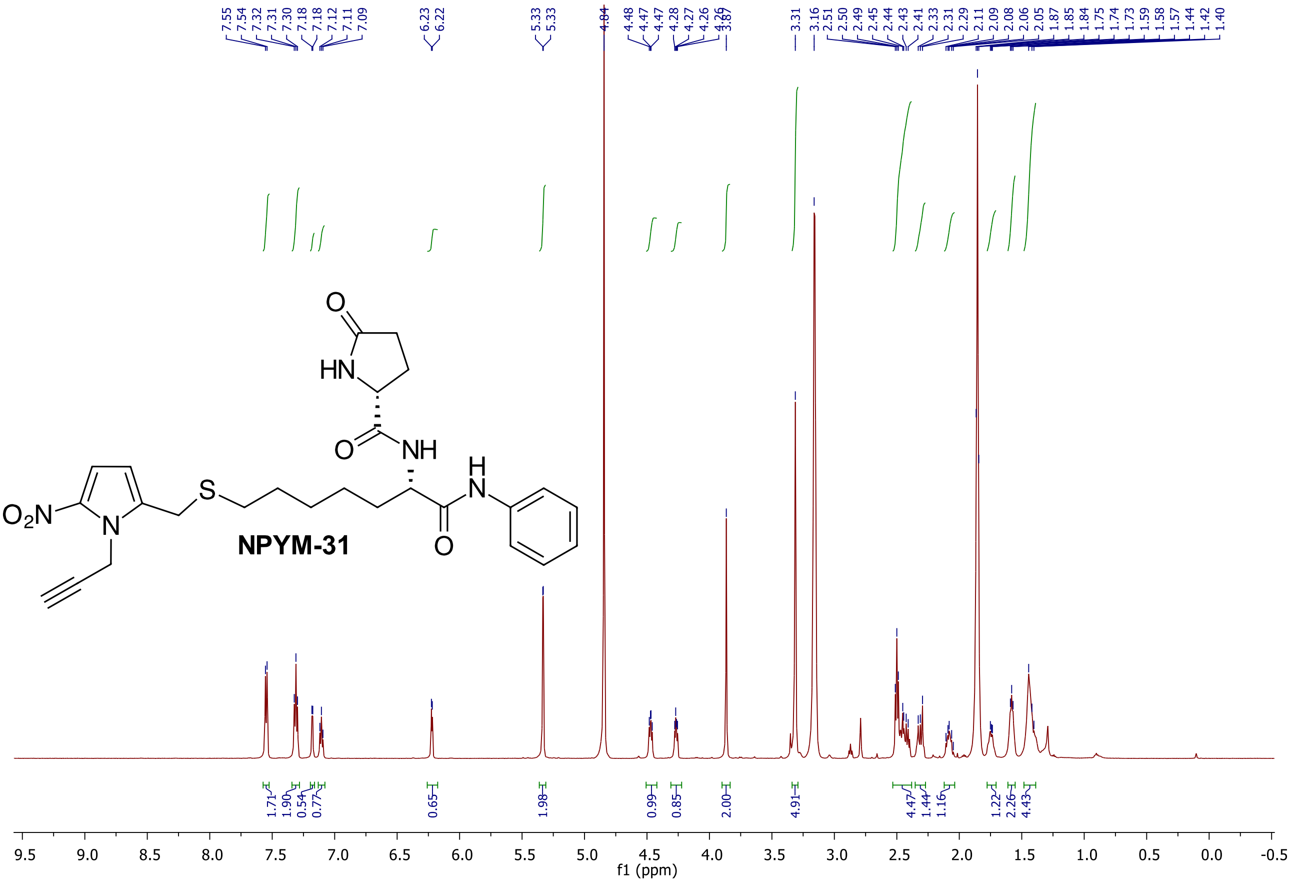

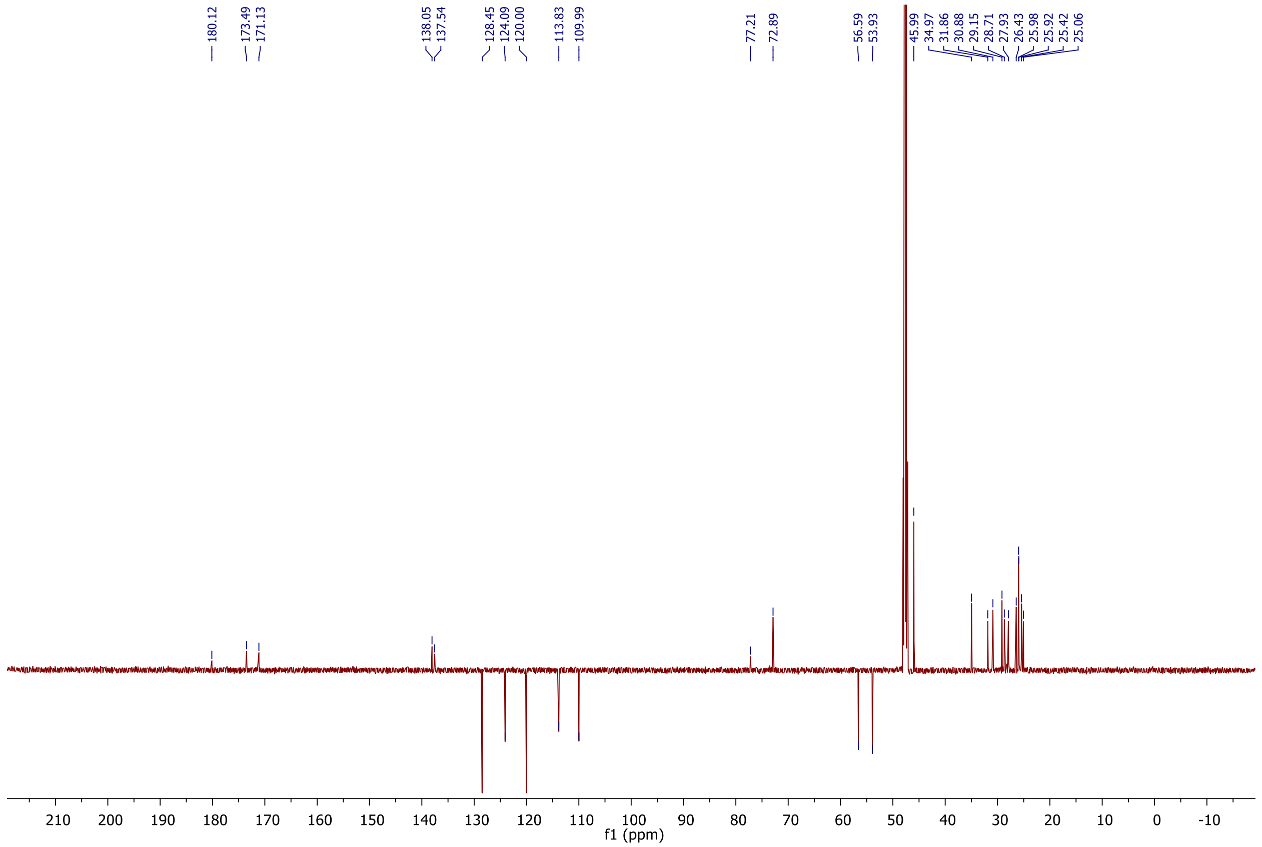

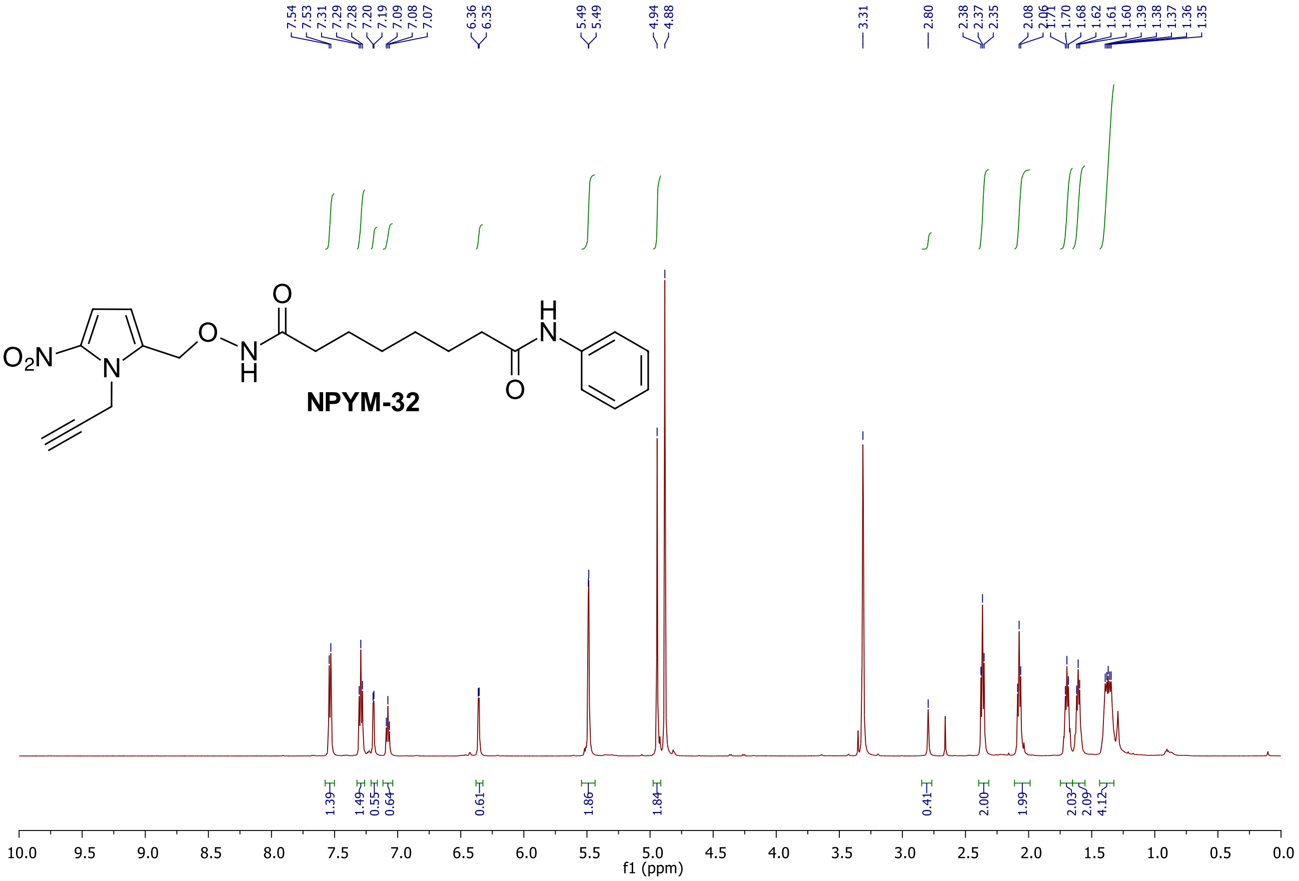
7.54
7.53
7.31
7.29
7.28
7.20
7.19
7.09
7.08
7.07
6.36
6.35
5.49
5.49
4.94
4.88
3.31
2.80
2.38
2.37
2.35
2.08
2.06
1.71
1.70
1.68
1.62
1.61
1.60
1.39
1.38
1.37
1.36
1.35
O2N
N
O
N
H
O
H
N
O
NPYM-32
1.39
1.49
0.55
0.64
0.61
1.86
1.84
0.41
2.00
1.99
2.03
2.09
4.12
f1 (ppm)

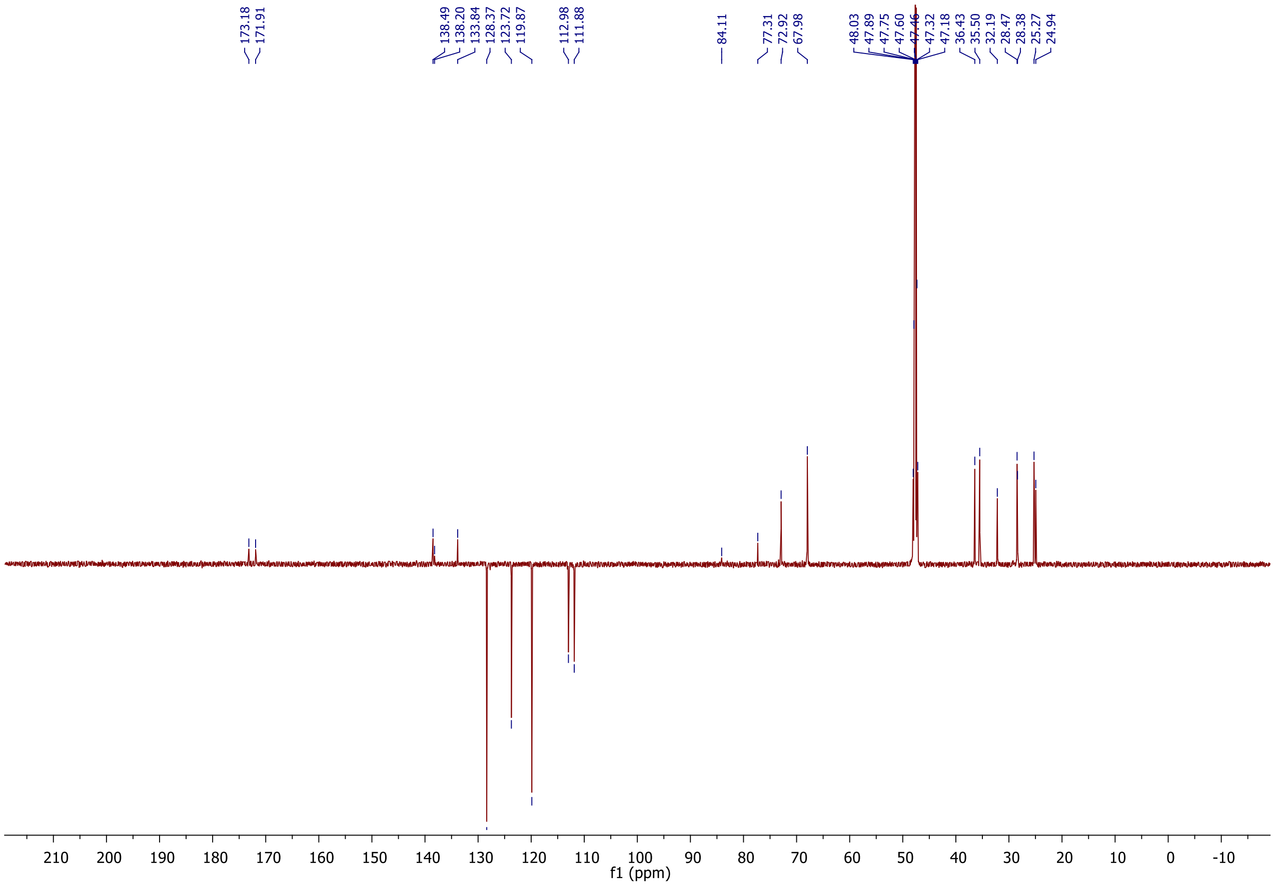
173.18
171.91
138.49
138.20
133.84
128.37
123.72
119.87
112.98
111.88
84.11
77.31
72.92
67.98
48.03
47.89
47.75
47.60
47.46
47.32
47.18
36.43
35.50
32.19
28.47
28.38
25.27
24.94
f1 (ppm)

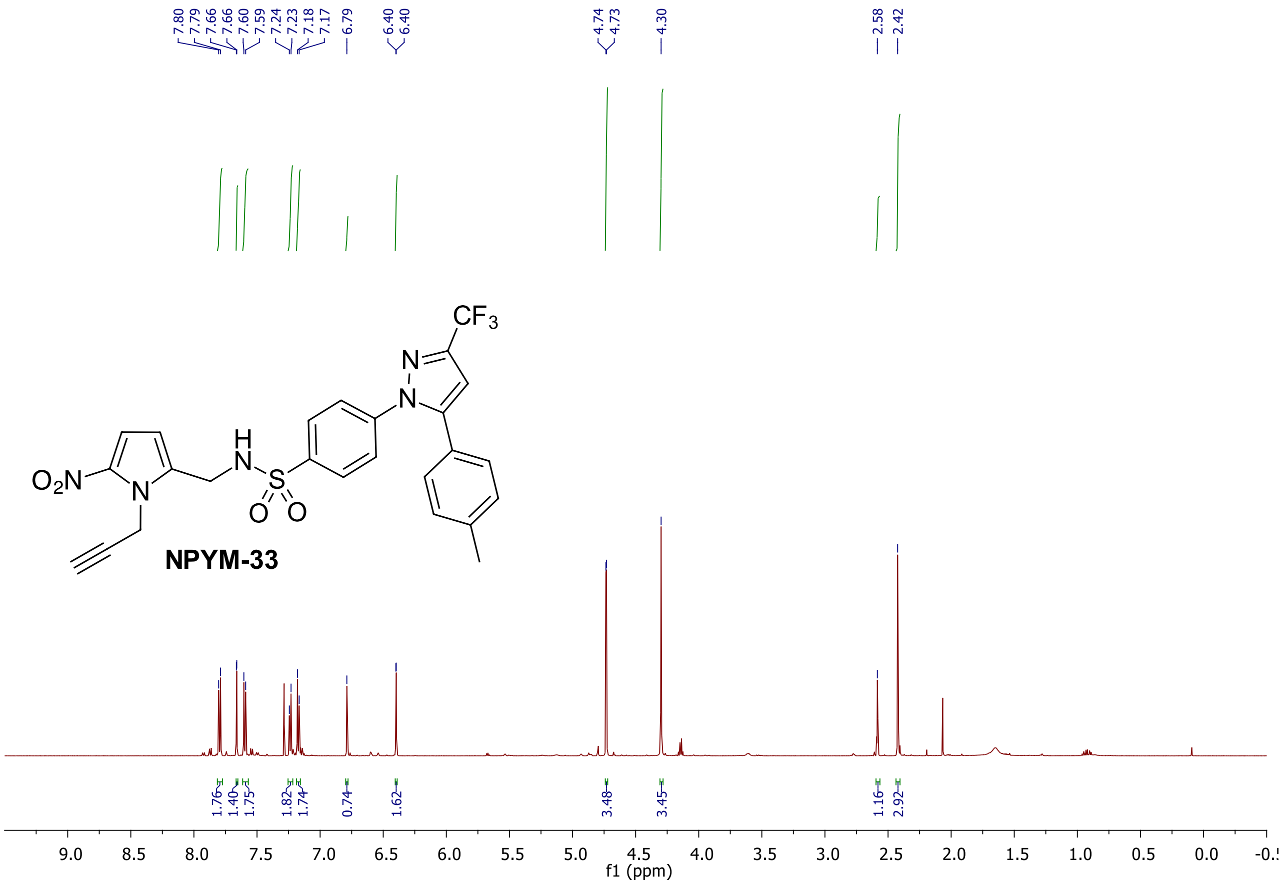
7.80
7.79
7.66
7.66
7.60
7.59
7.24
7.23
7.18
7.17
6.79
6.40
6.40
4.74
4.73
4.30
2.58
2.42
CF3
N
N
H
N
S
O
O
O2N
N
NPYM-33
1.76
1.40
1.75
1.82
1.74
0.74
1.62
3.48
3.45
1.16
2.92
9.0
8.5
8.0
7.5
7.0
6.5
6.0
5.5
5.0
4.5
4.0
3.5
3.0
2.5
2.0
1.5
1.0
0.5
0.0
f1 (ppm)

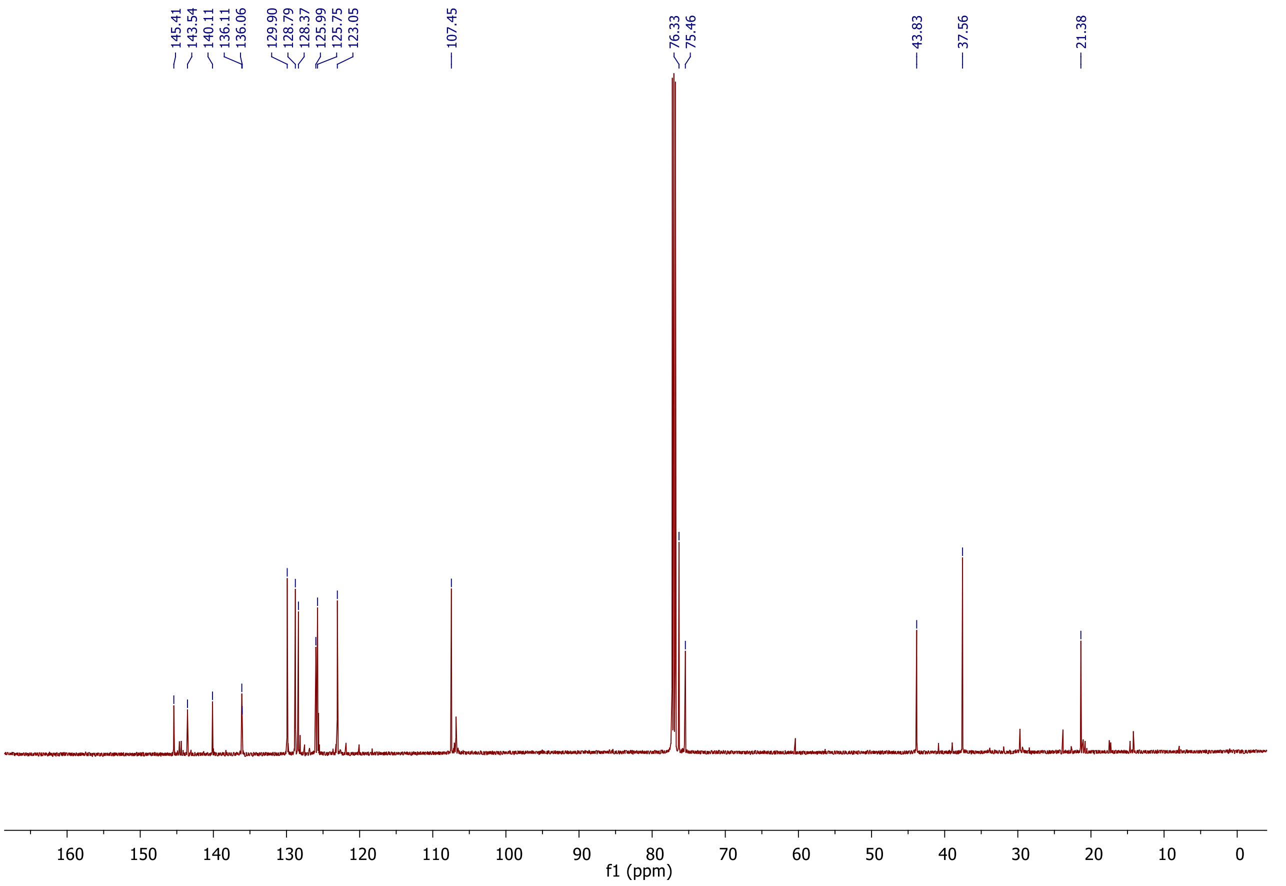
145.41
143.54
140.11
136.11
136.06
129.90
128.79
128.37
125.99
125.75
123.05
107.45
76.33
75.46
43.83
37.56
21.38
160
150
140
130
120
110
100
90
80
70
60
50
40
30
20
10
0
f1 (ppm)

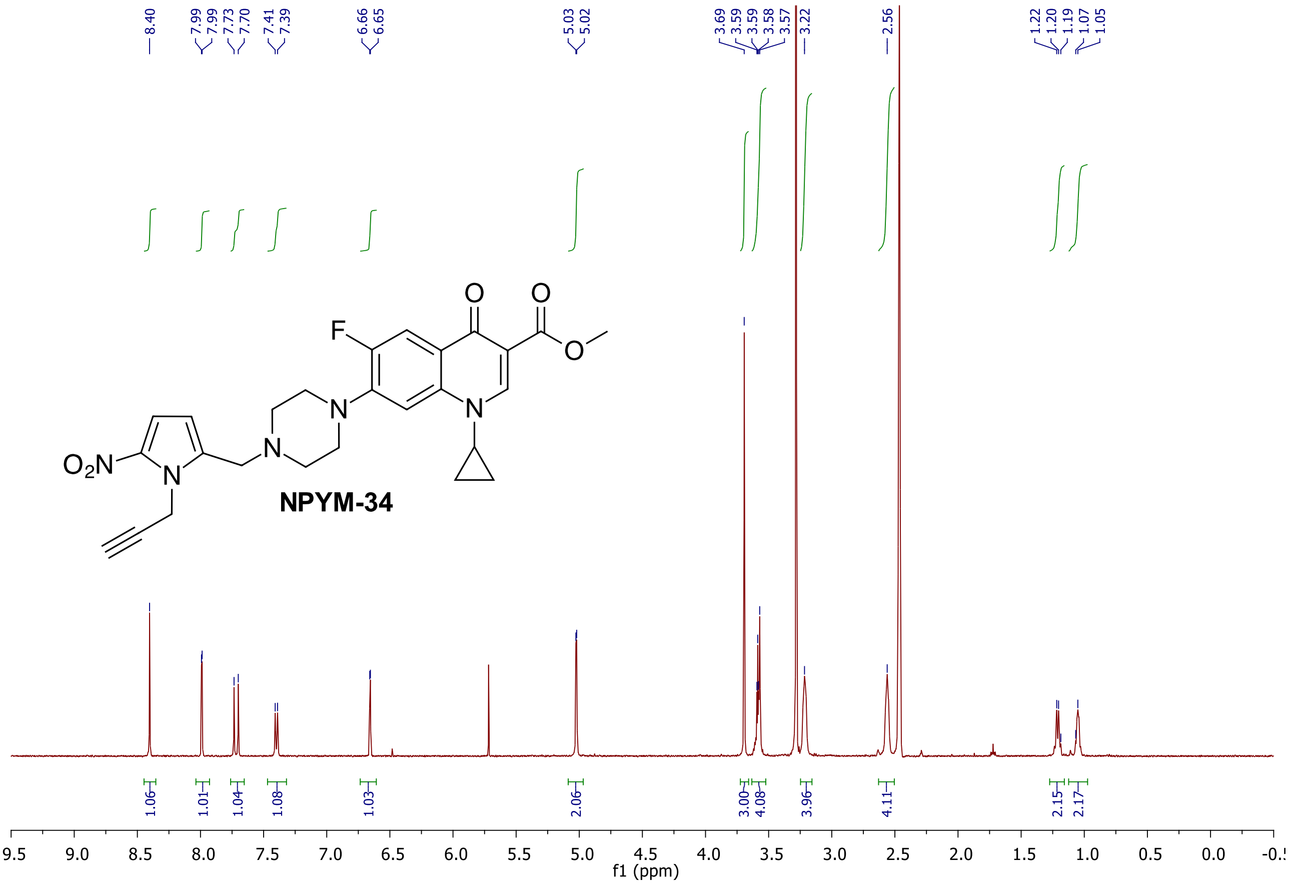
8.40
7.99
7.99
7.73
7.70
7.41
7.39
6.66
6.65
5.03
5.02
3.69
3.59
3.59
3.58
3.57
3.22
2.56
1.22
1.20
1.19
1.07
1.05
NPYM-34
1.06
1.01
1.04
1.08
1.03
2.06
3.00
4.08
3.96
4.11
2.15
2.17
f1 (ppm)

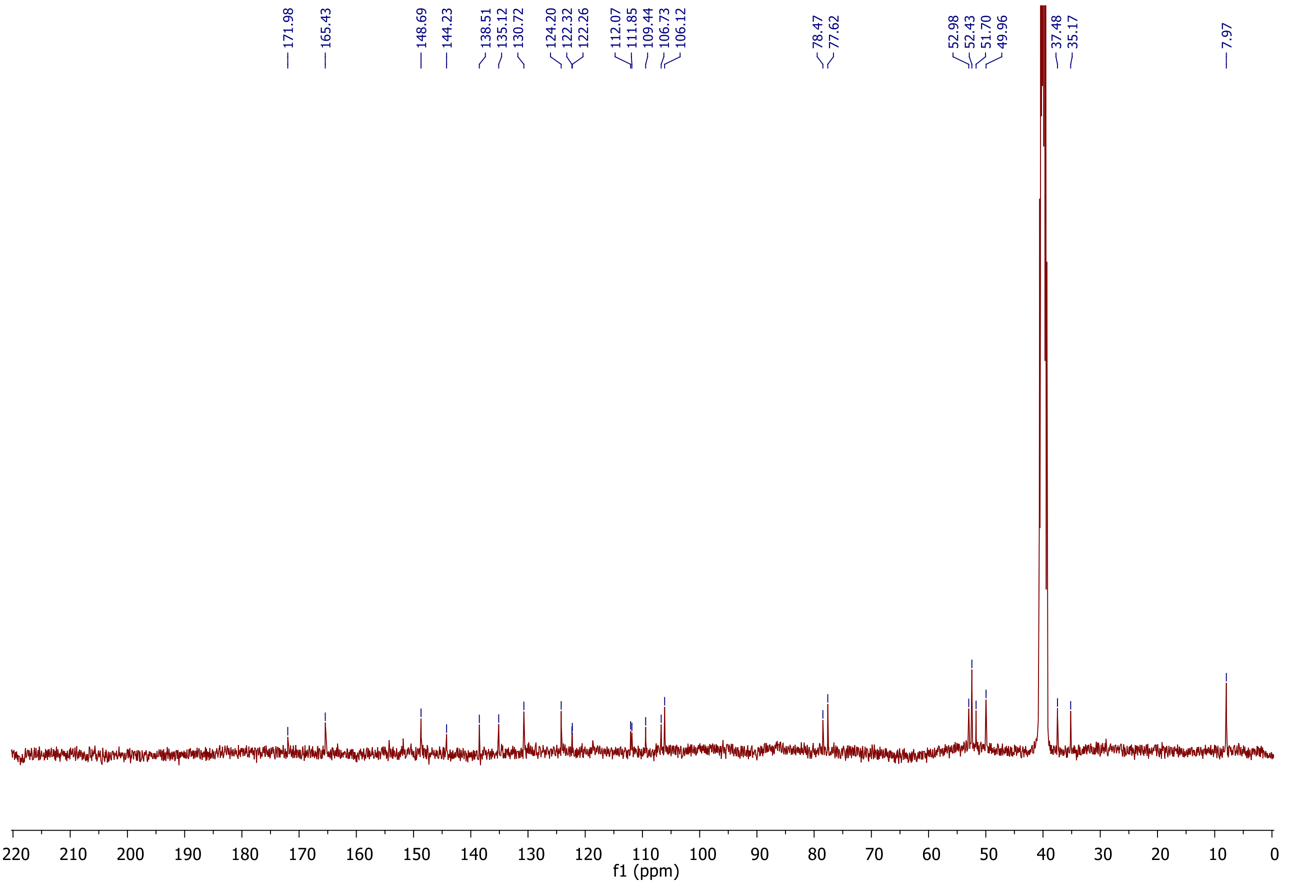
171.98
165.43
148.69
144.23
138.51
135.12
130.72
124.20
122.32
122.26
112.07
111.85
109.44
106.73
106.12
78.47
77.62
52.98
52.43
51.70
49.96
37.48
35.17
7.97
f1 (ppm)

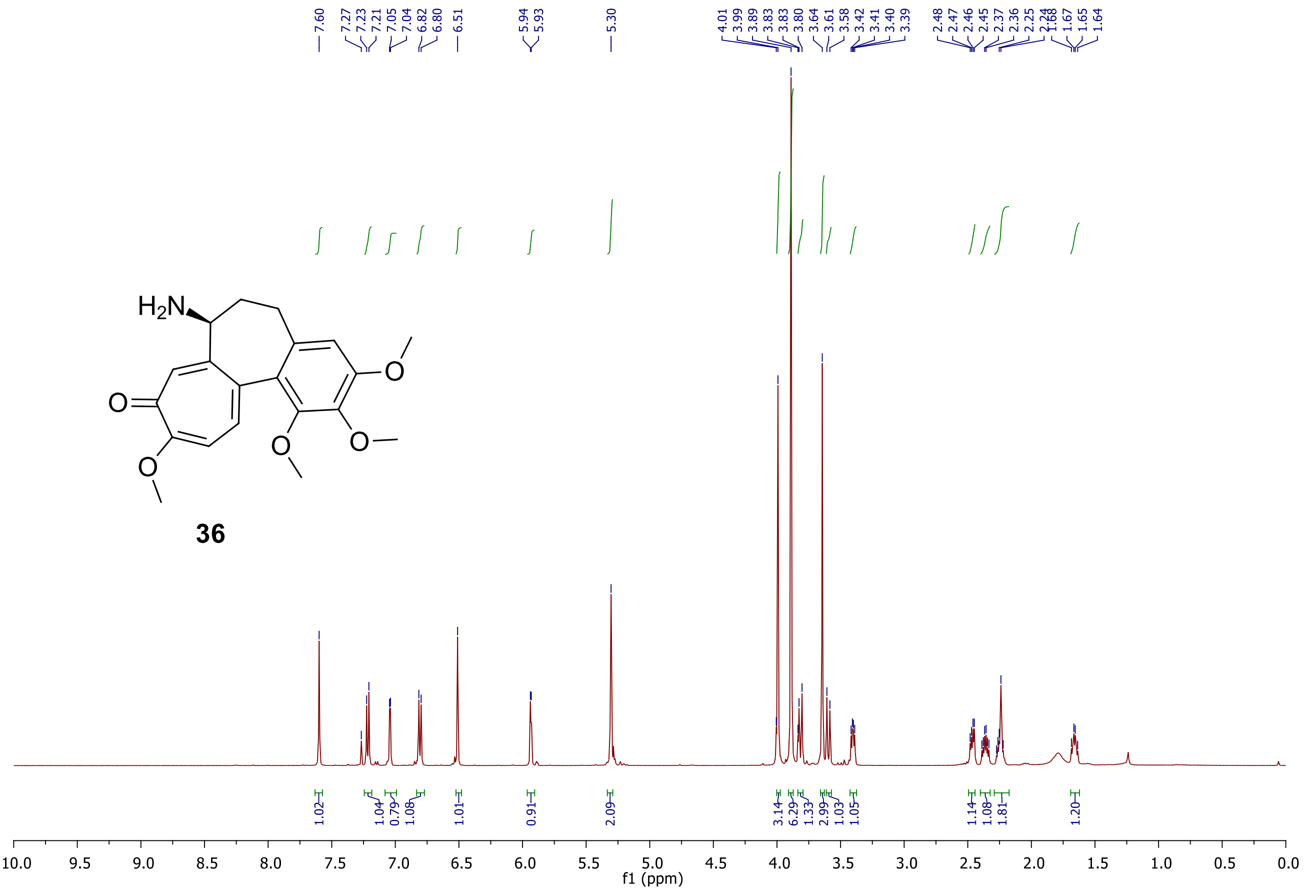
H2N
O
O
O
O
O
36
7.60 7.27 7.23 7.21 7.05 7.04 6.82 6.80 6.51 5.94 5.93 5.30 4.01 3.99 3.89 3.83 3.83 3.80 3.64 3.61 3.58 3.42 3.41 3.40 3.39 2.48 2.47 2.46 2.45 2.37 2.36 2.25 2.24 1.68 1.67 1.65 1.64
1.02 1.04 0.79 1.08 1.01 0.91 2.09 3.14 6.29 1.33 2.99 1.03 1.05 1.14 1.08 1.81 1.20
10.0 9.5 9.0 8.5 8.0 7.5 7.0 6.5 6.0 5.5 5.0 4.5 4.0 3.5 3.0 2.5 2.0 1.5 1.0 0.5 0.0
f1 (ppm)

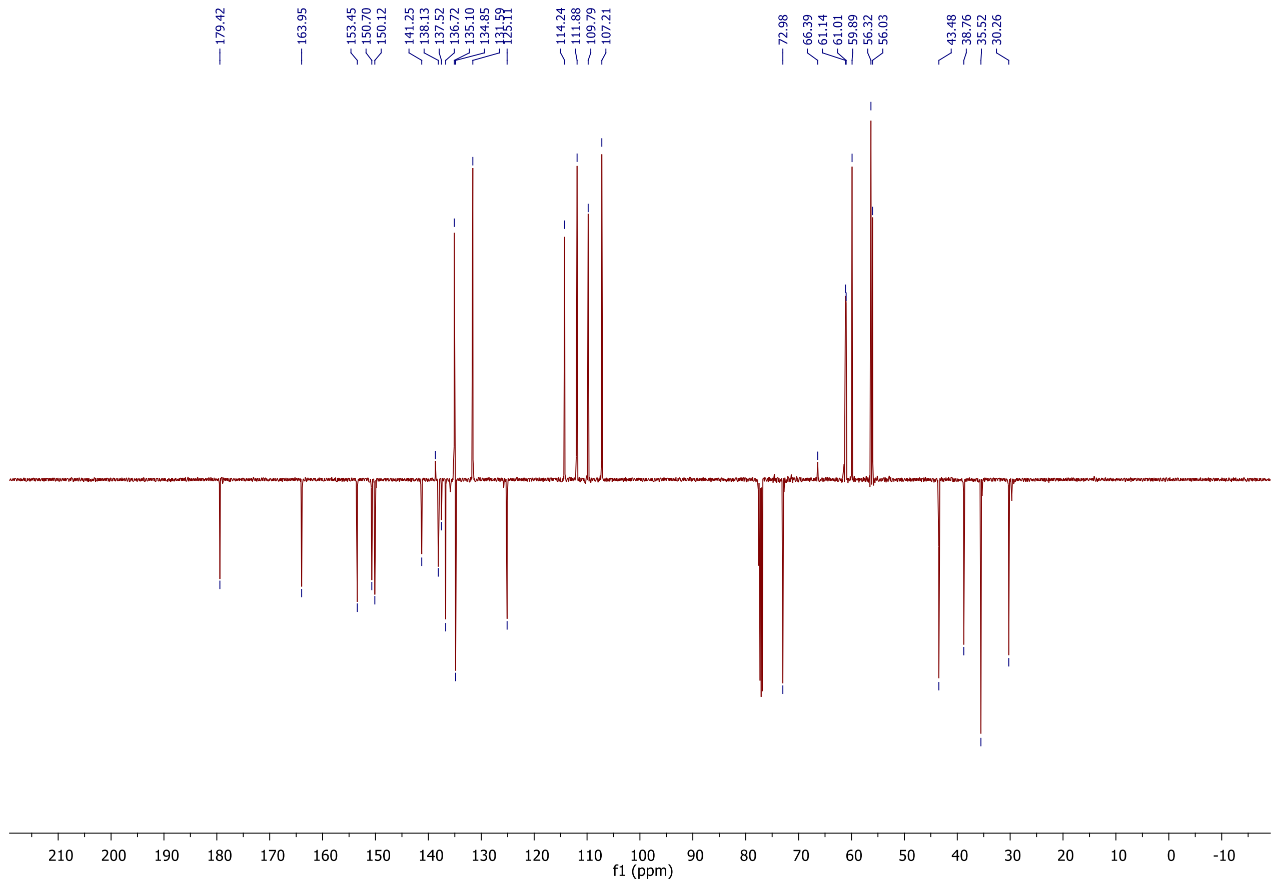
179.42 163.95 153.45 150.70 150.12 141.25 138.13 137.52 136.72 135.10 134.85 131.59 125.11 114.24 111.88 109.79 107.21 72.98 66.39 61.14 61.01 59.89 56.32 56.03 43.48 38.76 35.52 30.26
210 200 190 180 170 160 150 140 130 120 110 100 90 80 70 60 50 40 30 20 10 0 -10
f1 (ppm)

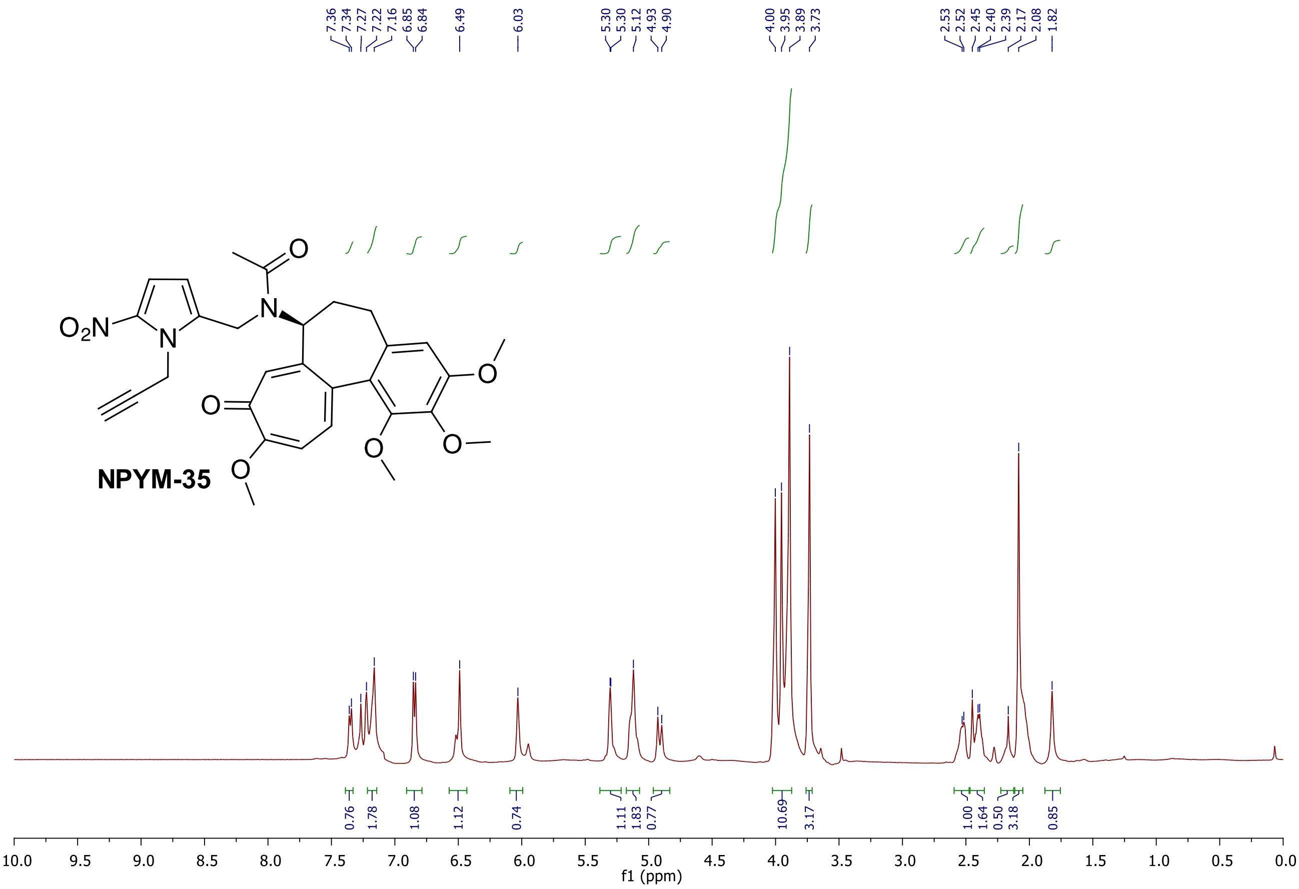

7.36
7.34
7.27
7.22
7.16
6.85
6.84
6.49
6.03
5.30
5.30
5.12
4.93
4.90
4.00
3.95
3.89
3.73
2.53
2.52
2.45
2.40
2.39
2.17
2.08
1.82
O_2N
N
O
NPYM-35
0.76
1.78
1.08
1.12
0.74
1.11
1.83
0.77
10.69
3.17
1.00
1.64
0.50
3.18
0.85
10.0 9.5 9.0 8.5 8.0 7.5 7.0 6.5 6.0 5.5 5.0 4.5 4.0 3.5 3.0 2.5 2.0 1.5 1.0 0.5 0.0
f1 (ppm)

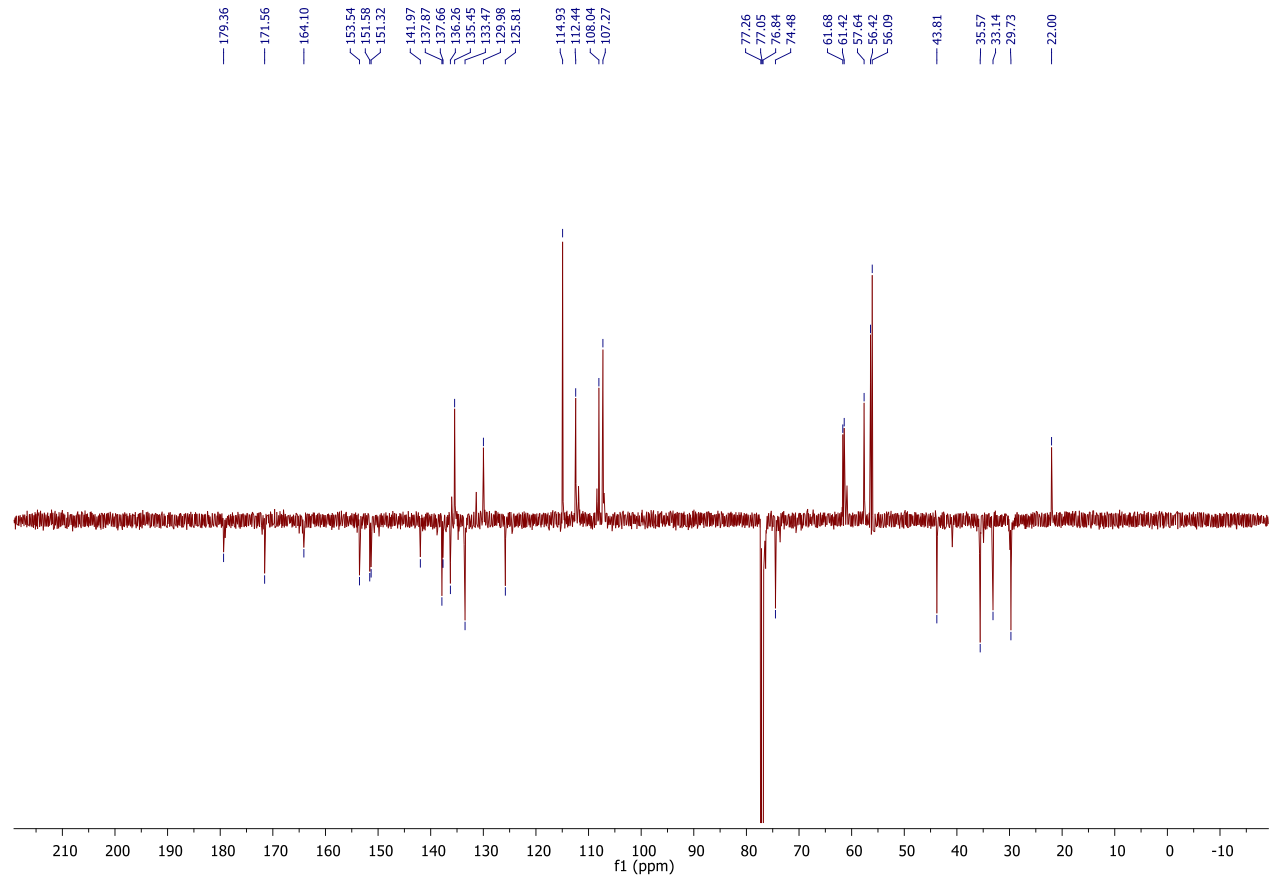

179.36
171.56
164.10
153.54
151.58
151.32
141.97
137.87
137.66
136.26
135.45
133.47
129.98
125.81
114.93
112.44
108.04
107.27
77.26
77.05
76.84
74.48
61.68
61.42
57.64
56.42
56.09
43.81
35.57
33.14
29.73
22.00
210 200 190 180 170 160 150 140 130 120 110 100 90 80 70 60 50 40 30 20 10 0 -10
f1 (ppm)

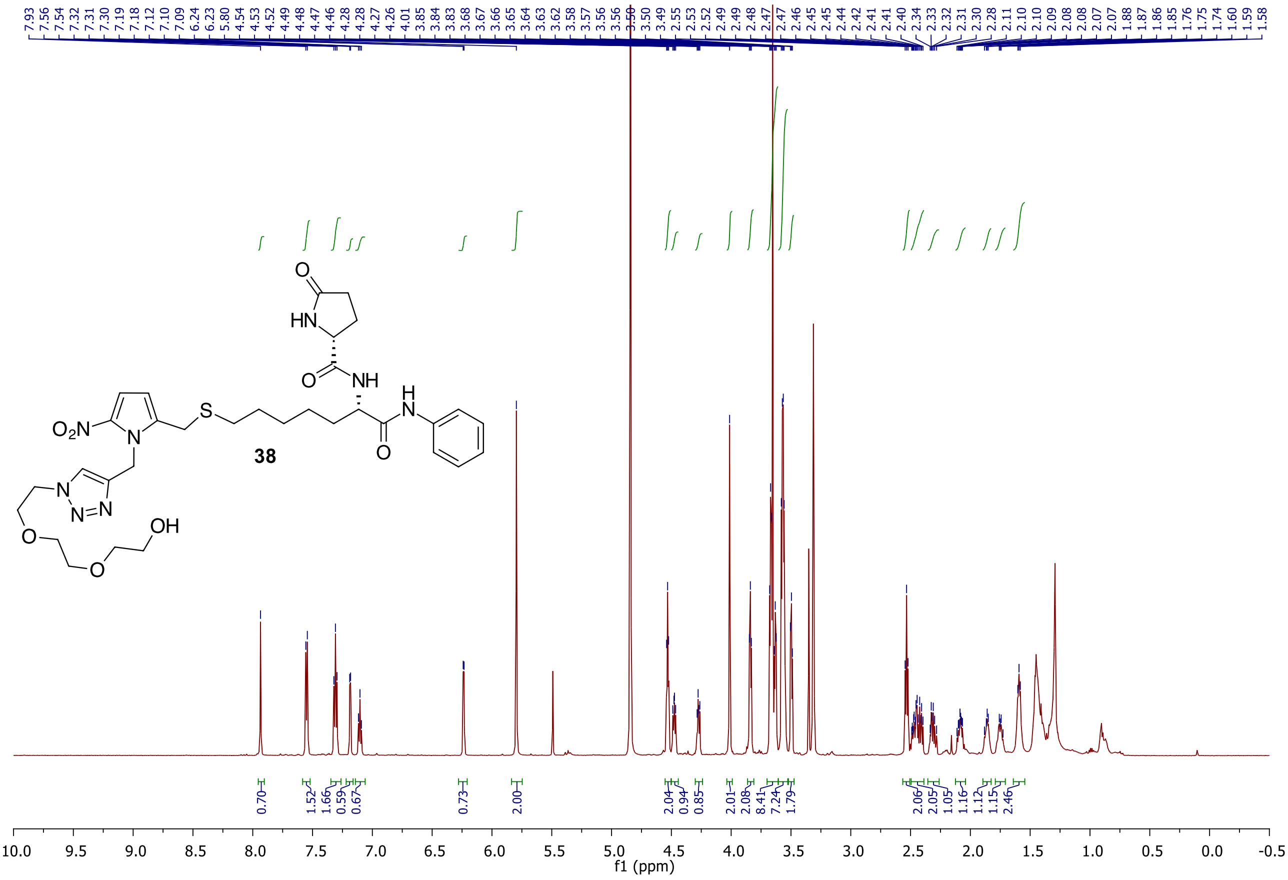
7.93 7.56 7.54 7.32 7.31 7.30 7.19 7.18 7.12 7.10 7.09 6.24 6.23 5.80 4.54 4.53 4.52 4.49 4.48 4.47 4.46 4.28 4.28 4.27 4.26 4.01 3.85 3.84 3.83 3.68 3.67 3.66 3.65 3.64 3.63 3.62 3.58 3.57 3.56 3.56 3.50 3.50 3.49 2.55 2.53 2.52 2.49 2.48 2.47 2.47 2.46 2.45 2.45 2.44 2.42 2.41 2.41 2.40 2.34 2.33 2.32 2.31 2.30 2.28 2.11 2.10 2.09 2.08 2.08 2.07 2.07 1.88 1.87 1.86 1.85 1.76 1.75 1.74 1.60 1.59 1.58
O
HN
O
NH
H
N
O
O2N
N
S
38
N
N=N
O
OH
O
0.70 1.52 1.66 0.59 0.67 0.73 2.00 2.04 0.94 0.85 2.01 2.08 8.41 7.24 1.79 2.06 2.05 1.05 1.16 1.12 1.15 2.46
10.0 9.5 9.0 8.5 8.0 7.5 7.0 6.5 6.0 5.5 5.0 4.5 4.0 3.5 3.0 2.5 2.0 1.5 1.0 0.5 0.0 -0.5
f1 (ppm)

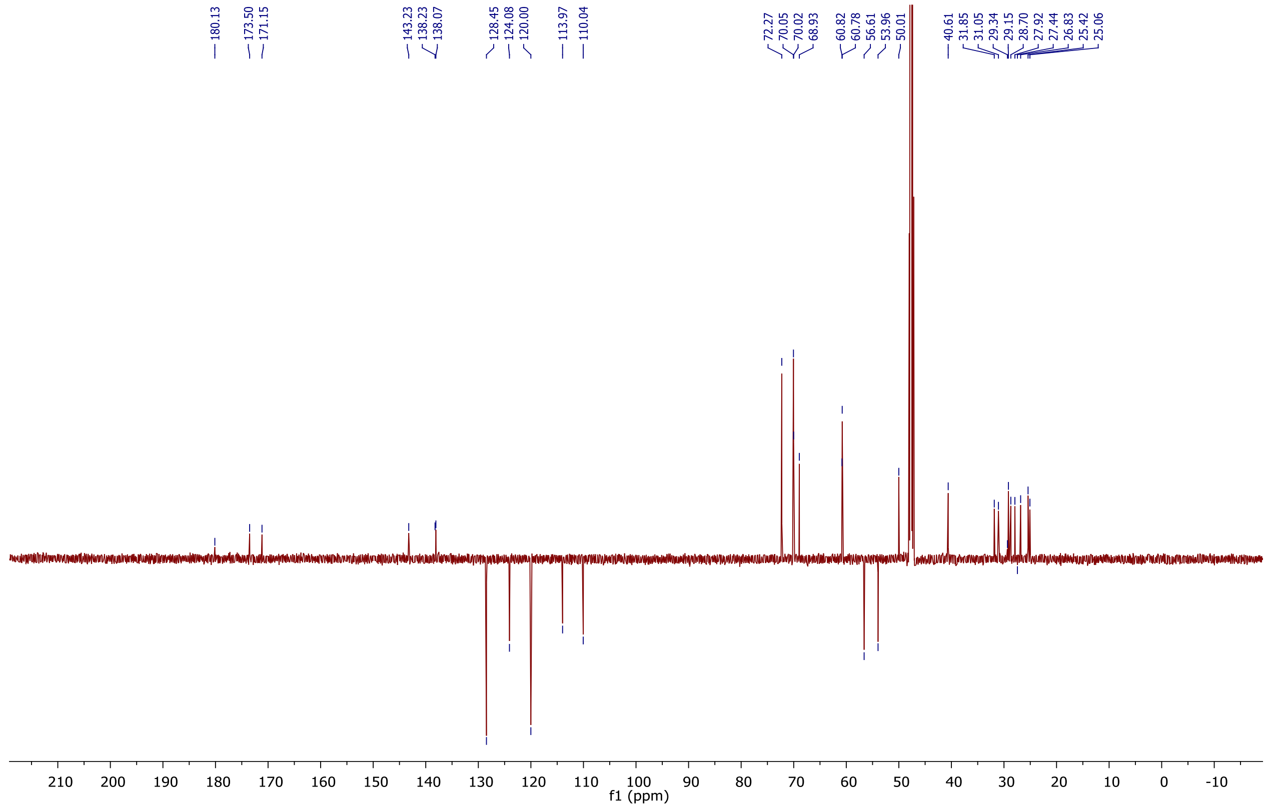
180.13 173.50 171.15 143.23 138.23 138.07 128.45 124.08 120.00 113.97 110.04 72.27 70.05 70.02 68.93 60.82 60.78 56.61 53.96 50.01 40.61 31.85 31.05 29.34 29.15 28.70 27.92 27.44 26.83 25.42 25.06
210 200 190 180 170 160 150 140 130 120 110 100 90 80 70 60 50 40 30 20 10 0 -10
f1 (ppm)

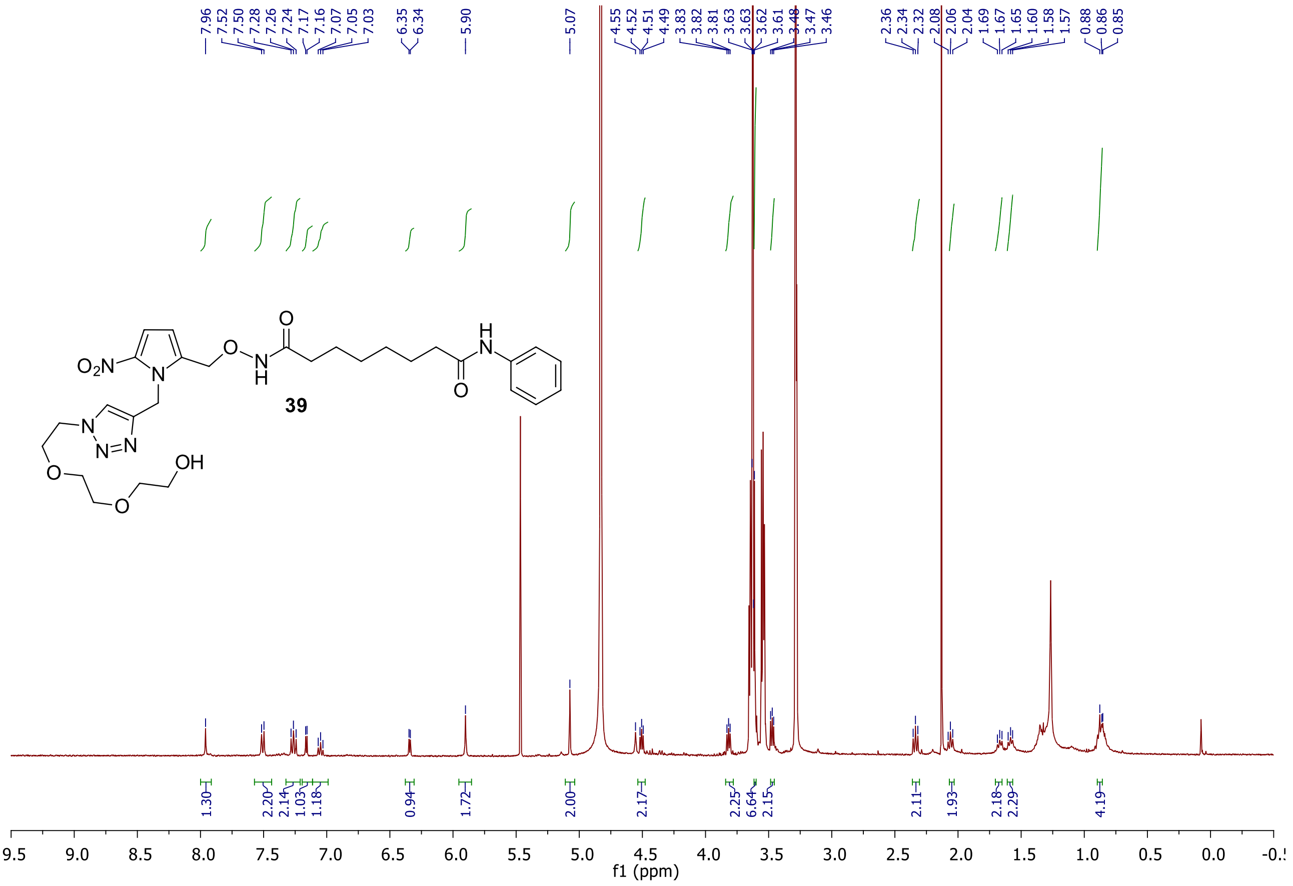
O$_2$N
N
O
N
H
O
H
N
O
39
N
N=N
O
O
OH
f1 (ppm)

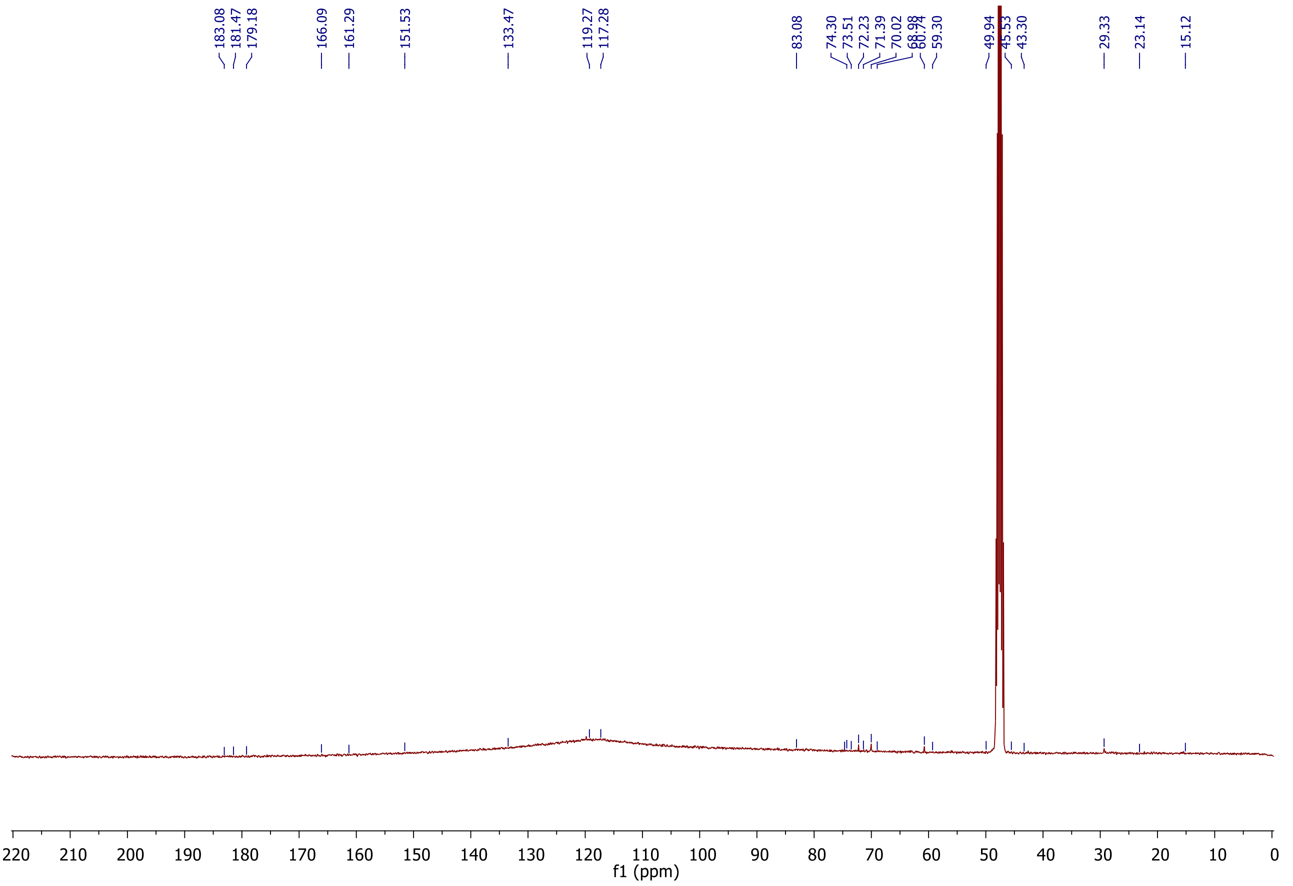
f1 (ppm)

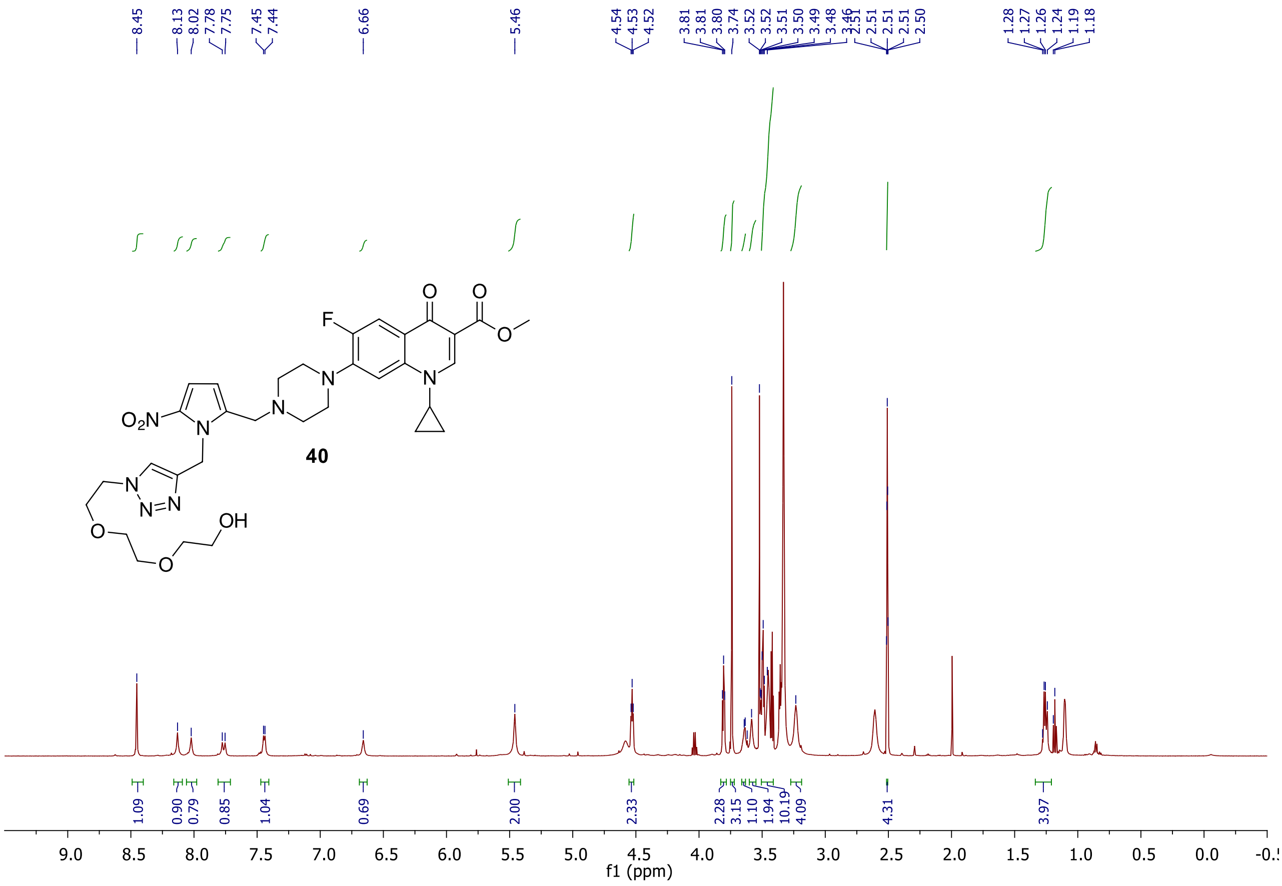
8.45
8.13
8.02
7.78
7.75
7.45
7.44
6.66
5.46
4.54
4.53
4.52
3.81
3.81
3.80
3.74
3.52
3.52
3.51
3.50
3.49
3.48
3.46
3.51
2.51
2.51
2.51
2.50
1.28
1.27
1.26
1.24
1.19
1.18
F
O
O
O
N
N
N
O2N
N
N
N=N
O
O
OH
40
1.09
0.90
0.79
0.85
1.04
0.69
2.00
2.33
2.28
3.15
1.10
1.94
10.19
4.09
4.31
3.97
f1 (ppm)

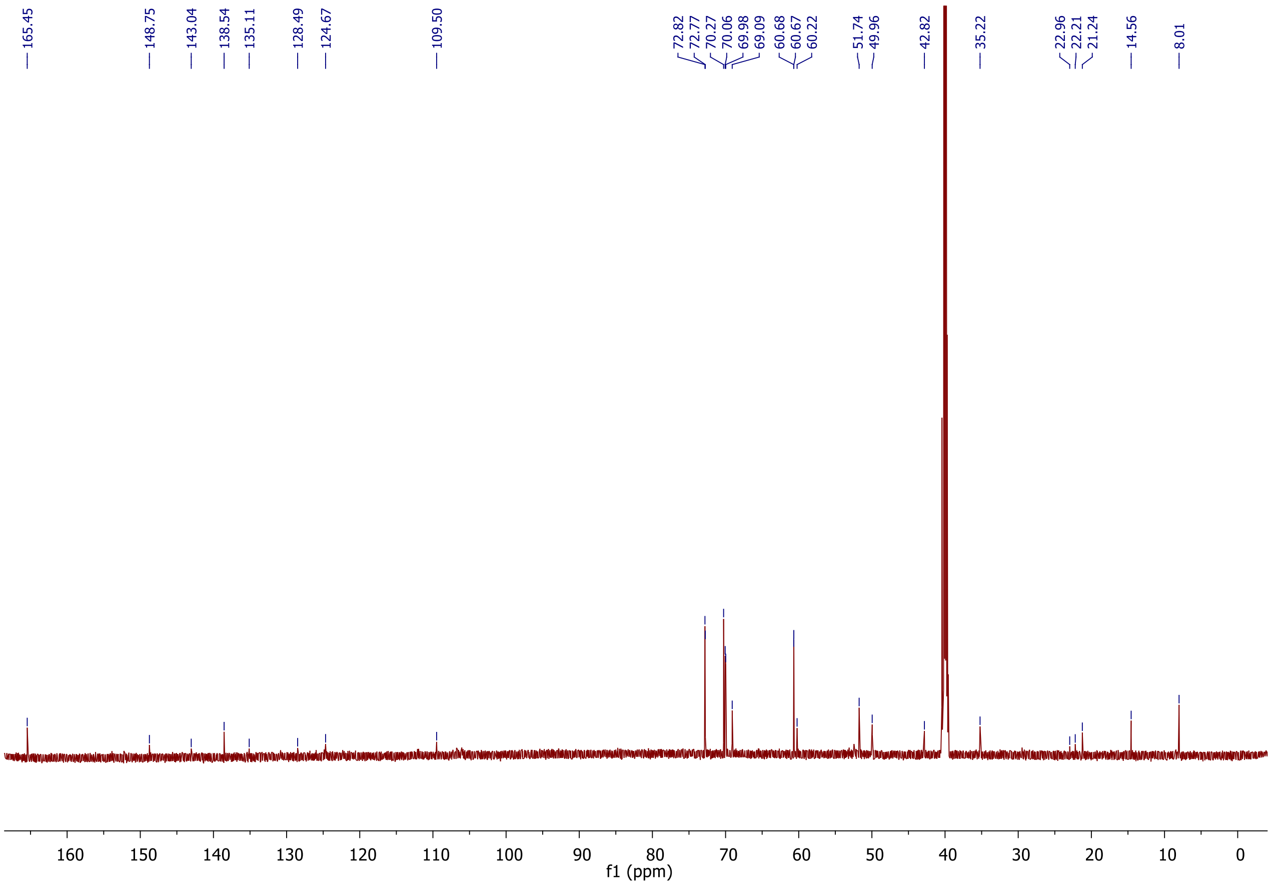
165.45
148.75
143.04
138.54
135.11
128.49
124.67
109.50
72.82
72.77
70.27
70.06
69.98
69.09
60.68
60.67
60.22
51.74
49.96
42.82
35.22
22.96
22.21
21.24
14.56
8.01
f1 (ppm)

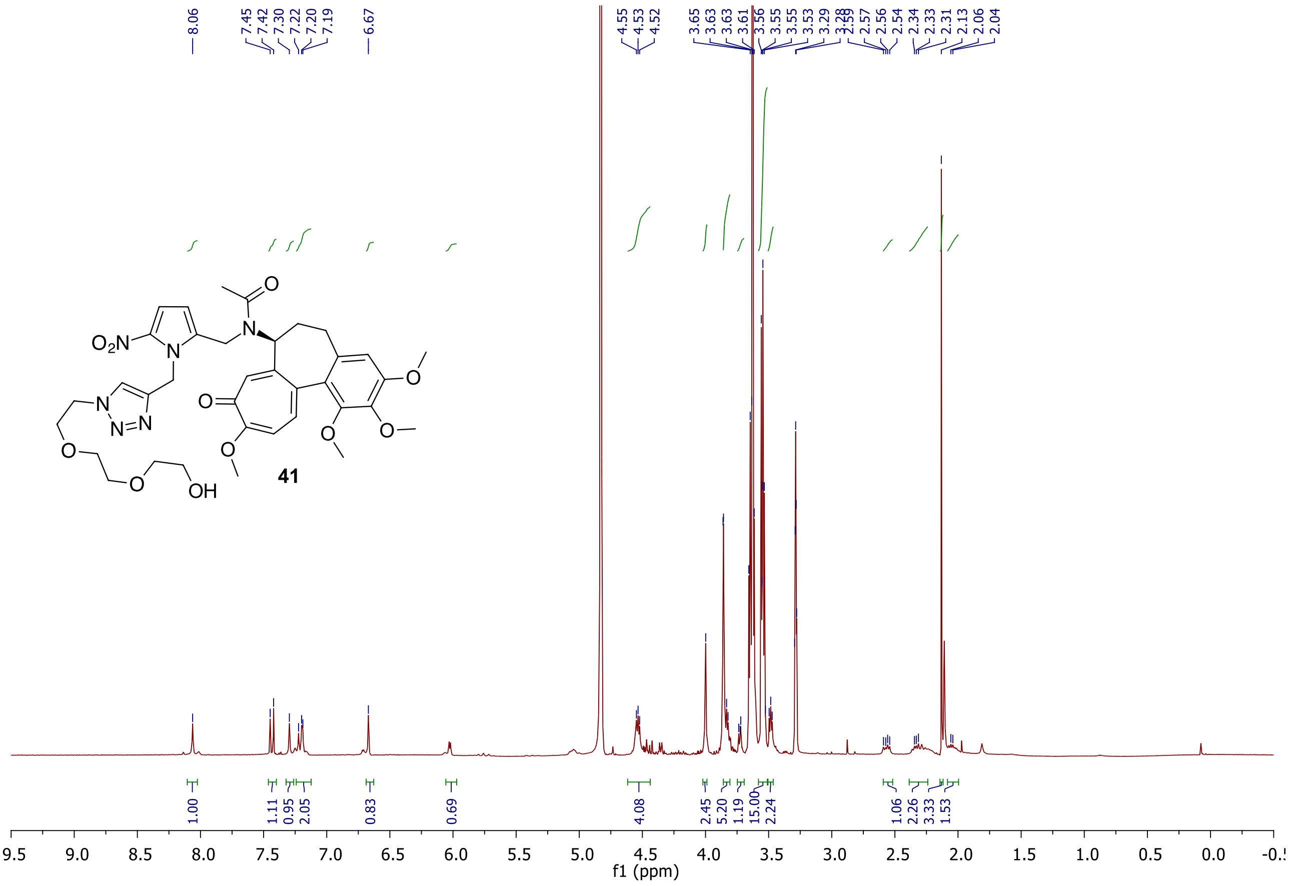
8.06
7.45
7.42
7.30
7.22
7.20
7.19
6.67
4.55
4.53
4.52
3.65
3.63
3.63
3.61
3.56
3.55
3.55
3.53
3.29
3.28
2.59
2.57
2.56
2.54
2.34
2.33
2.31
2.13
2.06
2.04
O₂N
N
O
OH
41
1.00
1.11
0.95
2.05
0.83
0.69
4.08
2.45
5.20
1.19
15.00
2.24
1.06
2.26
3.33
1.53
9.5 9.0 8.5 8.0 7.5 7.0 6.5 6.0 5.5 5.0 4.5 4.0 3.5 3.0 2.5 2.0 1.5 1.0 0.5 0.0 -0.5
f1 (ppm)

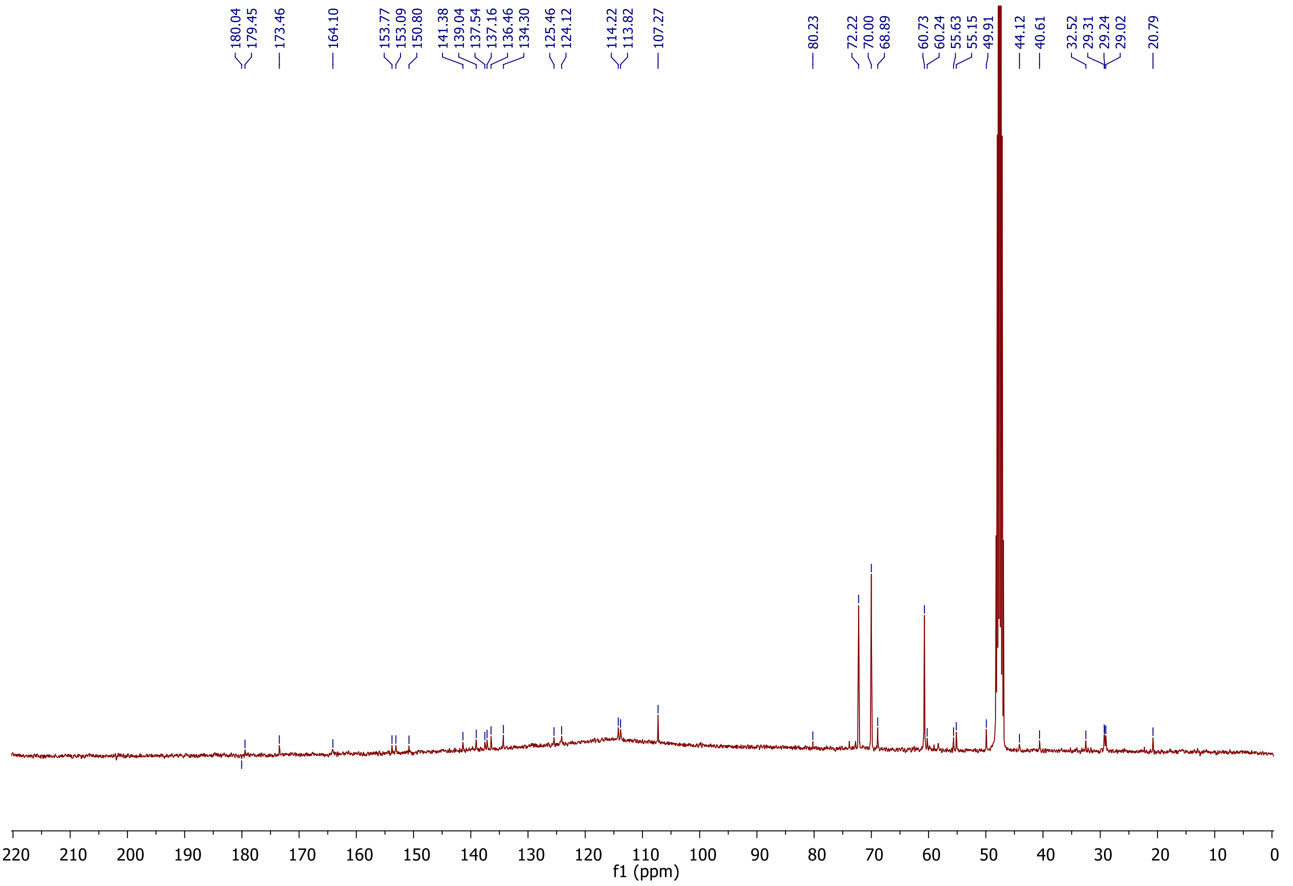
180.04
179.45
173.46
164.10
153.77
153.09
150.80
141.38
139.04
137.54
137.16
136.46
134.30
125.46
124.12
114.22
113.82
107.27
80.23
72.22
70.00
68.89
60.73
60.24
55.63
55.15
49.91
44.12
40.61
32.52
29.31
29.24
29.02
20.79
220 210 200 190 180 170 160 150 140 130 120 110 100 90 80 70 60 50 40 30 20 10 0
f1 (ppm)
